# Design, synthesis, molecular modeling, and bioactivity evaluation of 1,10-phenanthroline and prodigiosin (Ps) derivatives and their Copper(I) complexes against mTOR and HDAC enzymes as highly potent and effective new anticancer therapeutic drugs

**DOI:** 10.3389/fphar.2022.980479

**Published:** 2022-10-04

**Authors:** M. Mustafa Cetin, Wenjing Peng, Daniel Unruh, Michael F. Mayer, Yehia Mechref, Kemal Yelekci

**Affiliations:** ^1^ Department of Molecular Biology and Genetics, Faculty of Engineering and Natural Sciences, Kadir Has University, Istanbul, Turkey; ^2^ Department of Chemistry and Biochemistry, Texas Tech University, Lubbock, TX, United States

**Keywords:** breast cancer brain metastases, 1,10-phenanthroline, anticancer therapeutic drugs, mTOR, HDAC, HDAC1, molecular modeling, prodigiosin

## Abstract

Breast cancer is the second type of cancer with a high probability of brain metastasis and has always been one of the main problems of breast cancer research due to the lack of effective treatment methods. Demand for developing an effective drug against breast cancer brain metastasis and finding molecular mechanisms that play a role in effective treatment are gradually increasing. However, there is no effective anticancer therapeutic drug or treatment method specific to breast cancer, in particular, for patients with a high risk of brain metastases. It is known that mTOR and HDAC enzymes play essential roles in the development of breast cancer brain metastasis. Therefore, it is vital to develop some new drugs and conduct studies toward the inhibition of these enzymes that might be a possible solution to treat breast cancer brain metastasis. In this study, a series of 1,10-phenanthroline and Prodigiosin derivatives consisting of their copper(I) complexes have been synthesized and characterized. Their biological activities were tested *in vitro* on six different cell lines (including the normal cell line). To obtain additional parallel validations of the experimental data, some *in silico* modeling studies were carried out with mTOR and HDAC1 enzymes, which are very crucial drug targets, to discover novel and potent drugs for breast cancer and related brain metastases disease.

## Graphical Abstract

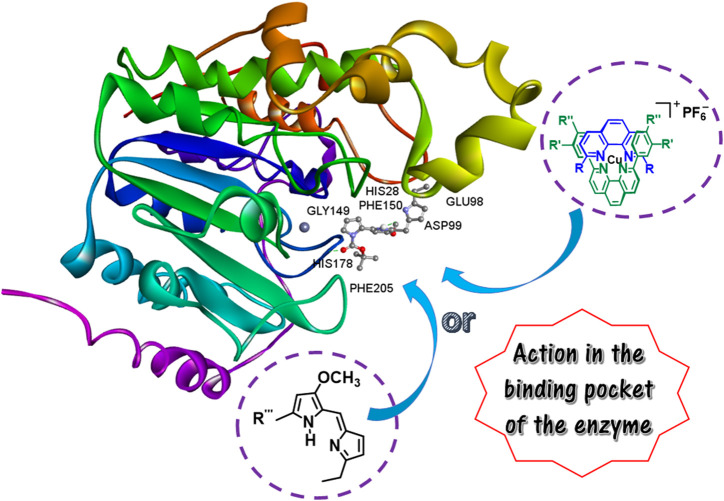

## Introduction

Cancer is the second leading cause of death for people, and about one in six attributed deaths is due to cancer (WHO, 2022). Breast cancer has been considered as the leading type of cancer within females and counts for 25% of all cases (IARC, 2014). This cancer type also has the secondary highest possibility of brain metastasis (Leone and Leone, 2015) following lung cancer. In 2012, 1.68 million breast cancer cases were diagnosed with more than 500,000 deaths (IARC, 2014). Approximately 10%–20% of breast cancer patients suffer from malignant brain metastasis (Pangeni et al., 2015). Due to the development of prognostic and diagnostic methods and the longer survival rate of primary breast cancer patients, this percentage is continuously increasing in recent years (Rostami et al., 2016). The mortality of breast cancer brain metastasis is currently viewed as one of the central themes in breast cancer research. Many efforts have been directed to the development of breast cancer therapeutics and prognostic tools (Lin et al., 2002; Kölbl et al., 2015). However, there is no efficient anticancer therapeutic/treatment specific for breast cancer, especially for those patients who have a high brain metastasis risk. Thus, there is an increasing demand for the development of effective breast cancer drug to inhibit the ezymes that play an important role on modulating breast cancer progression.

In the last decades, inhibition of histone deacetylases (HDAC) and mammalian target of rapamycin (mTOR) enzymes has emerged as a potential strategy (Yao et al., 2021) to reverse abnormal epigenetic changes associated with cancer, and several classes of HDAC and mTOR inhibitors have been found to have potent and specific anticancer activities in preclinical studies (Kawai et al., 2003; Fasolo and Sessa, 2012; Min et al., 2012; Tang et al., 2017; Bian et al., 2018; Guo et al., 2020; Yao et al., 2021). The targeted inhibition of HDAC as a potent anticancer therapy approach (Tang et al., 2017; Bian et al., 2018; Guo et al., 2020) and mTOR as a promising target for anticancer therapies due to its central role in the control of cancer cellular growth, division, survival, and angiogenesis and its frequent dysregulation in cancer cells are two of the very important potent anticancer therapy approaches. In particular, the better understanding of the mTOR chemical structure and pathway in return has led to developing mTOR inhibitors with different targets (Fasolo and Sessa, 2012). Therefore, most researchers in the field have focused their studies on developing new and different inhibitors targeting such enzymes, and actively been studying to elucidate the precise mechanism of action of such inhibition processes. For instance, Yao et al. (2021) have studied the biological evaluation of dual mTOR/HDAC6 inhibitors in MDA-MB-231 cells proposing that dual targeting mTOR and HDAC inhibitors is a promising strategy for triple negative breast cancer (TNBC) treatment. They have designed and synthesized a series of dual mTOR/HDAC6 inhibitors by structure-based strategy and found that one of the compounds was a potent dual mTOR/HDAC6 inhibitor with IC_50_ value of 133.7 nM against mTOR and 56 nM against HDAC6, presenting mediate antiproliferative activity in TNBC cells. They have also found to induce significant autophagy, apoptosis and suppress migration in MDA-MB-231 cells. Fasolo and Sessa (2012) have also shown that the mTOR is a protein kinase involved in the phosphatidylinositol 3-Kinase (PI3K)/AKT signalling pathway with a central role in the control of cell growth, survival, and angiogenesis, and multiple and frequent dysregulations of this pathway in human tumors make it a central target in the development of new anticancer treatments. Another study (Bian et al., 2018) has shown that HDAC inhibitor suppresses proliferation and invasion of breast cancer cells through regulation of miR-200c targeting CRKL, in which miR-200c was significantly downregulated in breast cancer cell lines compared to normal cell lines and inversely correlated with the levels of class IIa HDACs and CRKL, suggesting that the HDAC-miR200c-CRKL signaling axis could be a novel diagnostic marker and potential therapeutic target in breast cancer. Guo et al. (2020) also found that expression of HDAC1 and retinoblastoma binding protein 4 (RBBP4, playing an important role in transcription, cell cycle, and proliferation) correlate with clinicopathologic characteristics and prognosis in breast cancer *via* the performed immunohistochemistry on 240 breast cancer patients to assess such expression. It was found that HDAC1 and RBBP4 expression in breast cancer was significantly higher than that in normal tissues and claimed that the mechanism may be regulated transcription or translation of estrogen receptor (ER) and progesterone receptor (PR) by HDAC1. Kawai et al. (2003) have also worked on the overexpression of HDAC1 modulating breast cancer progression by negative regulation of estrogen receptor α (ER-α), which is a critical growth regulatory gene in breast cancer and its expression level is tightly linked to the prognosis and treatment outcomes of breast cancer patients (loss of ER-α expression in breast epithelial cells is critical for breast cancer progression). They have shown that HDAC1 interacts with ER-α *in vitro* and *in vivo* and suppresses its transcription activity, in which their findings strongly suggest that HDAC1 affects breast cancer progression by promoting cellular proliferation in association with a reduction in both ER-α protein expression and transcriptional activity. HDAC1 may thus be a potential target for therapeutic intervention in the treatment of a subset of ER-negative breast cancers, and inhibition of HDAC1 expression or activity may provide a new strategy for breast cancer therapy. In another study (Lu and Liu, 2020), Lu and Liu have reviewed the functions of ER-α and the mechanism of resistance to endocrine therapy as well as the recently reported potent selective ER Degraders (SERDs) that are promising strategy for ER positive endocrine-resistant breast cancer. With the emphasis on their diverse chemical structures and pharmacological profiles, they have categorized oral SERDs into five subtypes, such as steroidal, acrylic acid, basic amino, PROTAC, and long alkyl chain subtypes, which may provide new insights into the new treatment approaches for ER positive endocrine-resistant breast cancer. Similarly, Tang et al. has currently shown in their work (Tang et al., 2017) that HDAC1 triggers the proliferation and migration of breast cancer cells *via* upregulation of interleukin-8 (IL-8). Their data has shown that mRNA and protein levels of HDAC1 in 75% of the breast cancer cells are greater than that in their corresponding adjacent normal cells (fibroblast 3T3 and epithelial breast MCF10A). They have claimed that knockdown of HDAC1 by specific siRNAs can suppress the proliferation and migration of breast cancer cells and inhibit the expression of IL-8, suggesting that HDAC1 may be a potential therapy target for breast cancer treatment.

Along with the abovementioned literature studies, it can clearly be seen that there are many studies that show novel synthetic inhibitors for HDAC and mTOR enzyme inhibition that have been designed, synthesized, and examined for their antitumor and anticancer activities as well as their mechanisms of action. In one of these studies, inhibitors against HDAC for its antitumor activity and the underlying molecular mechanisms of such activity on MDA-MB-231 (231) human breast cancer cells have been examined, and the data from the study suggested that the HDAC inhibitor, namely IN-2001, is a novel promising therapeutic agent with potent antitumor effects against human breast cancers (Min et al., 2012). With the better acknowledgement and understanding of molecular and cellular mechanisms of HDAC and mTOR inhibition, studies in the field have been directed towards novel potent anticancer agents (Macleod, 1952; Butler et al., 1969; Dwyer et al., 1969; Ranford et al., 1993; Fricker, 1994; Zoroddu et al., 1996; Erkkila et al., 1999; Choi et al., 2001; Marzano et al., 2002; de Ruijter et al., 2003; Zhang and Lippard, 2003; Halkidou et al., 2004; Saha et al., 2004; Krusche et al., 2005; Heffeter et al., 2006; Walsh et al., 2006; Senese et al., 2007; Weichert, 2009; Espona-Fiedler et al., 2012; Müller et al., 2013; Danevčič et al., 2016; Wang et al., 2016; Uba and Yelekçi, 2017; Shouksmith et al., 2019).

In recent years, there have been a few reports highlighting the use of transition metal complexes as anti-cancer agents and their possible anti-cancer chemotherapeutic potential (Fricker, 1994; Lippard and Berg, 1994; Solomon et al., 1996; Zhang and Lippard, 2003; Heffeter et al., 2006; Que et al., 2008; Hanahan and Weinberg, 2011; Brady et al., 2014; Chang, 2015; Denoyer et al., 2015; Sun et al., 2022; Ge et al., 2022; Lu et al., 2022). Of these transition metals, copper in particular plays very important role in mammalian cells, e.g., copper homeostasis (Denoyer et al., 2015; Li, 2020; Ge et al., 2022), inhibition of colorectal cancer progression with diethyldithiocarbamate-copper complex (Huang et al., 2021), *in vitro* and *in vivo* use as potential anticancer and nonsteroidal anti-inflammatory (NSAIDS) therapeutics or agents (Hussain et al., 2019), and use as antitumor (Lumme et al., 1984) and anticancer agents (Marzano et al., 2009) targeting topoisomerases I and II (Molinaro et al., 2020). Transition metal (e.g., copper) complexes of 1,10-phenanthroline (PHEN) and its derivatives have been widely used in the treatment of a variety of cancers, such as testicular, breast cancers and brain metastasis following lung cancer (Marzano et al., 2002). Because PHEN with its planar structure is an important metal chelator, and various metal complexes containing PHEN and Schiff bases with a functional group with a C=N bond, e.g., the taurine Schiff base copper complex—potently inhibits the activity of the proteasome and induces apoptosis in MDA-MB-231 human breast cancer (Zhang et al., 2008; Hindo et al., 2009; Zhang et al., 2012; Zuo et al., 2013; Zhang et al., 2015), posses anticancer activity (Zhang et al., 2017). Copper complexes of these ligands are of great interest since they exhibit numerous biological activities, such as antitumor (Ranford et al., 1993), antimycobacterial (Saha et al., 2004), antimicrobial (Zoroddu et al., 1996), and intercalating agents of DNA (Erkkila et al., 1999). Because copper is a highly essential mineral nutrient, that is, both beneficial and toxic to the cell due to its redox properties [the two physiologically relevant oxidation states, Cu(II) and Cu(I)—the predominant form in the reducing environment of the cell cytosol]. This precious metal is also increasingly implicated in death pathways and cell proliferation, that is, a fundamental process for the exponential growth of tissue and the development and homeostasis of multicellular organisms. Therefore, copper is a required cofactor for enzymes that mediate a host of essential cellular functions, such as mitochondrial respiration, biosynthesis of hormones, pigments, neurotransmitters, and antioxidant defence. However, oxidative stress and cyctotoxicity can also be induced by dysregulation of copper stores (Lippard and Berg, 1994; Solomon et al., 1996; Que et al., 2008; Hanahan and Weinberg, 2011). From the active site cofactor perspective, copper is also a dynamic signalling metal and metalloallosteric regulator (Brady et al., 2014; Chang, 2015). Recent studies have shown that transition metal signaling has forged new links between chemists and biologists, and thus this linkage helps translating the basic sciences of copper into clinical therapies and diagnostics to exploit vulnerabilities of copper-dependent diseases (Denoyer et al., 2015; Li, 2020; Ge et al., 2022). Another obvious role of copper in metastasis is the regulation of angiogenesis, that is, a fundamental process required for metastatic potential, and many clinical and preclinical studies have provided enough evidence regarding copper coordination compounds to use both as single agents and in combination with other treatments (Denoyer et al., 2015). Combining with some other drugs, such anti-cancer agents with their redox activity have been successfully used to treat brain, ovarian, bladder and breast cancer (Macleod, 1952; Butler et al., 1969; Dwyer et al., 1969). On the other hand, Prodigiosin (Ps), known as a natural medicine, is a secondary and natural metabolite produced by various microorganisms. Ps consists of three pyrrole rings which are active in many reactions (Walsh et al., 2006) with its multiple biological functions, including antibacteria, immunosuppression, anti-inflammatory and anticancer activity (Danevčič et al., 2016). In addition, its anticancer function has attracted immense interests because Ps is a dual mTOR inhibitor. The mTOR pathway necessary for cancer development has two essential components: mTORC1 and mTORC2. Of these, mTORC1 is responsible for tumor cell proliferation and survival while mTORC2 mediates cell migration and invasion (Espona-Fiedler et al., 2012). Typical mTOR inhibitors such as Rapamycin can only inhibit mTORC1 while Ps can inhibit both components (mTORC1 and mTORC2). In addition, a recent study (Wang et al., 2016) has shown that in addition to mTOR inhibition, Ps could also block Wnt/β-catenin signaling pathway which contributes to the cancer initiation and progression. These unique inhibition mechanisms of Ps are strongly suggesting that this compound is a novel and potent candidate of next generation anticancer drug. Currently, research efforts are focusing on the synthesis of Ps derivatives which are expected to provide better anticancer activity and lower cytotoxicity. On the other hand, studying the inhibition of HDAC, a family of proteins whose main function is the removal of acetyl groups from lysine residues on histone and non-histone substrates, thereby leaving an acetate group, will be at least as important as mTORs. Because deregulation of Class I HDAC (HDAC1, 2, 3 and 8) activity from HDACs (of the 18 isoforms) has been associated with cancer (de Ruijter et al., 2003; Weichert, 2009; Müller et al., 2013; Shouksmith et al., 2019) and its involvement has been suggested (Senese et al., 2007) to be crucial in controlling mammalian cell proliferation. In particular, the HDAC1 isoform has been associated with various types of cancer and its overexpression in gastric and prostate cancers has been reported (Choi et al., 2001; Halkidou et al., 2004). It has also been shown (Krusche et al., 2005) that HDAC1 inhibits ER-α protein expression and transcriptional activity. Thus, HDAC1 may regulate the progression of breast cancer and associated metastasis, and inhibition of HDAC1 emerges as a promising therapeutic target for cancer therapy (Senese et al., 2007; Uba and Yelekçi, 2017).

Considering breast cancer, molecular, and cellular mechanisms of HDAC and mTOR inhibition, and the abovementioned proved enzyme-inhibitor-breast cancer correlations, the overarching goal of this study is the development of effective anticancer agents to combat with one of the major health problems in the world, i.e., breast cancer brain metastasis. Since this health issue is suffering 10%–20% of breast cancer patients and considered as a serious issue due to its mortality and lack of efficient therapeutic intervention in the treatment of breast cancer brain metastasis, new anticancer drug(s) need to be exploited. In this context, 1,10-phenanthroline and Ps derivatives including their copper(I) complexes have been selected as target drug candidates owing to their anticancer activities stated in different literatures. Including the supporting experimental evidence (enzymatic assays at either biochemical or cellular levels) for the mechanism of action of inhibitors acted against mTOR and HDAC from those abovementioned relevant literature studies, both mTORs (mTORC1 and mTORC2) and HDACs (HDAC1, 2, 3, and 8—particularly HDAC1) have been aimed on purpose for computational calculations and modeling studies with the selected compounds and their relevant metal complexes. Upon synthesis, purification and characterization of such derivatives and their copper(I) complexes, they have been tested on five breast cancer cell lines MDA-MB-231 (231), MDA-MB-361 (361), HTB131, HTB22, and MDA-MB-231BR (231BR) and one brain cancer cell line (CRL-1620 (CRL)) for the novel anticancer agent screening (and involving one additional normal (non-cancer) cell line MCF10A—a breast epithelial cell collected from patient with fibrocystic disease).

## Materials and methods

Some experimental details have been presented below. All other materials, methods, additional figures and tables, and instrumentation have been described in the Supplementary Material.

### Molecular modeling studies

#### Enzyme preparation

The crystal structure of mTOR and HDAC1 were retrieved from protein data bank and used for protein setup. [http://www.rcsb.org, (for mTOR pdb code: 4jsv; resolution 3.5 Å) and (for HDAC1, pdb code: 4BKX; resolution 3.0 Å)] (Morris et al., 1998). Each structure was cleaned of all water molecules and inhibitors as well as all non-interacting ions before being used in the docking studies. For mTOR and HDAC1, one of the two subunits was taken as the target structure. Using a fast Dreiding-like force field, each protein’s geometry was first optimized and then submitted to the “Clean Geometry” toolkit of Discovery Studio (BIOVIA, 2016) for a more complete check. Missing hydrogen atoms were added based on the protonation state of the titratable residues at a pH of 7.4. Ionic strength was set to 0.145 and the dielectric constant was set to 10. The AutoDock Tools (vv. 1.5.7) (ADT) (Morris et al., 2009) graphical user interface program was employed to setup the enzymes for docking.

#### Ligand setup

The 3D structures of ligand molecules were built, in SPARTAN20 (https://www.wavefun.com/) and optimized at (PM3) level and saved in pdb format. The ADT package was also used here to generate the docking input files of ligands. Autodock4.2.6 (Morris et al., 1998) docking program was used for all docking processes. The detailed procedure for docking methods was reported in our earlier work (Akdoğan et al., 2011).

### Chemistry

#### Materials and methods

Anhydrous dichloromethane and acetonitrile were distilled over CaH_2_ under nitrogen and stored over molecular sieves prior to use. Ligands (**L** ^**2**^ and **L** ^**3**^) (Dietrich-Buchecker and Sauvage, 1990) and ligands (**L** ^**4**^ and **L** ^**5**^) (Zhong et al., 2010; Kang et al., 2014) were prepared using known literature procedures. [Cu(CH_3_CN)_4_]PF_6_ was purchased from Aldrich. All other starting materials were either purchased from commercial sources and used without any further purification, or they were prepared according to the procedures reported in literature. ^1^H NMR and ^13^C NMR (decoupled mode) spectra were recorded on a JEOL ECS-400 or Varian Unity Inova 500 MHz spectrometer. DMSO-*d*_*6*_ (99.9% D with 0.05% v/v TMS) was used as the NMR sample solvent.

#### Preparation of ligands and complexes

The new anticancer drug candidates were synthesized by the routine organic approaches. Precursors of synthetic compounds can be purchased from commercial companies. All synthetic steps are well-established organic reactions. Chemical purification and identification through MS, NMR, and X-ray are the classic methods used for many years. Novel Prodigiosin (**Ps**) and 1,10-phenanthroline derivatives are synthesized and tested. The syntheses of the ligands (**L** ^**2**^–**L** ^**5**^) have been previously reported (Dietrich-Buchecker and Sauvage, 1990; Zhong et al., 2010). The detailed synthetic procedures for the complexes and the molecular structural characterization data for the target compounds are presented in the Supplementary Material (Sections A–C and F).

#### Crystallography

Diffraction data (Supplementary Tables S5–S10 in the Section F of the Supplementary Material) for the 2-trifluoromethansulfonyloxy-4-methoxy-5-[(5-ethyl-2H-pyrrol-2-ylidene)methyl]-1H-pyrrole (Key Intermediate) structure was obtained on a Bruker Kappa APEX-II CCD diffractometer [operated at 1500 W (50 kV, 30 mA) to generate (graphite monochromated) Mo Kα radiation (λ = 0.71073 Å)]. A suitable crystal was selected (a Zeiss Stemi 305 microscope was used to identify a suitable specimen) and the crystal was mounted on a MiTeGen holder in Paratone oil. The crystal was kept at 100 K during data collection. Using Olex2 (Dolomanov et al., 2009), the structure was solved with the XT (Sheldrick, 2015) structure solution program using Intrinsic Phasing and refined with the XL (Sheldrick, 2007) refinement package using Least Squares minimization.

### Biology

Cell lines MDA-MB-231 (231), MDA-MB-361 (361), CRL-1620 (CRL), HTB131, and HTB22 were purchased from American Type Culture Collection (ATCC, Manassas, VA), and MDA-MB-231BR (231BR) was a generous gift from Dr. Paul Lockman (West Virginia University, School of Pharmacy, Morgantown, WV). Cell media, penicillin-streptomycin solution, fetal bovine serum, and phosphate-buffered saline (PBS) were purchased from ATCC. Trypsin-EDTA solution (Corning), cell culture flasks, and pipettes were purchased from VWR.

#### Cell culturing and cytotoxicity tests

MDA-MB-231 (231) and HTB131 were grown with RPIM-1640 medium (with 10% Fetal Bovine Serum and 2% Penicillin-Streptomycin Solution). MDA-MB-231BR (231BR), CRL-1620 (CRL), and MCF10A were grown with DMEM (with 10% Fetal Bovine Serum and 2% Penicillin-Streptomycin Solution). HTB22 was grown with EMEM (with 10% Fetal Bovine Serum and 2% Penicillin-Streptomycin Solution). Cell lines were taken out from liquid nitrogen and thawed in a 37°C water bath. After thawing, cell cultures were transferred to 75 cm^2^ flasks and incubated at 37°C with 5% CO_2_ for 4–7 days. After reaching 80% of cell confluence, cells were ready for subculturing or testing.

Anticancer activity tests were performed using a Cell Counting Kit-8 (CCK8, SigmaAldrich) following manufacturer’s instruction (Shen et al., 2016). Briefly, 100 µl of diluted cell suspension (approximately 10000 cells) was transferred to 96-well plate, incubated at 37°C with 5% CO_2_ for 24 h. Then, the compounds were diluted into a serial of concentrations in DMSO. Next, 0.5 µl of diluted compounds were added to 100 µl of cell medium and gently mixed, resulting in the final concentration of DMSO at 0.5%. 100 µl of the compound contained medium were then used to replace the original medium and incubated at 37°C for 48 h. After incubation with compounds, 10 µl of CCK8 reagent was added to each well and mixed by gently tapping the plate. Then, the plate was incubated at 37°C for 2–4 h until the orange color was dark enough to measure. The plate was measured using a Multiskan microplate reader (Fisher Scientific) at 450 nm. The IC_50_ was calculated by SPSS statistics (IBM). Three controls were used to estimate the analysis: 1) positive control, contained cells and medium (0.5%DMSO) without compounds, used to calculated IC_50_; 2) negative control, contained medium without compounds and cells, used to estimate the influence of medium color to the CCK8 results; 3) chemical control, contained medium and compounds without cells, used to estimate the influence of chemical color to the CCK8 results. All cytotoxicity tests were performed in six biological replicates. Each compound was performed two cycles of screening: primary screening using 10 times serial dilution to determine the approximate concentration and then secondary screening using a narrow concentration range to acquire the accurate IC_50_.

## Results and discussion

### Molecular design, synthesis, and structural characterization

**SCHEME 1:**
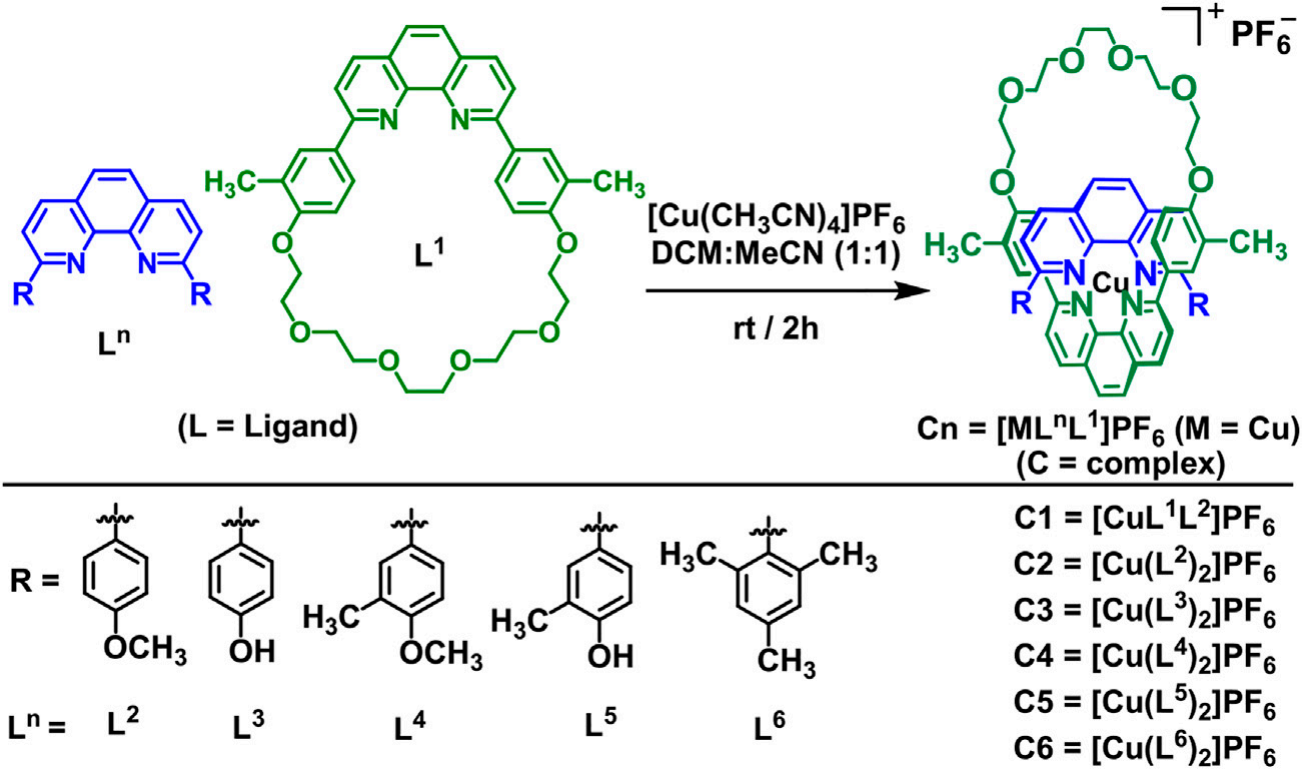
General schematic diagram of the synthesis of the bis(homo- and/or hetero-) copper(I) complexes (**C1–C6**) ‒‒ 2:1 ligand-to-metal complexes, as PF_6_^−^ salts ‒‒ from their respective 1,10-phenanthroline-based ligand derivatives (**L** ^**1**^ **–L** ^**6**^).

The ligands (**L** ^**1**^–**L** ^**5**^) and complexes (**C1**–**C5**) were synthesized (Scheme 1) in high yield by adapting the exact and/or slightly modified literature procedures (our earlier publications) (Kang et al., 2008; Cetin, 2017; Cetin et al., 2017; Cetin et al., 2020). The synthesis of ligand **L** ^**1**^ involves in syntheses (Kang et al., 2008; Kang et al., 2014; Cetin, 2017) of both **L** ^**4**^ and **L** ^**5**^ prior to the final step for the desired product. The methyl groups directly bonded to the phenyl rings in the ligand **L** ^**1**^ (and **L** ^**4**^ as well) helped serve as additional spectroscopic markers for identification of the ligand as well as the corresponding copper(I) complexes **C1** and **C4** that were synthesized in high yields as shown in Scheme 1 (Kang et al., 2008; Kang et al., 2014; Cetin, 2017), and characterized by a variety of spectroscopic techniques. The **C1** is a copper(I) complex of 2,9-di (4-methoxyphenyl)-1,10-phenanthroline (**L** ^**2**^) and the green macrocycle (**L** ^**1**^ in Scheme 1) with the counterion PF_6_^−^ anion as the only bis(hetero-) complex. The synthesis of 2,9-dimesityl-1,10-phenanthroline (dmesp) ligand (**L** ^**6**^) was adapted from the literature procedures (Schmittel et al., 1997; Kohler et al., 2016; Cetin et al., 2017; Kohler et al., 2017; Hayes D et al., 2018; Hayes L et al., 2018). The dmesp ligand was readily prepared by the Suzuki coupling of 2,4,6-trimethyl-phenylboronic acid and 2,9-dichloro-1,10-phenanthroline in good yield.

The syntheses of 2:1 ligand-to-metal complexes (**C1**–**C6**), as PF_6_^−^ salts, were accomplished by addition of tetrakis (acetonitrile)copper(I) hexafluorophosphate to the solutions of ligands in a dry solvent mixture of acetonitrile:dichloromethane (50:50). The adopted synthetic procedures (Schmittel et al., 1997; Kohler et al., 2016; Cetin et al., 2017; Kohler et al., 2017; Hayes D et al., 2018; Hayes L et al., 2018) for complexes are summarized below (Scheme 1). The ligands and complexes were purified and characterized by spectroscopic analysis and the spectra matched that reported in the literature (Schmittel et al., 1997; Kang et al., 2008; Kang et al., 2014; Kohler et al., 2016; Cetin, 2017; Cetin et al., 2017; Kohler et al., 2017; Hayes D et al., 2018; Hayes L et al., 2018; Cetin et al., 2020).

**SCHEME 2:**
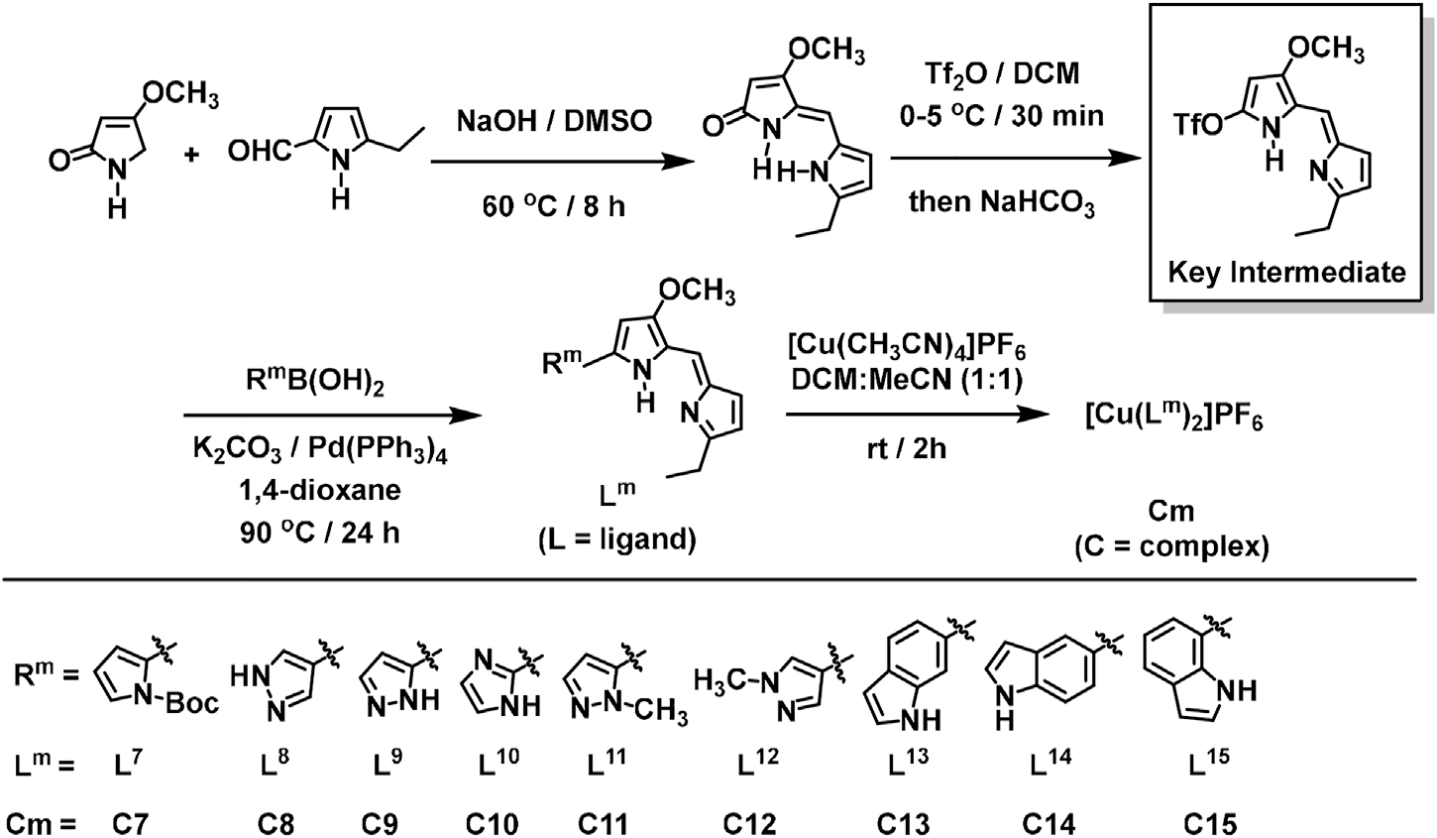
Synthesis of the key intermediate, and general schematic diagram of the synthesis of the Prodigiosin (**Ps**) derivatives (**L** ^**7**^ **–L** ^**15**^) and their respective 2:1 ligand-to-metal copper(I) complexes (**C7–C15**), as PF_6_^−^ salts.

The ligands (**L** ^**7**^–**L** ^**15**^) and complexes (**C7**–**C15**) were synthesized (Scheme 2) in high yield by use of the modified literature procedures (Melvin et al., 2002). The key intermediate and prior precursors shown in Scheme 2 were synthesized and characterized by spectroscopic analysis (Sections B, C, and F in the Supplementary Material) and the spectra matched that reported in the literature (Melvin et al., 2002). Upon synthesis of the key intermediate, the ligands (**L** ^**7**^–**L** ^**15**^) were synthesized by using different commercially available boronic acid precursors and characterized by spectroscopic analysis. Similar to the abovementioned complex synthesis (Kang et al., 2008; Kang et al., 2014; Cetin, 2017), the 2:1 ligand-to-metal complexes (**C7**–**C15**), as PF_6_^−^ salts, were prepared, purified and characterized by spectroscopic analysis (Scheme 2). All the ligands (**L** ^**1**^–**L** ^**15**^) and their corresponding complexes (**C1**–**C15**) are air-stable and capable of being dissolved in most common organic solvents.

### Biology and computational modeling

**FIGURE 1:**
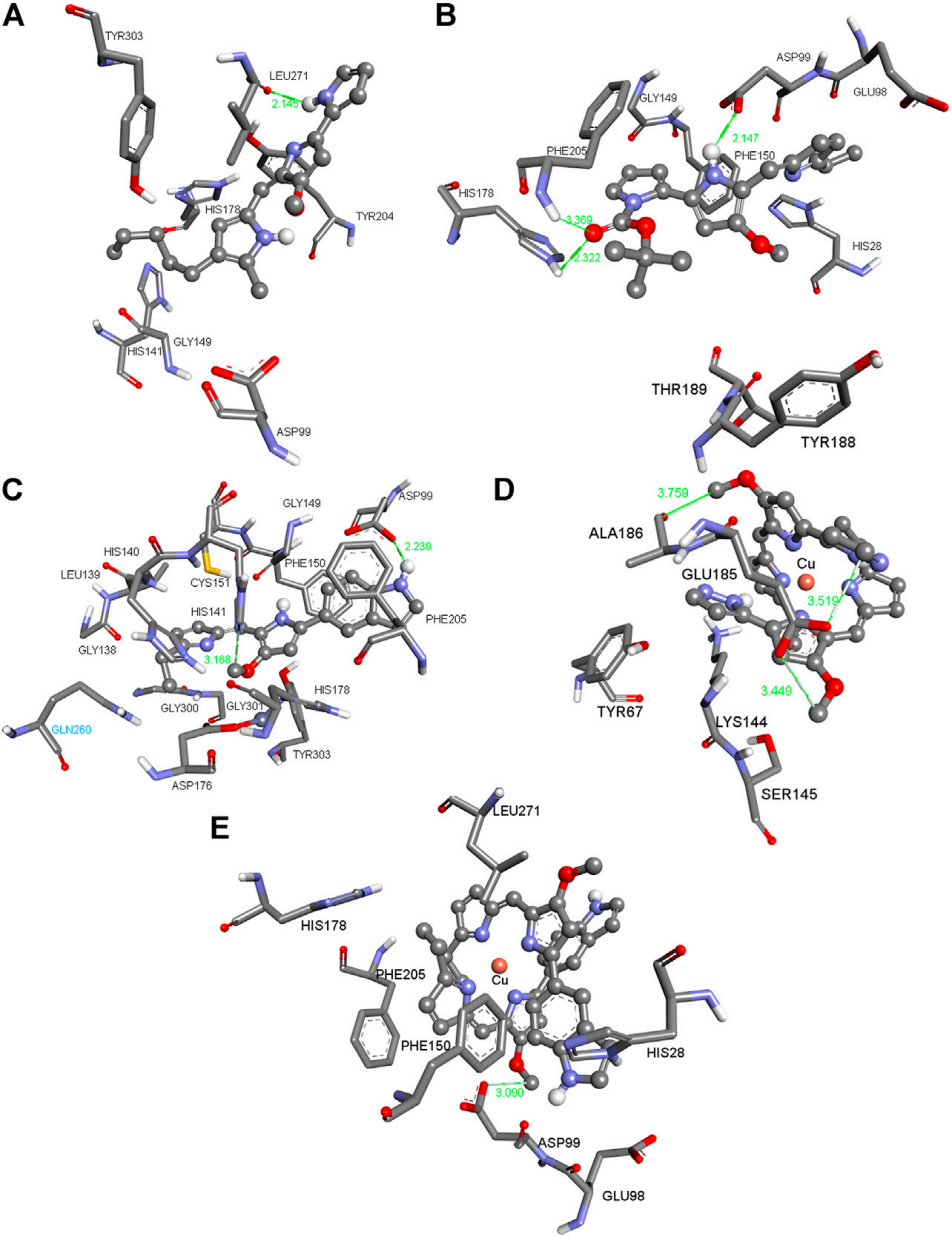
Three-dimensional (3D) images with the displayed hydrogen bonding distances (in green) generated *via* molecular docking of **(A) Ps**, ligands **(B) L** ^**7**^ and **(C) L** ^**14**^ and copper (I) complexes **(D) C9** and **(E) C14** into HDAC1 enzyme. Amino acid side chains are shown as sticks, the ligands as ball and sticks, and the chelating atom coppers are depicted as round copper tone.

**FIGURE 2:**
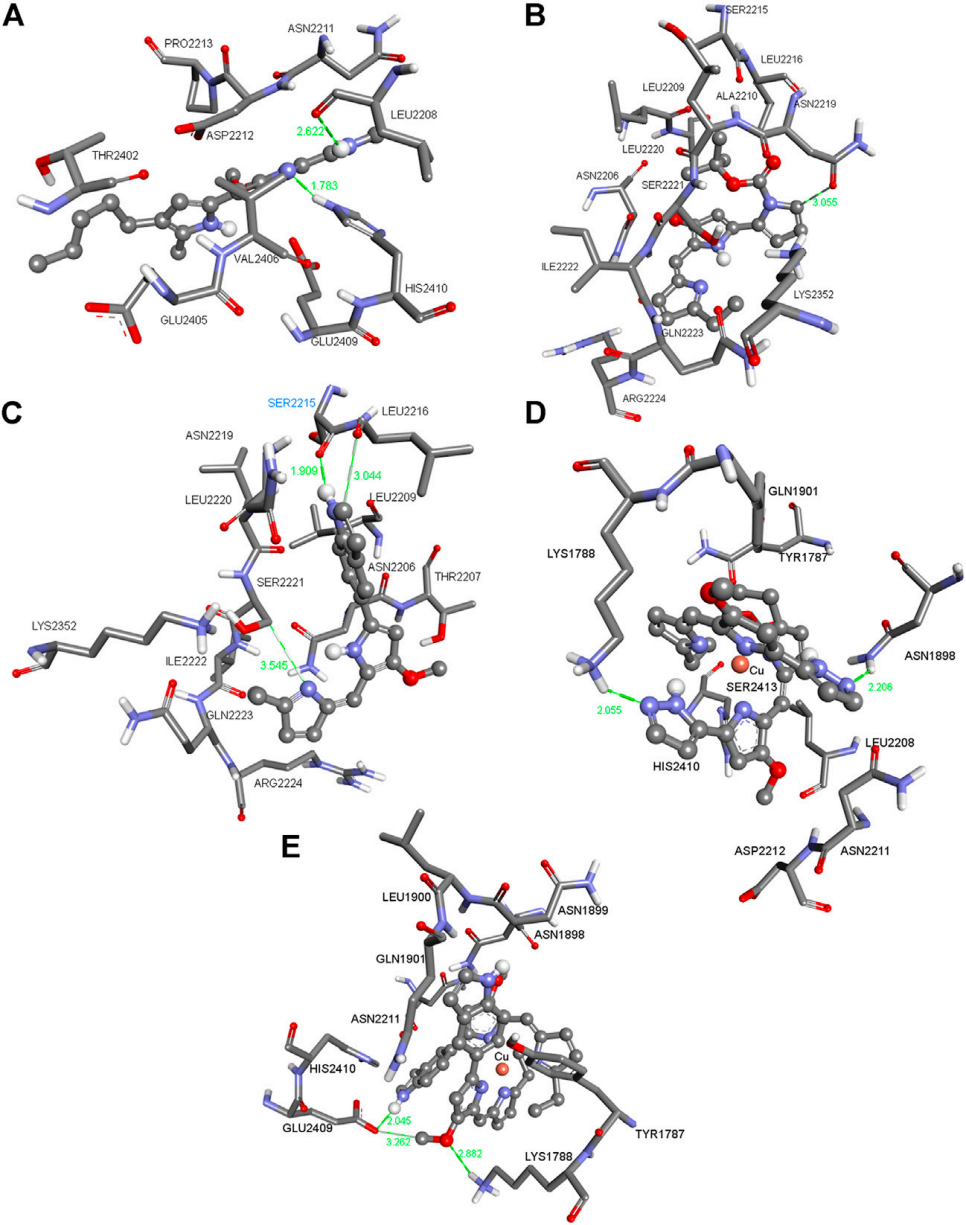
Three-dimensional (3D) images with the displayed hydrogen bonding distances (in green) generated *via* molecular docking of **(A) Ps**, **(B)** ligands **L** ^**7**^ **(C)** and **L** ^**14**^ and copper(I) complexes **(D) C9** and **(E) C14** into mTOR enzyme. Amino acid side chains are shown as sticks, the ligands as ball and sticks, and the chelating atom coppers are depicted as round copper tone.

Both mTOR and HDAC1 enzymes, which are implicated in the biological activities of cancerous cells, were specifically chosen *in silico* works. The level of these enzymes drastically increases in the cancerous state of the cells in comparison to normal cells (Millard et al., 2013; Yang et al., 2013). In order to obtain additional validations to support the experimental results, molecular modeling studies (Table 1, Supplementary Tables S1, S2, and Figures 1, 2) were carried out using newly synthesized compounds against mTOR and HDAC1 enzymes targets.

**TABLE 1:** Calculated binding energies (in kcal/mol) and inhibition constants (in μM) for **Ps**, some ligands (**L** ^**7**^, **L** ^**9**^, and **L** ^**14**^) and copper(I) complexes (**C1**, **C9**, and **C14**). The binding energies and inhibition constants of the other ligands and complexes can be found in the Supplementary Material (Supplementary Table S1).

Compound ID	mTOR		HDAC1	
	Binding energies (kcal/mol)	Inhibition constants (μM)	Binding energies (kcal/mol)	Inhibition constants (μM)
**Ps**	−4.89	258.68	−6.99	7.53
**L** ^7^	−5.04	202.51	−7.06	6.64
**L** ^9^	−4.84	281.77	−6.35	22.27
**L** ^14^	−6.62	14.15	9.08	0.220
**C1**	−8.31	0.808	−6.88	9.10
**C9**	−5.84	52.19	−4.16	885.39
**C14**	−5.90	47.11	−6.69	12.52

The compounds in Figures 1, 2 (for the other compounds, see Supplementary Tables S1, S2 in the Supplementary Material) were docked into the active sites of mTOR and HDAC1 enzymes to predict their binding affinities and binding modes employing AutoDock 4.2 docking software. Although the experimental studies were carried out using whole cells *in silico* studies on these crucial enzymes generated consistent results agreeing with the experimental inhibition values. All the compounds were more active than that of reference compound **Ps** (405 μM for mTOR and 348 μm for HDAC1). Compounds **C1** and **L** ^**14**^ showed excellent binding affinity against mTOR (0.808 μM) and HDAC1 (0.220 μM) enzymes, supporting the experimental data (Table 1).

Figure 1A shows the pose of the **Ps** in the binding pocket of HDAC1 enzyme. A strong hydrogen bond occurs between the amide group of the ILE271 and the N–H bond of the **Ps**. The other interacting side chain residues with **Ps** are TYR303, TYR204, HIS178, GLY149, HIS141 and ASP99 which make various van der Waals and hydrophobic interactions with **Ps**. Figure 1B shows the pose of **L** ^**7**^ in the binding pocket of HDAC1 enzyme. Three strong hydrogen bonds occur between the ASP99, PHE205, and HIS178 and the ligand **L** ^**7**^ and such strong interactions make this compound one of the best inhibitors among this series. Figure 1C indicates the pose of L14 in the binding pocket of the HDAC1 enzyme. With varying distances, three significant hydrogen bonds occur between the SER2221, LEU2216, and SER2215. Figures 1D,E show the copper complexes **C9** and **C14**. These complexes interact with the nearby amino acids and make strong hydrogen bonds with the amino acids lining the binding pockets. These strong hydrogen bonds and the other hydrophobic and van der Waals interactions make these **C9** and **C14** complexes potential inhibitors against HDAC1 enzyme. Similarly, Figures 2A–E show the pose of **Ps**, **L** ^**7**^, **L** ^**14**^, **C9**, and **C14** in the binding pocket of mTOR enzyme, respectively.

**FIGURE 3:**
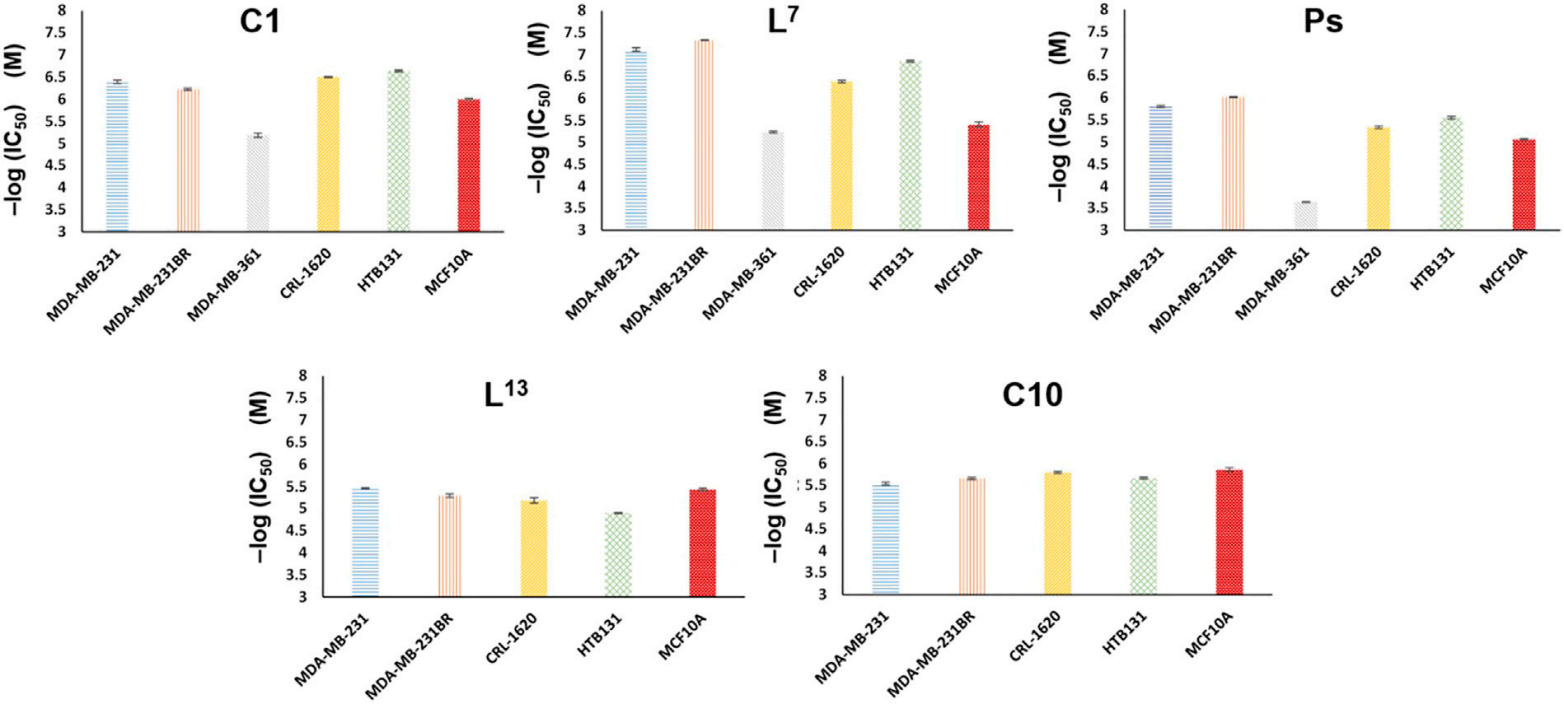
Anticancer activities of compounds **C1**, **L** ^**7**^, **Ps**, **L** ^**13**^, and **C10**. A negative log (IC_50_) value is used to represent the anticancer activity. The lower the IC_50_, the higher the activity is. Both compounds **C1** and **L** ^**7**^show higher anticancer activity than native drug, **Ps**.

**FIGURE 4:**
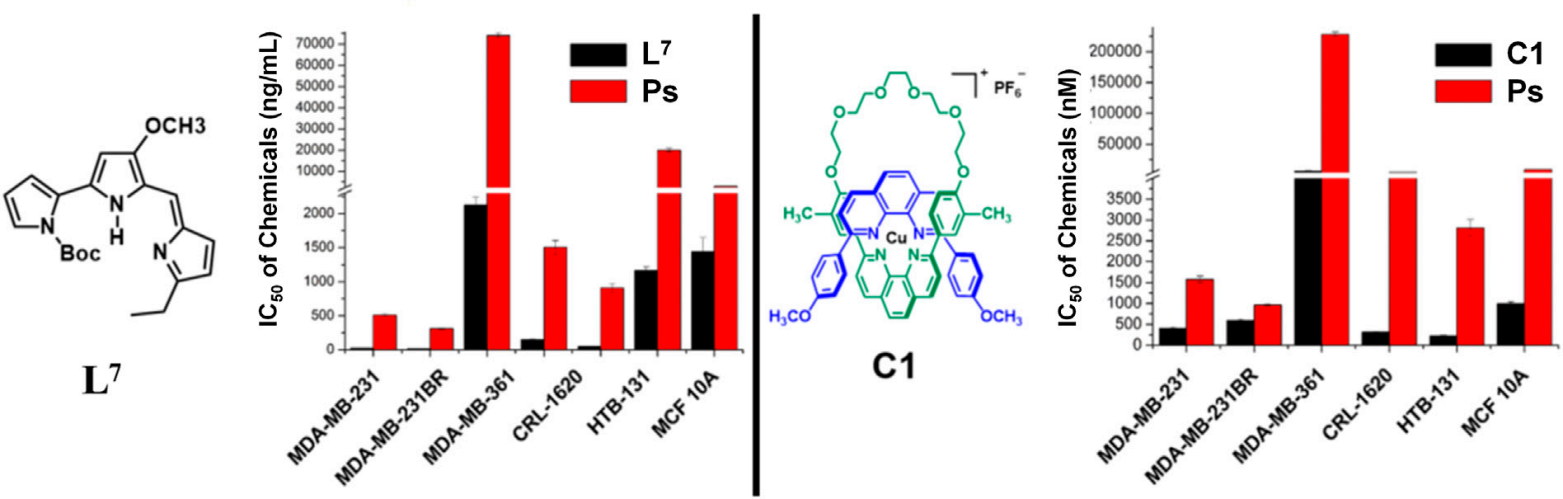
Anticancer activities of compounds **L** ^**7**^ and **C1**. The lower the IC_50_, the higher the activity is. Both compounds show extremely higher anticancer activity than native drug, **Ps**.

The newly synthesized molecules in which their binding affinities were predicted *in silico* were tested on five different breast cancer lines, MDA-MB-231 (231), MDA-MB-361 (361), HTB131, HTB22, and MDA-MB-231BR (231BR), and one brain cancer cell line, CRL-1620 (CRL). The cell lines have been treated with these synthetic molecules to estimate their IC_50_ anticancer capacities (Section E in the Supplementary Material). Among these cell lines, 231BR attracted most of our interest because it is a brain metastatic specific breast cancer cell line that has 100% rate of brain metastasis and cannot migrate to any other organs (Yoneda et al., 2001). The anticancer capacities of each compound were first screened by 10 times dilution to generate different molar concentrations ranging in 5 orders of magnitudes. This wide concentration range was applied for primary screening (Supplementary Table S3) to narrow down the targets. The approximate IC_50_ was estimated in the primary screening step and used to design the concentration range of the secondary cytotoxicity tests (Supplementary Table S4). Any compounds showed IC_50_ less than 10^−5^ M in primary screening were selected for the secondary cytotoxicity tests that had a molar concentration range within two orders of magnitude to acquire the accurate IC_50_. Anticancer drug taxol (paclitaxel) was used as the reference to estimate the efficacy of each compound. Among these cell lines, 231BR was used in the primary screening because this cell grows the fastest and most sensitive to all compounds. The anticancer activity against 231BR is of highest interest. Meanwhile, the same treatments were performed against the normal breast cell line, MCF10A, to test their cytotoxicity. After the primary screening, five compounds, **L** ^**7**^, **C1**, **Ps**, **L** ^**13**^, and **C10** exhibited relatively promising anticancer activities against 231BR with an IC_50_ < 10^−5^ M (Supplementary Table S3). Then, a secondary cytotoxicity test was performed on each of these five compounds using a narrower concentration range (at least three biological replicates) to determine their IC_50_ against different cancer cell lines (Supplementary Table S4). Figure 3 depicts the anticancer activities of these compounds. A negative logarithm of IC_50_ was applied to represent the corresponding anticancer activity. Two of the abovementioned compounds (**C1** and **L** ^**7**^) have demonstrated high anticancer activities and relatively low (Figure 4) cytotoxicity (on normal cell line MCF10A). A prodigiosin (**Ps**) derivative **L** ^**7**^ exhibited lowest IC_50_ (highest activities) against brain-seeking cell line 231BR (4.63E-08 ± 6.50E-10 M) and 231 (7.58E-08 ± 7.20E-09 M) that are considered as most invasion breast cancer cell lines while showed more than 80 times lower cytotoxity on normal cell line (3.39E-06 ± 5.57E-07 M). Additionally, **L** ^**7**^ also exhibited an overwhelming anticancer activity when compared with the precursor prodigiosin (**Ps**), the natural drug. The anti-breast cancer activity of the compound **L** ^**7**^ is more than 20 times better than the precursor drug, **Ps**.

Compound **C1,** on the other hand, is a novel compound. Figure 3 depicts that the compound **C1** that we have thus far synthesized has the different selectivity among cancer cell lines from compound **L** ^**7**^. The copper(I) complex, **C1**, has better anticancer activities on breast cancer cell line HTB131 (2.28E-07 ± 1.11E-08 M) and brain cancer cell line CRL (3.15E-07 ± 9.77E-09 M). This new compound may provide us a novel anticancer mechanism and target, with some possible structural modifications and changes to decrease cytotoxicity on normal cells. However, all compounds showed lower anticancer activity against 361, demonstrating the selectivities need to be considered cautiously. Moreover, as a reference, commercialized anticancer reagent Taxol (paclitaxel) achieved more than 700 times lower cytotoxicity on normal cell line (IC_50_ of 3.00E-05 M) relative to 231BR (IC_50_ 3.90E-08 M), making it an efficient anticancer drug. When compared to commercialized anticancer reagent paclitaxel, the relatively lower anticancer activity on cancer cell lines and higher cytotoxicity on normal cell line of all compounds demand future efforts to enhance the anticancer activity while decreasing the cytotoxicity.

**FIGURE 5:**
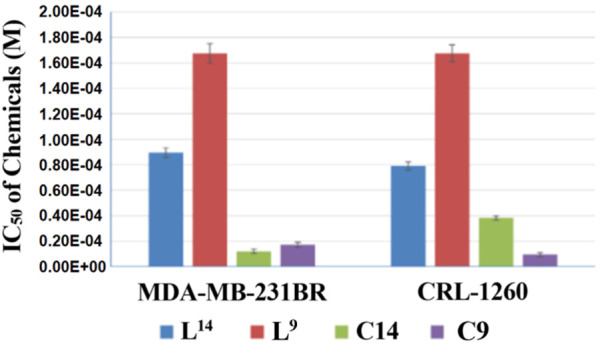
IC_50_ of compounds **L** ^**14**^, **L** ^**9**^, **C14**, and **C9**. The copper(I) complexes significantly increase the anticancer activity compared with their relevant ligands.

**FIGURE 6:**
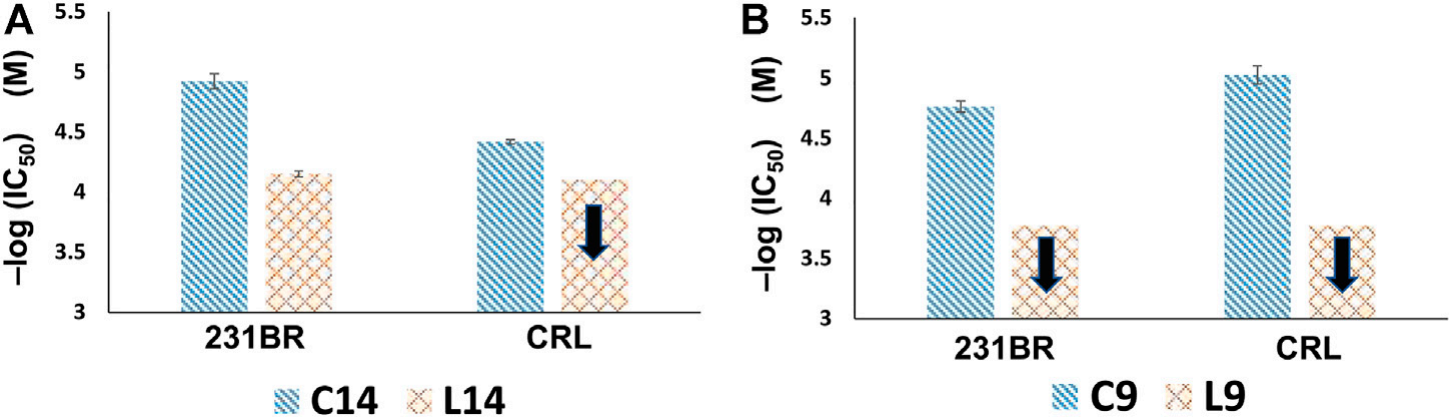
Anticancer activity comparisons of **(A) C14** and **L** ^**14**^, and **(B) C9** and **L** ^**9**^. The black arrow denotes that the anticancer activity is lower than the corresponding value because the concentration in cytotoxicity test could not set higher due to the solubility issue. The copper(I) complexes significantly increase the anticancer activity compared with their relevant ligands.

In addition, copper(I) complexes of the ligands were tested on the same cell lines as well. Figures 5, 6 showed the results that when coupled with copper(I), the anticancer activity increased. Compounds **C14** and **C9** are copper(I) complex of compounds **L** ^**14**^ and **L** ^**9**^, respectively. IC_50_ of complexes **C14** and **C9** significantly decreased which meant the increasing of anticancer activity when compared with their ligands **L** ^**14**^ and **L** ^**9**^. When compared **C14** and **L** ^**14**^, the anticancer activity increased 5.9 times on 231BR from IC_50_ 7.08E-05 ± 3.89E-06 M to 1.20E-05 ± 1.75E-06 M (Figure 6A). The copper effect was even more significant for **C9**. When compared **C9** and **L** ^**9**^, the anticancer activity increased more than 9.8 times on CRL from IC_50_ > 1.68E-04 M to 1.72E-05 ± 1.86E-06 M (Figure 6B). Although the ligands showed less activity than ligand **L** ^**7**^, this result provided a possible way for further improvement of the anticancer activity.

Among all the breast cancer cell lines, MDA-MB-231BR (231BR) is unique. This cell line has a 100% tendency to brain metastasis and cannot migrate to any other organs (Yoneda et al., 2001; Gupta et al., 2013). This brain-seeker cell line can be substantial in the breast cancer brain metastasis studies. We have reported the differential gene expressions in 231BR compared with other cell lines (Peng et al., 2019). Through anticancer agent screening, we can acquire compounds which have extremely high activity on this cell line compared to others. This may provide a potential treatment specific for the breast cancer patients who are at a large brain metastasis risk.

## Conclusion

In this study, a series of 1,10-phenanthroline and Prodigiosin (Ps) derivatives consisting of their 2:1 ligand-to-metal copper(I) complexes, as PF_6_^−^ salts, have been isolated and characterized. Their biological activities were tested *in vitro* on six different cell lines, and several compounds with good activity were preliminarily verified. Of these compounds, **L** ^**7**^, **C1**, **Ps**, **L** ^**13**^, and **C10** exhibited relatively promising anticancer activities against our highest interest cell line, 231BR, with an IC_50_ < 10^−5^ M after the primary screenings. **C1** and **L** ^**7**^ have also demonstrated higher anticancer activities and relatively lower cytotoxicities on normal cell line MCF10A in the performance of the secondary cytotoxicity test using a narrower concentration range against different cancer cell lines. **L** ^**7**^, in particular, has exhibited lowest IC_50_ (highest activity) against brain-seeking cell line 231BR and 231 that are considered as most invasion breast cancer cell lines while also showing more than 80 times lower cytotoxity on MCF10A. In addition, **L** ^**7**^ has an overwhelming anticancer activity (more than 20 times better) than **Ps**, the natural drug. **C1**, on the other hand, has better anticancer activities on HTB131 and CRL compared to **L** ^**7**^. The IC_50_ of **C14** and **C9** also showed increased anticancer activities compared to their relevant ligands **L** ^**14**^ and **L** ^**9**^, respectively. When compared **C14** and **L** ^**14**^, the anticancer activity of **C14** increased by 5.9 times on 231BR, whilst the anticancer activity of another copper(I) complex **C9** on CRL also rised by 9.8 times. These increases showed that the copper(I) complexes of the ligands were even more significant role on anticancer activities of such complexes. To obtain additional validations of the experimental data, some *in silico* modeling studies were carried out with mTOR and HDAC1 enzymes to predict their binding affinities and binding modes. Such studies also supported the experimental data that **C1** and **L** ^**14**^ showed excellent binding affinity against mTOR and HDAC1 enzymes. Through anticancer agent screening, we can acquire the compounds which have very high activity on 231BR compared to other cell lines and may provide great therapeutical potential for the breast cancer patients who are at a large brain metastasis risk. Further syntheses of some of the above listed compounds as well as the structural optimization and modification of the synthesized molecules are still under study in our laboratories.

## Data Availability

The original contributions presented in the study are included in the article/Supplementary Material, further inquiries can be directed to the corresponding and co-corresponding authors.

## References

[B1] Akdoğan E. D. Erman B. Yelekçi K. (2011). *In silico* design of novel and highly selective lysine-specific histone demethylase inhibitors. Turk. J. Chem. 35, 523–542. 10.3906/kim-1102-985

[B2] Bian X. Liang Z. Feng A. Salgado E. Shim H. (2018). HDAC inhibitor suppresses proliferation and invasion of breast cancer cells through regulation of miR-200c targeting CRKL. Biochem. Pharmacol. 147, 30–37. 10.1016/j.bcp.2017.11.008 29155146 PMC5733635

[B87] BIOVIA (2016). Dassault Systèmes, Discovery Studio 2016. San Diego: Dassault Systèmes.

[B3] Brady D. C. Crowe M. S. Turski M. L. Hobbs G. A. Yao X. Chaikuad A. (2014). Copper is required for oncogenic BRAF signalling and tumorigenesis. Nature 509 (7501), 492–496. 10.1038/nature13180 24717435 PMC4138975

[B4] Butler H. M. Hurse A. Thursky E. Shulman A. (1969). Bactericidal action of selected phenanthroline chelates and related compounds. Aust. J. Exp. Biol. Med. Sci. 47 (5), 541–552. 10.1038/icb.1969.148 4313771

[B5] Cetin M. M. Hodson R. T. Hart C. R. Cordes D. B. Findlater M. Casadonte D. J. Jr. (2017). Characterization and photocatalytic behavior of 2, 9-di(aryl)-1, 10-phenanthroline copper (I) complexes. Dalton Trans. 46 (20), 6553–6569. 10.1039/c7dt00400a 28463361

[B6] Cetin M. M. Shafiei-Haghighi S. Chen J. Zhang S. Miller A. C. Unruh D. K. (2020). Synthesis, structures, photophysical properties, and catalytic characteristics of 2, 9-dimesityl-1, 10-phenanthroline (dmesp) transition metal complexes. J. Polym. Sci. (2020). 58 (8), 1130–1143. 10.1002/pol.20190276

[B7] Cetin M. M. (2017). Syntheses and characterization of copper(I) complexes for study of dynamic supramolecular ring-chain equilibria and application as photoredox catalysts, Lubbock, TX, USA: Texas Tech University. PhD Dissertation.

[B8] Chang C. J. (2015). Searching for harmony in transition-metal signaling. Nat. Chem. Biol. 11 (10), 744–747. 10.1038/nchembio.1913 26379012

[B9] Choi J.-H. Kwon H. J. Yoon B.-I. Kim J.-H. Han S. U. Joo H. J. (2001). Expression profile of histone deacetylase 1 in gastric cancer tissues. Jpn. J. Cancer Res. 92 (12), 1300–1304. 10.1111/j.1349-7006.2001.tb02153.x 11749695 PMC5926683

[B10] Danevčič T. Vezjak M. B. Zorec M. Stopar D. (2016). Prodigiosin - a multifaceted *Escherichia coli* antimicrobial agent. PLoS One 11 (9), e0162412. 10.1371/journal.pone.0162412 27612193 PMC5017725

[B11] de Ruijter A. J. M. van Gennip A. H. Caron H. N. Kemp S. van Kuilenburg A. B. P. (2003). Histone deacetylases (HDACs): Characterization of the classical HDAC family. Biochem. J. 370 (3), 737–749. 10.1042/bj20021321 12429021 PMC1223209

[B12] Denoyer D. Masaldan S. La Fontaine S. Cater M. A. (2015). Targeting copper in cancer therapy: ‘*Copper that cancer* . Metallomics 7 (11), 1459–1476. 10.1039/C5MT00149H 26313539

[B13] Dietrich-Buchecker C. Sauvage J.-P. (1990). Templated synthesis of interlocked macrocyclic ligands, the catenands. Preparation and characterization of the prototypical bis-30 membered ring system. Tetrahedron 46 (2), 503–512. 10.1016/s0040-4020(01)85433-8

[B14] Dolomanov O. V. Bourhis L. J. Gildea R. J. Howard J. A. K. Puschmann H. (2009). OLEX2: A complete structure solution, refinement and analysis program. J. Appl. Cryst. 42 (2), 339–341. 10.1107/s0021889808042726

[B15] Dwyer F. P. Reid I. K. Shulman A. Laycock G. M. Dixson S. (1969). The biological actions of 1, 10-phenanthroline and 2, 2'-bipyridine hydrochlorides, quaternary salts and metal chelates and related compounds. 1. Bacteriostatic action on selected gram-positive, gram-negative and acid-fast bacteria. Aust. J. Exp. Biol. Med. Sci. 47 (2), 203–218. 10.1038/icb.1969.21 4307583

[B16] Erkkila K. E. Odom D. T. Barton J. K. (1999). Recognition and reaction of metallointercalators with DNA. Chem. Rev. 99 (9), 2777–2796. 10.1021/cr9804341 11749500

[B17] Espona-Fiedler M. Soto-Cerrato V. Hosseini A. Lizcano J. M. Guallar V. Quesada R. (2012). Identification of dual mTORC1 and mTORC2 inhibitors in melanoma cells: Prodigiosin vs. obatoclax. Biochem. Pharmacol. 83 (4), 489–496. 10.1016/j.bcp.2011.11.027 22155350

[B18] Fasolo A. Sessa C. (2012). Targeting mTOR pathways in human malignancies. Curr. Pharm. Des. 18 (19), 2766–2777. 10.2174/138161212800626210 22475451

[B19] Fricker S. P. (1994). Metal compounds in cancer therapy. London, United Kingdom: Chapman & Hall, 10–15.

[B20] Ge E. J. Bush A. I. Casini A. Cobine P. A. Cross J. R. DeNicola G. M. (2022). Connecting copper and cancer: From transition metal signalling to metalloplasia. Nat. Rev. Cancer 22, 102–113. 10.1038/s41568-021-00417-2 34764459 PMC8810673

[B21] Guo Q. Cheng K. Wang X. Li X. Yu Y. Hua Y. (2020). Expression of HDAC1 and RBBP4 correlate with clinicopathologic characteristics and prognosis in breast cancer. Int. J. Clin. Exp. Pathol. 13 (3), 563–572. Available at: https://pubmed.ncbi.nlm.nih.gov/32269697/. 32269697 PMC7137008

[B22] Gupta P. Adkins C. Lockman P. Srivastava S. K. (2013). Metastasis of breast tumor cells to brain is suppressed by phenethyl isothiocyanate in a novel *in vivo* metastasis model. PLoS One 8 (6), e67278. 10.1371/journal.pone.0067278 23826254 PMC3695065

[B23] Halkidou K. Gaughan L. Cook S. Leung H. Y. Neal D. E. Robson C. N. (2004). Upregulation and nuclear recruitment of HDAC1 in hormone refractory prostate cancer. Prostate 59 (2), 177–189. 10.1002/pros.20022 15042618

[B24] Hanahan D. Weinberg R. A. (2011). Hallmarks of cancer: The next generation. Cell 144 (5), 646–674. 10.1016/j.cell.2011.02.013 21376230

[B25] Hayes D D. Kohler L. Hadt R. G. Zhang X. Liu C. Mulfort K. L. (2018). Excited state electron and energy relays in supramolecular dinuclear complexes revealed by ultrafast optical and X-ray transient absorption spectroscopy. Chem. Sci. 9 (4), 860–875. 10.1039/c7sc04055e 29629153 PMC5873173

[B26] Hayes L D. Kohler L. Chen L. X. Mulfort K. L. (2018). Ligand mediation of vectorial charge transfer in Cu(I)diimine chromophore–acceptor dyads. J. Phys. Chem. Lett. 9 (8), 2070–2076. 10.1021/acs.jpclett.8b00468 29614231

[B27] Heffeter P. Jakupec M. A. Körner W. Wild S. von Keyserlingk N. G. Elbling L. (2006). Anticancer activity of the lanthanum compound [tris(1, 10-phenanthroline)lanthanum(III)]trithiocyanate (KP772; FFC24). Biochem. Pharmacol. 71 (4), 426–440. 10.1016/j.bcp.2005.11.009 16343446

[B28] Hindo S. S. Frezza M. Tomco D. Heeg M. J. Hryhorczuk L. McGarvey B. R. (2009). Metals in anticancer therapy: Copper(II) complexes as inhibitors of the 20S proteasome. Eur. J. Med. Chem. 44 (11), 4353–4361. 10.1016/j.ejmech.2009.05.019 19559507 PMC2759842

[B29] Huang X. Hou Y. Weng X. Pang W. Hou L. Liang Y. (2021). Diethyldithiocarbamate-copper complex (CuET) inhibits colorectal cancer progression *via* miR-16-5p and 15b-5p/ALDH1A3/PKM2 axis-mediated aerobic glycolysis pathway. Oncogenesis, 10, 4. doi: 10.1038/s41389-020-00295-7 33419984 PMC7794448

[B30] Hussain A. AlAjmi M. F. Rehman T. Amir S. Husain F. M. Alsalme A. (2019). Copper(II) complexes as potential anticancer and nonsteroidal anti-inflammatory agents: *In vitro* and *in vivo* studies. Sci. Rep. 9, 5237. doi: 10.1038/s41598-019-41063-x 30918270 PMC6437194

[B31] Kang S. Berkshire B. M. Xue Z. Gupta M. Layode C. May P. A. (2008). Polypseudorotaxanes *via* ring-opening metathesis polymerizations of [2]Catenanes. J. Am. Chem. Soc. 130 (46), 15246–15247. 10.1021/ja806122r 18939837

[B32] Kang S. Cetin M. M. Jiang R. Clevenger E. S. Mayer M. F. (2014). Synthesis of metalated pseudorotaxane polymers with full control over the average linear density of threaded macrocycles. J. Am. Chem. Soc. 136 (36), 12588–12591. 10.1021/ja507167k 25153841

[B33] Kawai H. Li H. Avraham S. Jiang S. Avraham H. K. (2003). Overexpression of histone deacetylase HDAC1 modulates breast cancer progression by negative regulation of estrogen receptor alpha. Int. J. Cancer 107 (3), 353–358. 10.1002/ijc.11403 14506733

[B34] Kohler L. Hadt R. G. Hayes D. Chen L. X. Mulfort K. L. (2017). Synthesis, structure, and excited state kinetics of heteroleptic Cu(I) complexes with a new sterically demanding phenanthroline ligand. Dalton Trans. 46 (38), 13088–13100. 10.1039/c7dt02476b 28944388

[B35] Kohler L. Hayes D. Hong J. Carter T. J. Shelby M. L. Fransted K. A. (2016). Synthesis, structure, ultrafast kinetics, and light-induced dynamics of CuHETPHEN chromophores. Dalton Trans. 45 (24), 9871–9883. 10.1039/c6dt00324a 26924711

[B36] Kölbl A. C. Andergassen U. Jeschke U. (2015). The role of glycosylation in breast cancer metastasis and cancer control. Front. Oncol. 5, 219. 10.3389/fonc.2015.00219 26528431 PMC4602128

[B37] Krusche C. A. Wülfing P. Kersting C. Vloet A. Böcker W. Kiesel L. (2005). Histone deacetylase-1 and -3 protein expression in human breast cancer: A tissue microarray analysis. Breast Cancer Res. Treat. 90 (1), 15–23. 10.1007/s10549-004-1668-2 15770522

[B38] Leone J. P. Leone B. A. (2015). Breast cancer brain metastases: The last frontier. Exp. Hematol. Oncol. 4, 33. 10.1186/s40164-015-0028-8 26605131 PMC4657380

[B39] Li Y. (2020). Copper homeostasis: Emerging target for cancer treatment. IUBMB Life, 72 (9), 1900–1908. doi: 10.1002/iub.2341 32599675

[B40] Lin S. Q. Kemmner W. Grigull S. Schlag P. M. (2002). Cell surface alpha 2, 6 sialylation affects adhesion of breast carcinoma cells. Exp. Cell Res. 276 (1), 101–110. 10.1006/excr.2002.5521 11978012

[B41] Lippard S. J. Berg J. M. (1994). Principles of bioinorganic chemistry, xvii. Sausalito, California, United States: University Science Books, 411.

[B84] Lu Y. Liu W. (2020). Selective estrogen receptor degraders (SERDs): A promising strategy for estrogen receptor positive endocrine-resistant breast cancer. J. Med. Chem. 63 (24), 15094–15114. 10.1021/acs.jmedchem.0c00913 33138369

[B85] Lu Y. Ma X. Chang X. Liang Z. Lv L. Shan M. (2022). Recent development of gold(I) and gold(III) complexes as therapeutic agents for cancer diseases. Chem. Soc. Rev. 51 (13), 5518–5556. 10.1039/D1CS00933H 35699475

[B42] Lumme P. Elo H. Jänne J. (1984). Antitumor activity and metal complexes of the first transition series. *Trans*-bis(salicylaldoximato)copper( II) and related copper( II) complexes, a novel group of potential antitumor agents. Inorganica Chim. Acta 92 (4), 241–251. 10.1016/S0020-1693(00)80045-6

[B43] Macleod R. A. (1952). The toxicity of *o*-phenanthroline for lactic acid bacteria. J. Biol. Chem. 197 (2), 751–761. 10.1016/S0021-9258(18)55631-3 12981107

[B44] Marzano C. Pellei M. Tisato D. Santini C. (2009). Copper complexes as anticancer agents. Anticancer. Agents Med. Chem. 9 (2), 185–211. 10.2174/187152009787313837 19199864

[B45] Marzano C. Trevisan A. Giovagnini L. Fregona D. (2002). Synthesis of a new platinum(II) complex: Anticancer activity and nephrotoxicity *in vitro* . Toxicol. Vitro 16 (4), 413–419. 10.1016/s0887-2333(02)00022-x 12110280

[B46] Melvin M. S. Tomlinson J. T. Park G. Day C. S. Saluta G. R. Kucera G. L. (2002). Influence of theA-ring on the proton affinity and anticancer properties of the prodigiosins. Chem. Res. Toxicol. 15 (5), 734–741. 10.1021/tx025507x 12018996

[B47] Millard C. J. Watson P. J. Celardo I. Gordiyenko Y. Cowley S. M. Robinson C. V. (2013). Class I HDACs share a common mechanism of regulation by inositol phosphates. Mol. Cell 51 (1), 57–67. 10.1016/j.molcel.2013.05.020 23791785 PMC3710971

[B48] Min K.-N. Joung K.-E. Kim D.-K. Sheen Y.-Y. (2012). Anti-cancer effect of IN-2001 in MDA-MB-231 human breast cancer. Biomol. Ther. 20 (3), 313–319. 10.4062/biomolther.2012.20.3.313 PMC3794529 24130929

[B49] Molinaro C. Martoriati A. Pelinski L. Cailliau K. (2020). Copper complexes as anticancer agents targeting topoisomerases I and II. Cancers, 12 (10), 2863. doi: 10.3390/cancers12102863 PMC7601307 33027952

[B50] Morris G. M. Goodsell D. S. Halliday R. S. Huey R. Hart W. E. Belew R. K. (1998). Automated docking using a Lamarckian genetic algorithm and an empirical binding free energy function. J. Comput. Chem. 19 (14), 1639–1662. 10.1002/(sici)1096-987x(19981115)19:14<1639::aid-jcc10>3.0.co;2-b

[B51] Morris G. M. Huey R. Lindstrom W. Sanner M. F. Belew R. K. Goodsell D. S. (2009). AutoDock4 and AutoDockTools4: Automated docking with selective receptor flexibility. J. Comput. Chem. 30 (16), 2785–2791. 10.1002/jcc.21256 19399780 PMC2760638

[B52] Müller B. M. Jana L. Kasajima A. Lehmann A. Prinzler J. Budczies J. (2013). Differential expression of histone deacetylases HDAC1, 2 and 3 in human breast cancer - overexpression of HDAC2 and HDAC3 is associated with clinicopathological indicators of disease progression. BMC Cancer 13 (1), 215. 10.1186/1471-2407-13-215 23627572 PMC3646665

[B53] Pangeni R. P. Channathodiyil P. Huen D. S. Eagles L. W. Johal B. K. Pasha D. (2015). The *GALNT9, BNC1* and *CCDC8* genes are frequently epigenetically dysregulated in breast tumours that metastasise to the brain. Clin. Epigenetics 7, 57. 10.1186/s13148-015-0089-x 26052355 PMC4457099

[B54] Peng W. hu R. Zhou S. Mirzaei P. Mechref Y. (2019). Integrated transcriptomics, proteomics, and glycomics reveals the association between up-regulation of sialylated N-glycans/Integrin and breast cancer brain metastasis. Sci. Rep. 9 (1), 17361. 10.1038/s41598-019-53984-8 31758065 PMC6874669

[B55] Que E. L. Domaille D. W. Chang C. J. (2008). Metals in neurobiology: Probing their chemistry and biology with molecular imaging. Chem. Rev. 108 (5), 1517–1549. 10.1021/cr078203u 18426241

[B56] Ranford J. D. Sadler P. J. Tocher D. A. (1993). Cytotoxicity and antiviral activity of transition-metal salicylato complexes and crystal structure of Bis(diisopropylsalicylato)(1, 10-phenanthroline)copper(II). J. Chem. Soc. Dalton Trans. 1993 (22), 3393–3399. 10.1039/dt9930003393

[B57] Rostami R. Mittal S. Rostami P. Tavassoli F. Jabbari B. (2016). Brain metastasis in breast cancer: A comprehensive literature review. J. Neurooncol. 127 (3), 407–414. 10.1007/s11060-016-2075-3 26909695

[B58] Saha D. K. Sandbhor U. Shirisha K. Padhye S. Deobagkar D. Anson C. E. (2004). A novel mixed-ligand antimycobacterial dimeric copper complex of ciprofloxacin and phenanthroline. Bioorg. Med. Chem. Lett. 14 (12), 3027–3032. 10.1016/j.bmcl.2004.04.043 15149638

[B59] Schmittel M. Lüning U. Meder M. Ganz A. Michel C. Herderich M. (1997). Synthesis of sterically encumbered 2, 9-diaryl substituted phenanthrolines. Key building blocks for the preparation of mixed (Bis-heteroleptic) phenanthroline copper(I) complexes. Heterocycl. Comm. 3 (6), 493–498. 10.1515/hc.1997.3.6.493

[B60] Senese S. Zaragoza K. Minardi S. Muradore I. Ronzoni S. Passafaro A. (2007). Role for histone deacetylase 1 in human tumor cell proliferation. Mol. Cell. Biol. 27 (13), 4784–4795. 10.1128/mcb.00494-07 17470557 PMC1951481

[B61] Sheldrick G. M. (2007). A short history ofSHELX. Acta Crystallogr. A 64 (1), 112–122. 10.1107/s0108767307043930 18156677

[B62] Sheldrick G. M. (2015). SHELXT**-**Integrated space-group and crystal-structure determination. Acta Crystallogr. A Found. Adv. 71 (1), 3–8. 10.1107/s2053273314026370 25537383 PMC4283466

[B63] Shen Q. Ye W. Hu X. Zhao C. Zhou L. Zhu X. (2016). The effects of guizhi fuling capsule drug serum on uterine leiomyoma cells and its mechanism. Evid. Based. Complement. Altern. Med. 2016, 2393640. 10.1155/2016/2393640 PMC5118522 27895695

[B64] Shouksmith A. E. Gawel J. M. Nawar N. Sina D. Raouf Y. S. Bukhari S. (2019). Class I/IIb-Selective HDAC inhibitor exhibits oral bioavailability and therapeutic efficacy in acute myeloid leukemia. ACS Med. Chem. Lett. 11 (1), 56–64. 10.1021/acsmedchemlett.9b00471 31938464 PMC6956385

[B65] Solomon E. I. Sundaram U. M. Machonkin T. E. (1996). Multicopper oxidases and oxygenases. Chem. Rev. 96 (7), 2563–2606. 10.1021/cr950046o 11848837

[B86] Sun Y. Lu Y. Bian M. Yang Z. Ma X. Liu W. (2022). Pt(II) and Au(III) complexes containing Schiff-base ligands: A promising source for antitumor treatment. Eur. J. Med. Chem. 211, 113098. 10.1016/j.ejmech.2020.113098 33348237

[B66] IARC Stewart B. W. Wild C. P. (2014). World health organization. Lyon, France: IARC. Ch. 1.1.

[B67] Tang Z. Ding S. Huang H. Luo P. Qing B. Zhang S. (2017). HDAC1 triggers the proliferation and migration of breast cancer cells *via* upregulation of interleukin-8. Biol. Chem. 398 (12), 1347–1356. 10.1515/hsz-2017-0155 28779562

[B68] Uba A. I. Yelekçi K. (2017). Identification of potential isoform-selective histone deacetylase inhibitors for cancer therapy: A combined approach of structure-based virtual screening, ADMET prediction and molecular dynamics simulation assay. J. Biomol. Struct. Dyn. 36 (12), 3231–3245. 10.1080/07391102.2017.1384402 28938863

[B69] Walsh C. T. Garneau-Tsodikova S. Howard-Jones A. R. (2006). Biological formation of pyrroles: Nature’s logic and enzymatic machinery. Nat. Prod. Rep. 23 (4), 517–531. 10.1039/b605245m 16874387

[B70] Wang Z. Li B. Zhou L. Yu S. Su Z. Song J. (2016). Prodigiosin inhibits Wnt/β-catenin signaling and exerts anticancer activity in breast cancer cells. Proc. Natl. Acad. Sci. U. S. A. 113 (46), 13150–13155. 10.1073/pnas.1616336113 27799526 PMC5135380

[B71] Weichert W. (2009). HDAC expression and clinical prognosis in human malignancies. Cancer Lett. 280 (2), 168–176. 10.1016/j.canlet.2008.10.047 19103471

[B72] World Health Organization (WHO) (2022). Cancer. Available at: https://www.who.int/health-topics/cancer#tab=tab_1 (Accessed March 13, 2022).

[B73] Yang H. Rudge D. G. Koos J. D. Vaidialingam B. Yang H. J. Pavletich N. P. (2013). mTOR kinase structure, mechanism and regulation. Nature 497 (7448), 217–223. 10.1038/nature12122 23636326 PMC4512754

[B74] Yao D. Jiang J. Zhang H. Huang Y. Huang J. Wang J. (2021). Design, synthesis and biological evaluation of dual mTOR/HDAC6 inhibitors in MDA-MB-231 cells. Bioorg. Med. Chem. Lett. 47, 128204. 10.1016/j.bmcl.2021.128204 34139324

[B75] Yoneda T. Williams P. J. Hiraga T. Niewolna M. Nishimura R. (2001). A bone-seeking clone exhibits different biological properties from the MDA-MB-231 parental human breast cancer cells and a brain-seeking clone *in vivo* and *in vitro* . J. Bone Min. Res. 16 (8), 1486–1495. 10.1359/jbmr.2001.16.8.1486 11499871

[B76] Zhang C. X. Lippard S. J. (2003). New metal complexes as potential therapeutics. Curr. Opin. Chem. Biol. 7 (4), 481–489. 10.1016/s1367-5931(03)00081-4 12941423

[B77] Zhang X. Bi C. Fan Y. Cui Q. Chen D. Xiao Y. (2008). Induction of tumor cell apoptosis by taurine Schiff base copper complex is associated with the inhibition of proteasomal activity. Int. J. Mol. Med. 22 (5), 677–682. PMID: 18949390; PMCID: PMC3777612. 10.3892/ijmm_00000072 18949390 PMC3777612

[B78] Zhang Z. Bi C. Fan Y. Zhang N. Deshmukh R. Yan X. (2015). L-Ornithine Schiff base-copper and -cadmium complexes as new proteasome inhibitors and apoptosis inducers in human cancer cells. J. Biol. Inorg. Chem. 20, 109–121. doi: 10.1007/s00775-014-1219-1 25467055

[B79] Zhang Z. Bi C. Schmitt S. M. Fan Y. Dong L. Zuo J. (2012). 1, 10-Phenanthroline promotes copper complexes into tumor cells and induces apoptosis by inhibiting the proteasome activity. J. Biol. Inorg. Chem. 17 (8), 1257–1267. 10.1007/s00775-012-0940-x 23053530 PMC3662054

[B80] Zhang Z. Wang H. Yan M. Wang H. Zhang C. (2017). Novel copper complexes as potential proteasome inhibitors for cancer treatment (Review). Mol. Med. Rep. 15 (1), 3–11. doi: 10.3892/mmr.2016.6022 27959411

[B81] Zhong W. Tang Y. Zampella G. Wang X. Yang X. Hu B. (2010). A rare bond between a soft metal (FeI) and a relatively hard base (RO−, R = phenolic moiety). Inorg. Chem. Commun. 13 (9), 1089–1092. 10.1016/j.inoche.2010.06.026

[B82] Zoroddu M. A. Zanetti S. Pogni R. Basosi R. (1996). An electron spin resonance study and antimicrobial activity of copper(II)-phenanthroline complexes. J. Inorg. Biochem. 63 (4), 291–300. 10.1016/0162-0134(96)00015-3 8757142

[B83] Zuo J. Bi C. Fan Y. Buac D. Nardon C. Daniel K. G. (2013). Cellular and computational studies of proteasome inhibition and apoptosis induction in human cancer cells by amino acid Schiff base-copper complexes. J. Inorg. Biochem. 118, 83–93. 10.1016/j.jinorgbio.2012.10.006 23142973 PMC3676669

