# Supplementary material for: Design, synthesis, molecular modeling, and bioactivity evaluation of 1,10-phenanthroline and prodigiosin (Ps) derivatives and their Copper(I) complexes against mTOR and HDAC enzymes as highly potent and effective new anticancer therapeutic drugs
Source: Front Pharmacol. 2022 Oct 4;13:980479. doi: 10.3389/fphar.2022.980479 (PMC9578020; doi:10.3389/fphar.2022.980479)
Supplement: Supplementary file 1 [file DataSheet1.pdf]

Supporting Information

for

Design, Synthesis, Molecular Modeling and Bioactivity Evaluation of 1,10-Phenanthroline and Prodigiosin (Ps) Derivatives and Their Copper(I) Complexes against mTOR and HDAC Enzymes as Highly Potent and Effective New Anticancer Therapeutic Drugs

M. Mustafa Cetin,[1,*] Wenjing Peng,[2] Daniel Unruh,[2] Michael F. Mayer,[2] Yehia Mechref,[2,*] Kemal Yelekci,[1,*]

[1]*Department of Molecular Biology and Genetics, Faculty of Engineering and Natural Sciences, Kadir Has University, Cibali Campus Fatih, Istanbul, Turkey*

[2]*Department of Chemistry and Biochemistry, Texas Tech University, Lubbock, TX, USA*

*E-mail (corresponding authors): mustafa.cetin@khas.edu.tr, yelekci@khas.edu.tr and yehia.mechref@ttu.edu

Table of Contents

Section A. **Materials / General Methods / Instrumentation** **S2**

Section B. **Synthetic Protocols** **S3**

Section C. **NMR Spectroscopy** **S9**

Section D. **Computational Calculations and Molecular Modeling Studies** **S13**

Section E. **Anticancer Activities and Cytotoxicity Tests** **S16**

Section F. **Crystallographic Characterization** **S34**

Section G. **References** **S41**

Section A. Materials / General Methods / Instrumentation

All chemicals and reagents were purchased from commercial suppliers (Aldrich, Alfa Aesar or Fisher) and used without further purification. Anhydrous dichloromethane (DCM) and acetonitrile were separately distilled over CaH_2 under nitrogen. Dioxane was separately distilled over Na/benzophenone under nitrogen. Thin layer chromatography (TLC) was performed on silica gel 60 F254 (E. Merck). Column chromatography was carried out on silica gel 60F (Merck 9385, 0.040–0.063 mm). Ligands (**L^1-L^6**) (Cetin, 2017; Dietrich-Buchecker and Sauvage, 1990; Zhong et al., 2010; Kang et al., 2014; Kohler et al., 2016; Kohler et al., 2017; Hayes et al., 2018; Hayes et al., 2018; Schmittel et al., 1997; Cetin et al., 2017; Cetin et al., 2020) precursors (Cetin, 2017; Kang et al., 2014; Cetin et al., 2017; Cetin et al., 2020; Melvin et al., 2002; Kang et al., 2008) for ligands (**L^7-L^{15}**), and complexes (**C1-C15**) (Cetin, 2017; Dietrich-Buchecker and Sauvage, 1990; Kang et al., 2014; Cetin et al., 2017; Cetin et al., 2020; Kang et al., 2008) were prepared according to previous literature procedures with slight/moderate/complete modifications. All details for synthetic procedures are described in the **Section B**.

Proton and carbon nuclear magnetic resonance (^{1}H, ^{19}F and ^{13}C NMR) spectra were recorded on JEOL ECS–400 or a Varian Unity Inova 500 spectrometer, with working frequency of 400 or 500 MHz for ^{1}H, 100 or 125 MHz for ^{13}C, and 376 or 471 MHz for ^{19}F nuclei, respectively. Chemical shifts are reported in ppm relative to the signals corresponding to the residual non–deuterated solvent ($CDCl_3$ (99.9% D with 0.05% v/v TMS): δ = 7.24 ppm for ^{1}H NMR, and ($CDCl_3$ (99.9% D with 0.05% v/v TMS): δ = 77.16 ppm for ^{13}C NMR). Coupling constants, *J*, are reported in hertz. High-resolution ESI mass spectrometry was performed on an Exactive-Orbitrap mass spectrometer at Texas Tech University (Lubbock, TX). Flash

chromatography was performed using Silicycle UltraPure Flash Silica Gel (60 Å, 40-63 μm). Thin layer chromatography (TLC) was performed using EMD HPTLC plates, silica gel 60, F_{254}. All reaction vessels were flame-dried under vacuum and filled with nitrogen prior to use. All reactions were performed under a nitrogen atmosphere as a routine practice, not as an essential requirement.

Section B. Synthetic Protocols

The detailed synthetic procedures and the structural characterization data for the intermediates and target compounds are presented below.

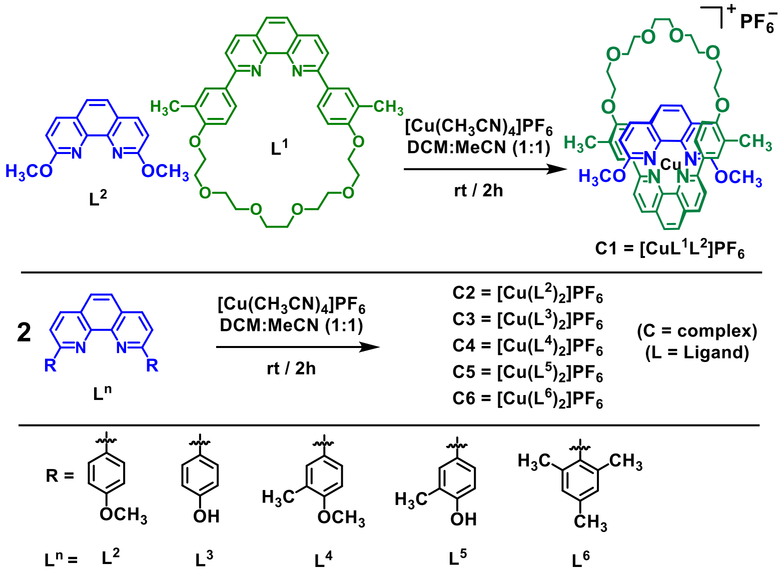

Scheme S1 | Synthesis of the copper(I) complexes (**C1-C6**) — 2:1 ligand-to-metal complexes, as PF_6^- salts — from their respective 1,10-phenanthroline-based ligand derivatives (**L^1-L^6**).

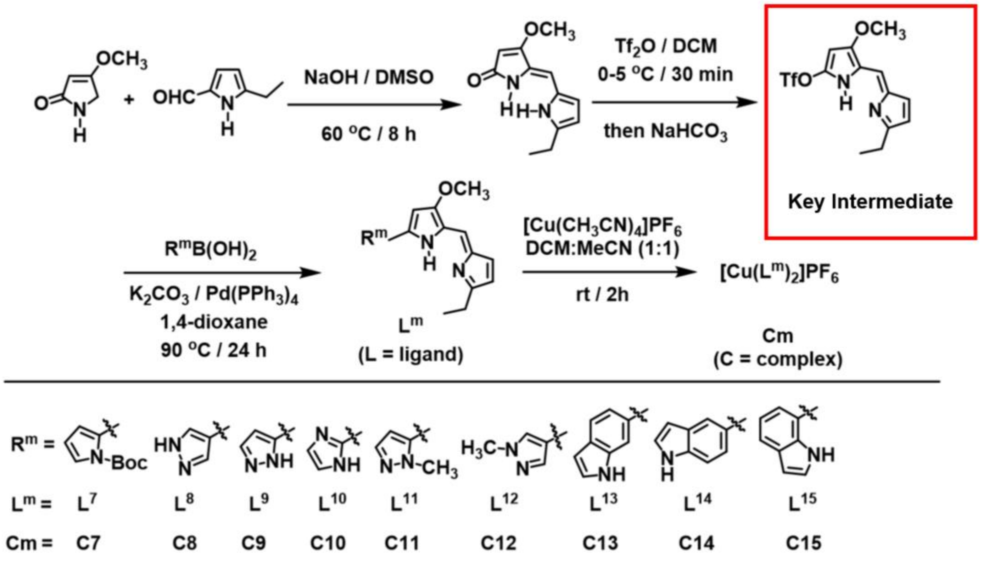

Scheme S2|Synthesis of the Prodigiosin (**Ps**) derivatives (**L⁷-L¹⁵**) and their respective 2:1 ligand-to-metal copper(I) complexes (**C7-C15**), as PF_6^- salts.

Synthesis of 4-methoxy-5-(5-ethyl-1H-pyrrol-2-ylmethylidene)-1,5-dihydropyrrol-2-one:

To a solution of 2-formyl-5-ethylpyrrole (0.500 grams, 4.06 mmol) and 4-methoxy-3-pyrrolin-2-one (0.920 grams, 8.13 mmol) in 20 mL DMSO was added 2N aq. NaOH (15 mL) and the mixture was stirred at 60 °C for 8 hours. After dilution with 100 mL DI-water, the suspension was extracted with 300 mL dichloromethane (3 x 100 mL). The organic phase was washed (shaken) with saturated brine and DI-water, dried over Na_2SO_4, and evaporated to dryness. The crude was dissolved in 2-3 mL dichloromethane and then excess hexane (50 mL) was added. The solution was evaporated under vacuum at 45 °C until 15-20 mL hexane was left. The black solid formation occurred. It was filtered and solid was collected over the filter paper. Then it was dissolved in 2-3 mL dichloromethane again and excess hexane (50 mL) was added into it. The solution was started to concentrate by evaporating under vacuum at 45 °C until 15-20 mL hexane was left again. Formation of brown solid was observed. The solid was collected over filter paper, dried, and left under vacuum overnight. After checking the proton NMR of the solid, 0.696 grams (78%) pure

product was obtained. ^{1}H NMR (400 MHz, $CDCl_3$, 25 ºC) δ 10.8 (s, 1H), 10.31 (s, 1H), 6.37 (t, *J* = 3.2 Hz, 1H), 6.32 (s, 1H), 5.99 (t, *J* = 2.7 Hz, 1H), 5.10 (d, *J* = 2.0 Hz, 1H), 3.90 (s, 3H), 2.79 (q, *J* = 15 and 7.3 Hz, 2H), 1.34 (t, *J* = 7.9 Hz, 3H); ^{13}C NMR (100 MHz, $CDCl_3$, 25 ºC) δ 173.13, 168.00, 141.98, 125.63, 123.98, 117.38, 107.01, 102.75, 90.12, 58.18, 21.30, 13.71.

Synthesis of 2-trifluoromethansulfonyloxy-4-methoxy-5-[(5-ethyl-2H-pyrrol-2-ylidene)methyl]-1H-pyrrole (Key Intermediate):

To a solution of 4-methoxy-5-(5-ethyl-1H-pyrrol-2-ylmethylidene)-1,5-dihydropyrrol-2-one (0.696 grams, 3.19 mmol) in dichloromethane (55 mL) at 0-5 ºC was added Tf_2O (0.1.80 grams (0.650 mL), 6.37 mmol) dropwise under nitrogen atmosphere. After stirring at this temperature for 30 minutes, the reaction mixture was poured into a 2% aq. $NaHCO_3$ solution, and extracted with ethyl acetate (2 x 50 mL). Then solvent was removed and the crude was left under vacuum for 2-3 hours. After dissolving the crude in ethyl acetate (50 mL), the solution was washed with saturated brine solution. After separating the organic layer, it was dried over anhydrous Na_2SO_4 and evaporated to the dryness. The crude was chromatographed on silicagel eluting with 50:50 hexane:dichloromethane solvent mixture. The pure 2-trifluoromethansulfonyloxy-4-methoxy-5-[(5-ethyl-2H-pyrrol-2-ylidene)methyl]-1H-pyrrole was obtained (1.03 grams, 92%); ^{1}H NMR (400 MHz, $CDCl_3$) δ 10.8 (s, 1H), 7.03 (s, 1H), 6.66 (d, *J* = 3.6 Hz, 1H), 6.07 (d, *J* = 3.7 Hz, 1H), 5.40 (s, 1H), 3.88 (s, 3H), 2.74 (q, *J* = 15 and 7.3 Hz, 2H), 1.32 (t, *J* = 7.8 Hz, 3H); ^{13}C NMR (100 MHz, $CDCl_3$) δ 168.07, 161.21, 146.65, 132.44, 128.54, 123.20, 122.20, 120.39, 109.75, 87.18, 58.82, 21.61, 12.73. ; ^{19}F NMR (376 MHz, $CDCl_3$) δ -72.7 (s, $-CF_3$).

Synthesis of 2-(1-Boc-pyrrol-2-yl)-4-methoxy-5-[(5-ethyl-2H-pyrrol-2-ylidene)methyl]-1H-pyrrole:

An oxygen-free solution of 2-trifluoromethansulfonyloxy-4-methoxy-5-[(5-ethyl-2H-pyrrol-2-ylidene)methyl]-1H-pyrrole (0.142 g, 0.404 mmol) in dry and freshly distilled 1,4-dioxane (30 mL) was treated in sequence with 1-Boc-pyrrole-2-boronic acid (0.341 g, 1.62 mmol), K_2CO_3 (0.446 g, 3.23 mmol). The solution purged with nitrogen for 10 mins and then $Pd(PPh_3)_4$ (23.3 mg, 5mol%) was added, and then the reaction mixture was heated to 90 ºC under nitrogen atmosphere with stirring for 24 hours. After cooling to room temperature, the reaction mixture was poured into ice-water (50 mL) and extracted with ethyl acetate (4 x 50 mL). The organic phase was washed (shaken) with saturated brine solution and DI-water, then dried over anhydrous sodium sulfate. Then it was evaporated to dryness and kept under vacuum for 2-3 hours. The crude was then columned with alumina (activated) by eluting 100 % hexane (300 mL) and then hexane:ethyl acetate (85:15) to purify the product. It was columned over activated alumina twice to get pure product (67.7 mg, 46%); ^{1}H NMR (400 MHz, $CDCl_3$) δ 6.84 (s, 1H), 6.72 (s, 1H), 6.68 (dd, *J* = 3.7 and 1.4 Hz, 1H), 6.49 (d, *J* = 3.6 Hz, 1H), 6.17 (t, *J* = 2.3 Hz, 1H), 6.04 (s, 1H), 5.89 (d, *J* = 3.7 Hz, 1H), 3.96 (s, 3H), 3.67 (s, 1H), 2.30 (q, *J* = 15 and 7.3 Hz, 2H), 1.26 (s, 9H), 1.05 (t, *J* = 7.8 Hz, 3H); ^{13}C NMR (100 MHz, $CDCl_3$) δ 168.95, 145.30, 128.75, 128.74, 122.74, 120.64, 115.95, 112.78, 110.31, 108.13, 95.59, 58.52, 37.50, 32.02, 29.79, 22.78, 20.31, 14.22, 13.36.

Synthesis of dimethylated macrocycle (L^1):

In a flame-dried round-bottom flask, a mixture of dimethylated diphenol (10.0 g, 25.4 mmol) and 1,14-diiodo-3,6,9,12-tetraoxatetradecane (12.4 g, 27.0 mmol) in DMF (400 mL) was added drop wise within 24 hours under efficient stirring to a nitrogen flushed suspension of Cs_2CO_3 (25.6 g, 72.6 mmol) in DMF (150 mL) kept at 55-60 °C. At the end of the addition, stirring was continued for another 48 hours at the same temperature. DMF was removed under reduced pressure with a rotary evaporator. The yellowish residue was dissolved in 150 mL of DCM, washed with saturated

aq. NH_4Cl (3 x 100 mL), dried over anhydrous Na_2SO_4 and filtered. The solvent was evaporated under reduced pressure by a rotary evaporator to leave a yellow solid that was purified on silica gel by flash column chromatography using DCM-methanol (99.5:0.5) to provide dimethylated macrocycle **L^1** (12.5 g, 82%) as a bright yellow solid, m.p. 153.8-155.1 °C; ^{1}H NMR (500 MHz, $CDCl_3$) δ 8.37 (dd, 2.0, 2.0 Hz, 2H), 8.26-8.24 (m, 4H), 8.07 (d, *J* = 8.0 Hz, 2H), 7.74 (s, 2H), 7.15 (d, *J* = 8.5 Hz, 2H), 4.34 (t, *J* = 5.0 Hz, 5.5 Hz, 4H), 3.84 (t, *J* = 5.5 Hz, 5.0 Hz, 4H), 3.75-3.69 (m, 12H), 2.42 (s, 6H); ^{13}C NMR (125 MHz, $CDCl_3$) 158.37, 156.40, 145.88, 136.53, 132.35, 130.14, 127.64, 127.27, 126.51, 125.41, 119.04, 112.95, 71.02, 70.66, 70.56, 69.52, 68.28, 16.69; HRMS (ESI) calcd for $C_{36}H_{39}N_2O_6$ $[M+H]^+$ m/z 595.2803, found m/z 595.2800; Anal. calcd. for $C_{36}H_{38}N_2O_6$: C, 72.71; H, 6.44; N, 4.71; found: C, 72.33; H, 6.51; N, 4.66.

General procedure for the syntheses of complexes:

A solution of the ligand (**L^2-L^{15}**) (0.250 mmol) in DCM (10 mL) and acetonitrile (10 mL) was prepared at room temperature under a nitrogen atmosphere. The light yellow-colored solution was stirred until the ligand was dissolved completely. To this solution, tetrakis(acetonitrile)copper(I) hexafluorophosphate (0.125 mmol) was added and the solution was stirred for 20 minutes at room temperature. The color of the solution turned to a dark brown-red-black. Concentration of the mixture under reduced pressure using a rotary evaporator provided the crude product. Purification on silica gel by flash column chromatography, using DCM–methanol (99:1) as eluent, afforded the corresponding copper(I) complex, **$[Cu(L^n)_2]PF_6$** (**C2-C15**), where **L^n** = **L^2-L^{15}**.

In the synthesis of **C1**, to a solution of dimethylated macrocycle **L^1** (205 mg, 0.362 mmol) in dichloromethane (10 mL) and acetonitrile (10 mL) at room temperature under nitrogen was added tetrakis(acetonitrile)copper(I) hexafluorophosphate (123 mg, 0.463 mmol) and stirred for 20 min.

A dicholoromethane (5 mL) and acetonitrile (5 mL) solution of $\mathbf{L^2}$ (142 mg, 0.362 mmol) was added from another Schlenk flask under nitrogen via cannula. The reaction mixture was stirred for two hours at room temperature under nitrogen followed by concentration of the mixture under reduced pressure by a rotary evaporator. Purification on SEC column chromatography using DCM as eluent afforded the partially oxidized the complex $[CuL^1L^2]PF_6$ (**C1**). The partially oxidized product was dissolved in 10 mL DCM, and 5 mg sodium dithionite (90%), and five drops of 2N aqueous sodium hydroxide were added to the solution and the mixture was stirred for 30 min at room temperature. After 30 min, the solution was filtered through a fritted-funnel which was filled with 1 cm height of Celite 545 and 1 cm height of anhydrous Na_2SO_4 to provide reduced and pure $[CuL^1L^2]PF_6$ (**C1**) (400 mg, 98%) as a red glassy solid, m.p. 252.4-253.0 °C; ^{1}H NMR (500 MHz, $CDCl_3$) δ 8.64 (d, *J* = 8.5 Hz, 2H), 8.46 (d, *J* = 8.0 Hz, 2H), 8.22 (s, 2H), 8.00 (s, 2H), 7.89 (d, *J* = 8.0 Hz, 2H), 7.80 (d, *J* = 8.0 Hz, 2H), 7.51 (d, *J* = 8.5 Hz, 4H), 7.18 (d, *J* = 8.5 Hz, 2H), 6.95 (s, 2H), 6.08 (d, *J* = 8.5 Hz, 4H), 5.81 (d, *J* = 8.0 Hz, 2H), 3.88 (s, 4H), 3.76-3.74 (m, 4H), 3.67-3.64 (m, 8H), 3.61-3.59 (m, 4H), 3.52 (s, 6H), 1.51 (s, 6H); ^{13}C NMR (125 MHz, $CDCl_3$) δ 160.28, 157.24, 157.06, 155.74, 143.46, 137.81, 136.86, 132.22, 131.20, 130.30, 129.28, 129.28, 128.16, 127.84, 127.20, 126.56, 126.09, 125.85, 124.19, 112.58, 112.58, 109.38, 71.31, 71.06, 71.06, 69.52, 67.52, 55.38, 15.88 ; HRMS (ESI) calcd for $C_{62}H_{58}CuN_4O_8$ $[M-PF_6]^+$ m/z 1049.3545, found m/z 1049.3521; Anal. calcd. for $C_{62}H_{58}CuF_6N_4O_8P$: C, 62.28; H, 4.89; N, 4.69; found: C, 62.35; H, 4.69; N, 4.62.

Section C. NMR Spectroscopy

^{1}H NMR Spectrum of 4-methoxy-5-(5-ethyl-1H-pyrrol-2-ylmethylidene)-1,5-dihydropyrrol-2-one

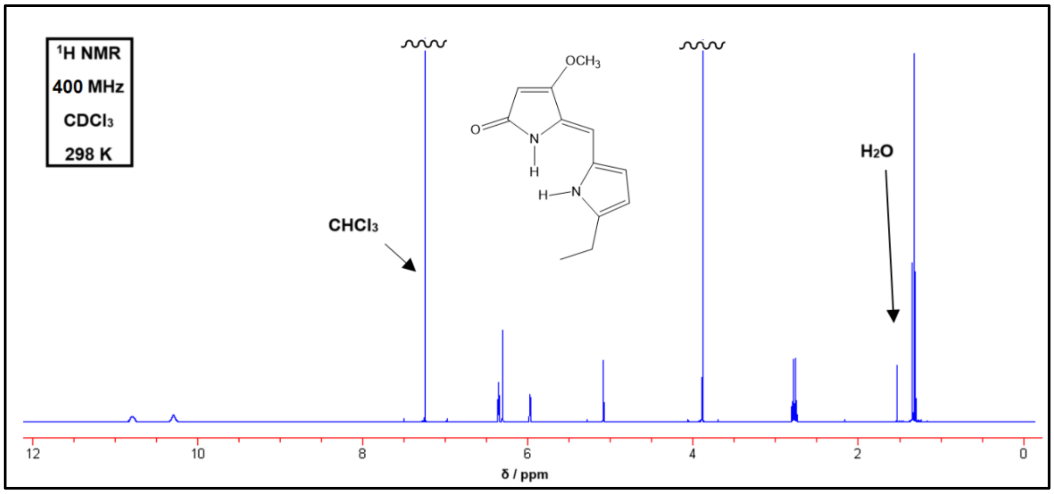

Figure S1 | Annotated ^{1}H NMR spectrum (400 MHz, $CDCl_3$, 25 ºC) of 4-methoxy-5-(5-ethyl-1H-pyrrol-2-ylmethylidene)-1,5-dihydropyrrol-2-one.

^{13}C NMR Spectrum of 4-methoxy-5-(5-ethyl-1H-pyrrol-2-ylmethylidene)-1,5-dihydropyrrol-2-one

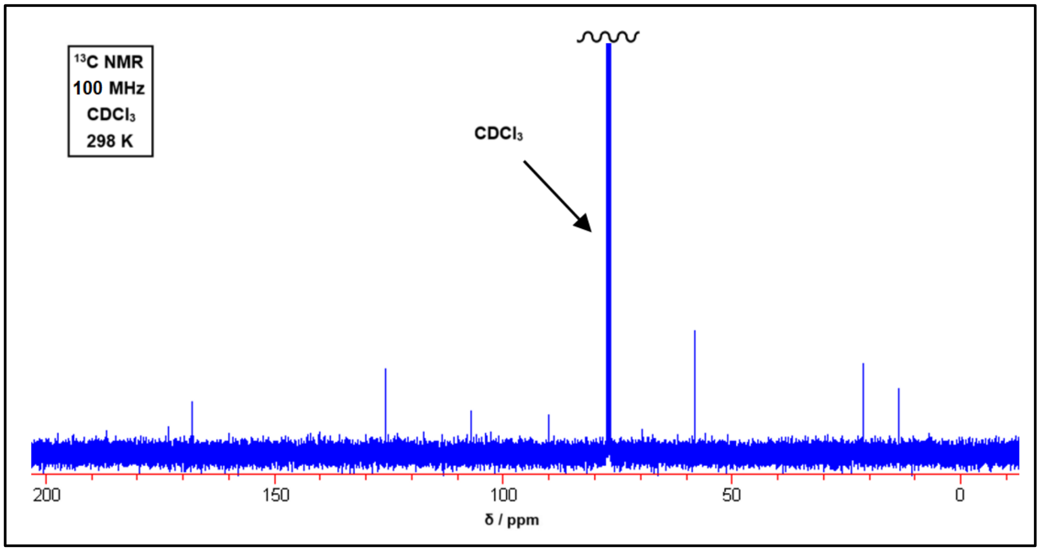

Figure S2 | Annotated ^{13}C NMR spectrum (100 MHz, $CDCl_3$, 25 ºC) of 4-methoxy-5-(5-ethyl-1H-pyrrol-2-ylmethylidene)-1,5-dihydropyrrol-2-one.

^{1}H NMR Spectrum of 2-trifluoromethansulfonyloxy-4-methoxy-5-[(5-ethyl-2H-pyrrol-2-ylidene)methyl]-1H-pyrrole (Key Intermediate):

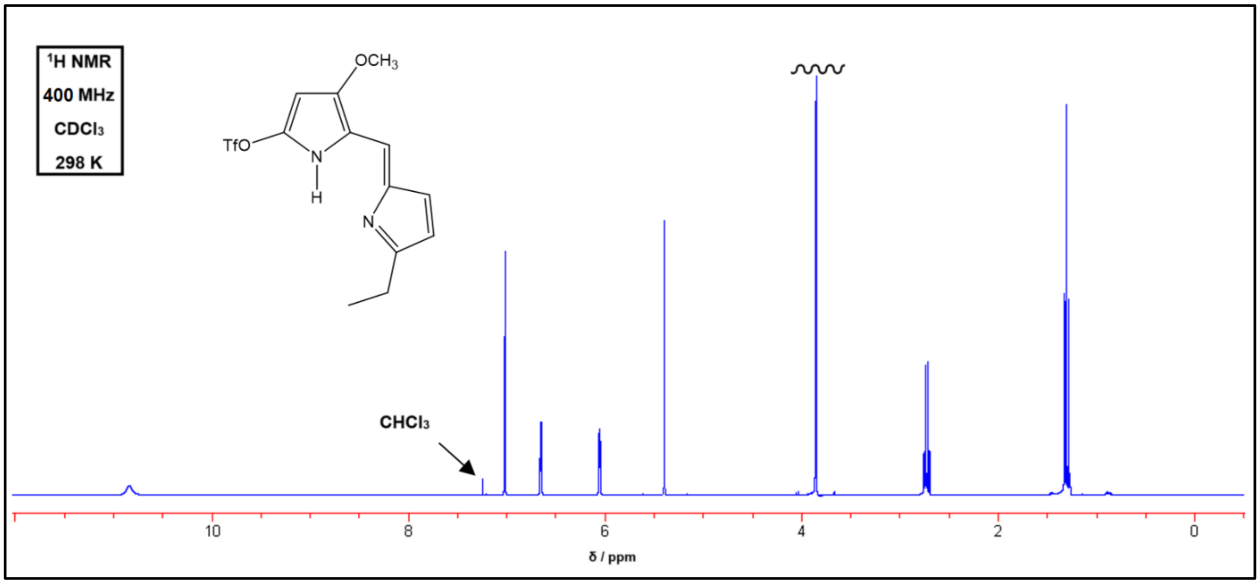

Figure S3|Annotated ^{1}H NMR spectrum (400 MHz, $CDCl_3$, 25 ºC) of 2-trifluoromethansulfonyloxy-4-methoxy-5-[(5-ethyl-2H-pyrrol-2-ylidene)methyl]-1H-pyrrole.

^{13}C NMR Spectrum of 2-trifluoromethansulfonyloxy-4-methoxy-5-[(5-ethyl-2H-pyrrol-2-ylidene)methyl]-1H-pyrrole (Key Intermediate):

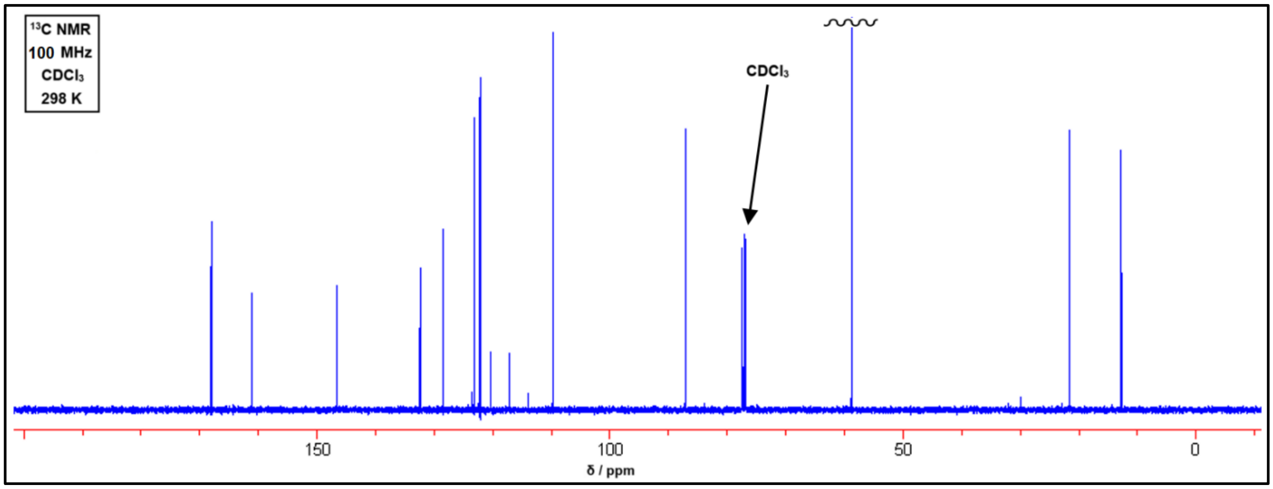

Figure S4|Annotated ^{13}C NMR spectrum (100 MHz, $CDCl_3$, 25 ºC) of 2-trifluoromethansulfonyloxy-4-methoxy-5-[(5-ethyl-2H-pyrrol-2-ylidene)methyl]-1H-pyrrole.

^{1}H NMR Spectrum of 2-(1-Boc-pyrrol-2-yl)-4-methoxy-5-[(5-ethyl-2H-pyrrol-2-ylidene)methyl]-1H-pyrrole:

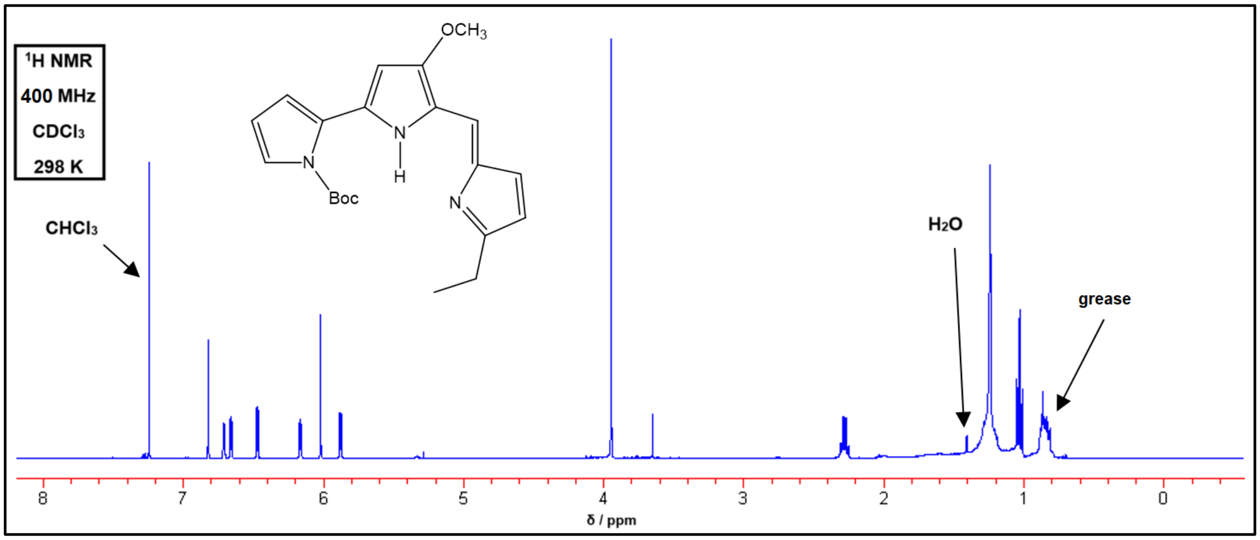

Figure S5 | Annotated ^{1}H NMR spectrum (400 MHz, $CDCl_3$, 25 ºC) of 2-(1-Boc-pyrrol-2-yl)-4-methoxy-5-[(5-ethyl-2H-pyrrol-2-ylidene)methyl]-1H-pyrrole.

^{13}C NMR Spectrum of 2-(1-Boc-pyrrol-2-yl)-4-methoxy-5-[(5-ethyl-2H-pyrrol-2-ylidene)methyl]-1H-pyrrole:

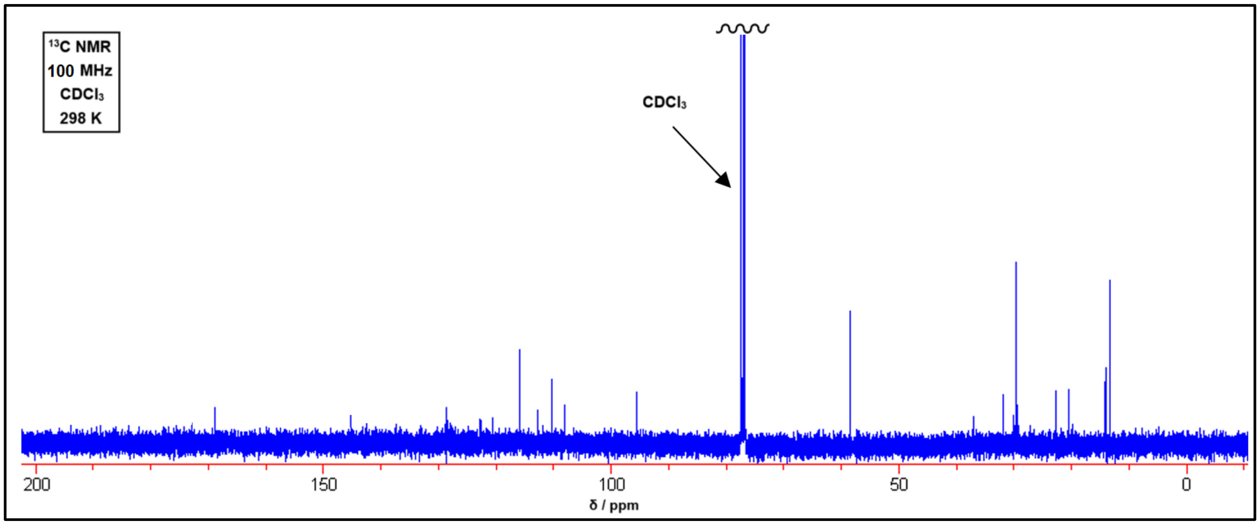

Figure S6 | Annotated ^{13}C NMR spectrum (100 MHz, $CDCl_3$, 25 ºC) of 2-(1-Boc-pyrrol-2-yl)-4-methoxy-5-[(5-ethyl-2H-pyrrol-2-ylidene)methyl]-1H-pyrrole.

^{1}H NMR Spectrum of dimethylated macrocycle (C1)

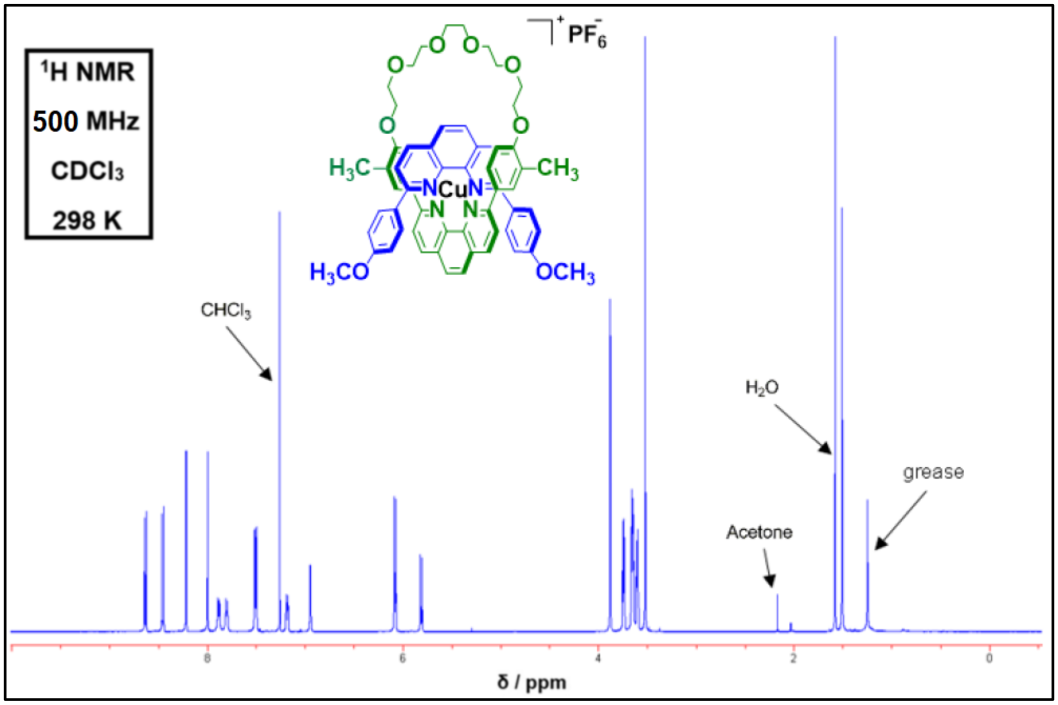

Figure S7|Annotated ^{1}H NMR spectrum (500 MHz, $CDCl_3$, 25 ºC) of dimethylated macrocycle (**C1**).

^{13}C NMR Spectrum of dimethylated macrocycle (C1)

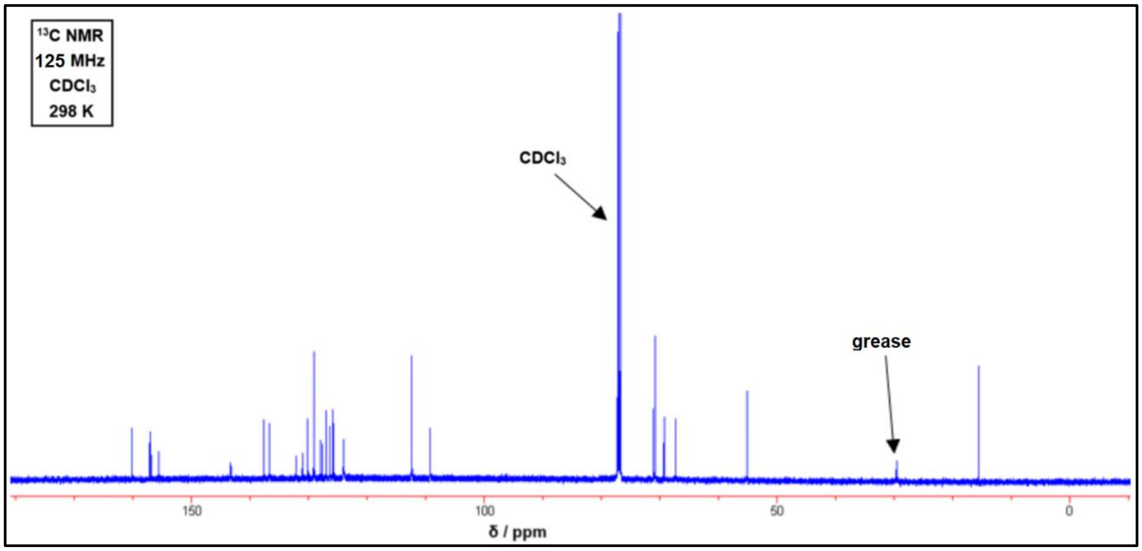

Figure S8|Annotated ^{13}C NMR spectrum (125 MHz, $CDCl_3$, 25 ºC) of dimethylated macrocycle (**C1**).

Section D. Computational Calculations and Molecular Modeling Studies

Table S1 | Calculated binding energies (in kcal/mol) and inhibition constants (in μM, except **C7** being in mM) for commercially available **Taxol**, **Ps**, ligands **L**^{2}–**L**^{15} and copper(I) complexes **C1**–**C15**.

**Compound ID**	**mTOR**		**HDAC1**	
	Binding Energies (kcal/mol)	Inhibition Constants (μM)	Binding Energies (kcal/mol)	Inhibition Constants (μM)
**Taxol** (commercial)	– 4.63	405.16	– 4.72	348.25
**Ps**	– 4.89	258.68	– 6.99	7.53
**L**^{2}	– 6.43	19.30	– 8.00	1.37
**L**^{3}	– 6.53	16.27	– 7.76	2.05
**L**^{4}	– 6.61	14.31	– 7.17	5.52
**L**^{5}	– 6.97	7.72	– 8.28	0.849
**L**^{6}	– 6.46	18.42	– 7.83	1.82
**L**^{7}	– 5.04	202.51	– 7.06	6.64
**L**^{8}	– 5.06	194.81	– 7.39	3.86
**L**^{9}	– 4.84	281.77	– 6.35	22.27
**L**^{10}	– 4.69	367.08	– 7.06	6.69
**L**^{11}	– 4.93	242.54	– 7.37	3.99
**L**^{12}	– 4.90	256.87	– 6.66	13.16
**L**^{13}	– 6.06	36.04	– 8.09	1.18
**L**^{14}	– 6.62	14.15	9.08	0.220
**L**^{15}	– 5.93	45.31	– 8.06	1.23
**C1**	– 8.31	0.808	– 6.88	9.10
**C2**	– 6.96	7.91	– 7.50	3.18
**C3**	– 8.32	0.796	– 6.30	23.99
**C4**	– 7.01	7.26	– 6.00	39.99
**C5**	– 9.46	0.117	– 4.86	276.08
**C6**	– 8.01	1.34	– 5.27	137.82
**C7**	– 4.04	1.09 **mM**	– 2.97	6.62 **mM**
**C8**	– 6.51	16.91	– 5.27	137.72
**C9**	– 5.84	52.19	– 4.16	885.39
**C10**	– 5.96	42.90	– 6.12	32.65
**C11**	– 5.30	130.76	– 5.95	43.17
**C12**	– 6.09	34.51	– 5.79	56.94
**C13**	– 6.71	11.99	– 6.57	15.20
**C14**	– 5.90	47.11	– 6.69	12.52
**C15**	– 5.83	53.12	– 4.79	308.28

Table S2|Three-dimensional (3D) images generated via molecular docking of **Ps**, ligands **L^{7}** and **L^{14}** and copper(I) complexes **C1** and **C14**, and two-dimensional (2D) images generated via molecular docking of **Ps**, ligands **L^{7}** and **L^{14}** into mTOR and HDAC1 enzymes.

Compound ID	mTOR	HDAC1
**Ps**	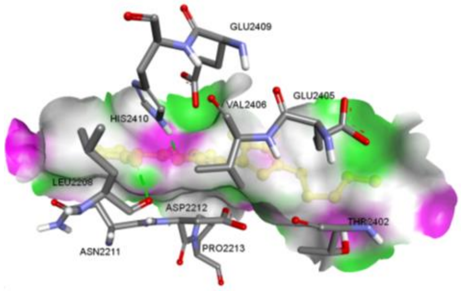	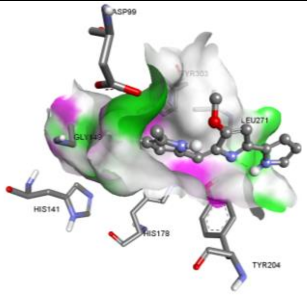
	H-Bond Donor	H-Bond Acceptor
**Ps (2D)**	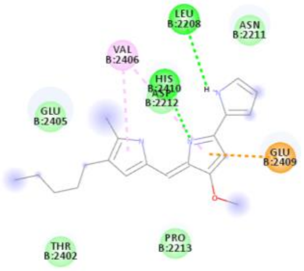	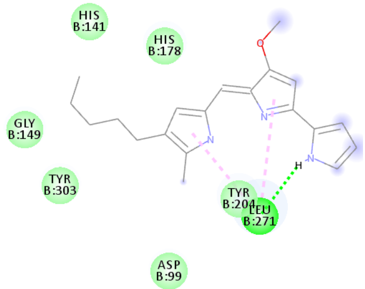
	<u>Interactions</u> van der Waals Conventional Hydrogen Bond	Pi-Anion Pi-Alkyl
**C1**	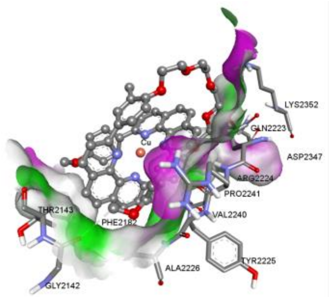	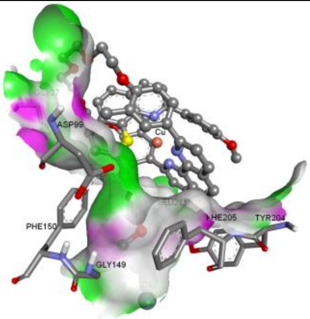
	H-Bond Donor	H-Bond Acceptor
**L^{7}**	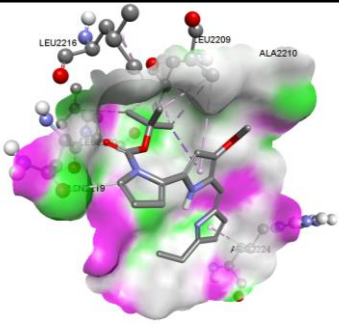	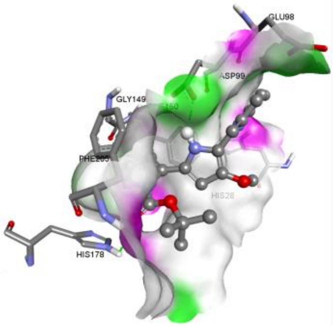
	H-Bond Donor	H-Bond Acceptor

L[7] (2D)

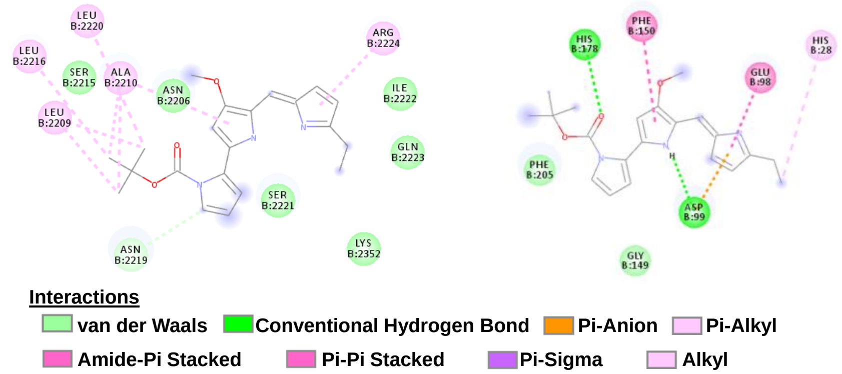

L[14]

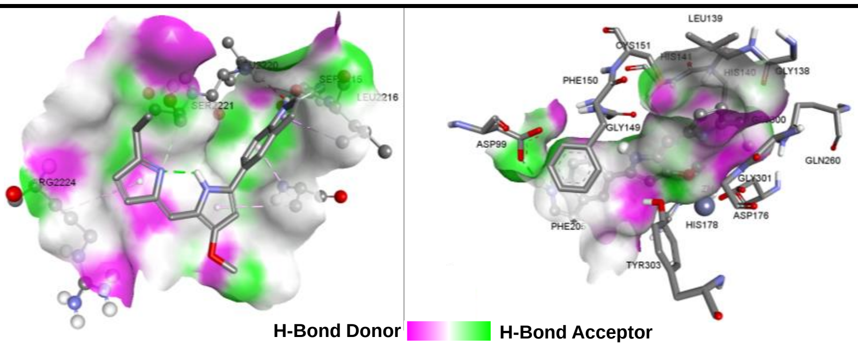

L[14] (2D)

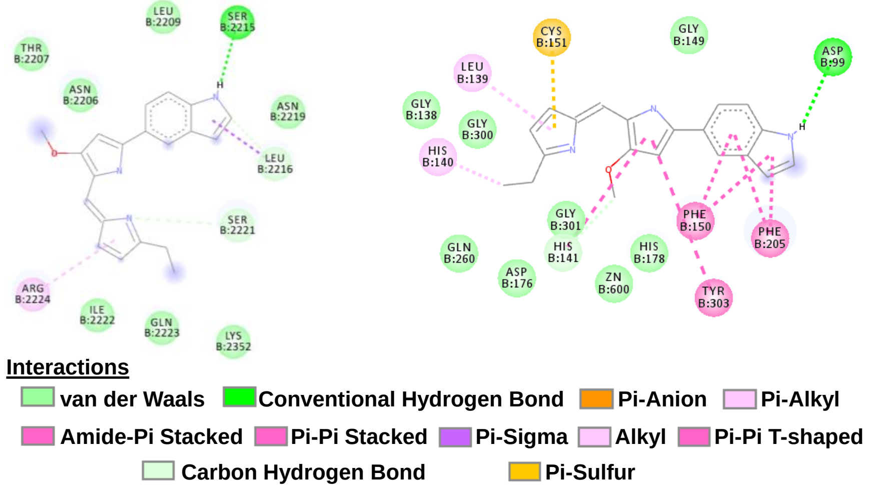

C14

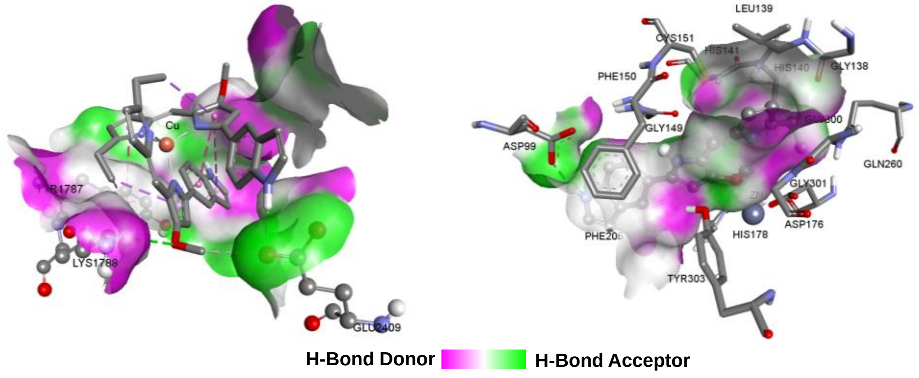

Section E. Anticancer Activities and Cytotoxicity Tests

Table S3|Primary anticancer activity screening of compounds (**L^{14}**, **L^{9}**, **C14**, **C9**, **L^{13}**, **L^{15}**, **L^{10}**, **C15**, **C13**, **C8**, **C10**, **L^{7}**, **Ps**, **Taxol (Paclitaxel)**) on different cell lines.

L^{14}:

	drug concentration (M)						Positive	Negative			
**cell line**	**7.90E-10**	**7.90E-09**	**7.90E-08**	**7.90E-07**	**7.90E-06**	**7.90E-05**	**Control**	**Control**			
**231BR**	0.628	0.596	0.573	0.589	0.603	0.319	0.623	0.160			
	0.604	0.568	0.596	0.596	0.597	0.318	0.624	0.162			
	0.653	0.577	0.601	0.573	0.579	0.314	0.635	0.161			
	0.614	0.673	0.641	0.635	0.623	0.316	0.607	0.162			
	0.638	0.608	0.56	0.611	0.593	0.305	0.625	0.164			
	0.629	0.636	0.622	0.609	0.619	0.317	0.642	0.159			
**average**	0.628	0.610	0.599	0.602	0.602	0.315	0.626	0.161	**Approximate**		
**deviation (SD)**	0.0173	0.0392	0.0300	0.0213	0.0165	0.0051	0.0119	0.0018	**IC50**	**7.08E-05 M**	**7.9E-06 < IC50 < 7.9E-05 M**
**Inhibition ratio**	-0.0036	0.0351	0.0584	0.0513	0.0509	0.6692			**SD**	**3.89E-06**	

	drug concentration (M)						Positive	Negative		
**cell line**	**7.90E-10**	**7.90E-09**	**7.90E-08**	**7.90E-07**	**7.90E-06**	**7.90E-05**	**Control**	**Control**		
**HTB22**	0.325	0.319	0.333	0.331	0.351	0.349	0.343	0.122		
	0.335	0.321	0.317	0.327	0.332	0.329	0.341	0.127		
	0.264	0.331	0.314	0.295	0.343	0.344	0.347	0.126		
	0.336	0.352	0.328	0.333	0.332	0.332	0.318	0.127		
	0.337	0.329	0.32	0.329	0.335	0.335	0.336	0.126		
	0.345	0.335	0.32	0.348	0.345	0.345	0.35	0.124	**Approximate**	
**average**	0.324	0.331	0.322	0.327	0.340	0.339	0.339	0.125	**IC50**	**> 7.9E-05 M**
**deviation (SD)**	0.0299	0.0119	0.0071	0.0174	0.0078	0.0081	0.0114	0.0020		
**Inhibition ratio**	0.0717	0.0366	0.0794	0.0553	-0.0031	0.0000				

	drug concentration (M)						Positive	Negative		
**cell line**	**7.90E-10**	**7.90E-09**	**7.90E-08**	**7.90E-07**	**7.90E-06**	**7.90E-05**	**Control**	**Control**		
**CRL**	1.784	1.674	1.667	1.667	1.682	1.061	1.763	0.161		
	1.748	1.672	1.641	1.639	1.655	1.006	1.784	0.165		
	1.715	1.499	1.585	1.602	1.586	1.053	1.67	0.169		
	1.715	1.611	1.636	1.759	1.662	1.059	1.771	0.165		
	1.737	1.818	1.684	1.698	1.629	1.023	1.81	0.17		
	1.875	1.865	1.87	1.833	1.785	1.016	1.789	0.165	**Approximate**	
**average**	1.762	1.690	1.681	1.700	1.667	1.036	1.765	0.166	**IC50**	**> 7.9E-05 M**
**deviation (SD)**	0.0608	0.1344	0.0988	0.0844	0.0668	0.0241	0.0490	0.0033		
**Inhibition ratio**	0.0017	0.0470	0.0528	0.0409	0.0616	0.4557				

L9:

	drug concentration (M)						Positive	Negative		
**Cell Line**	**1.68E-09**	**1.68E-08**	**1.68E-07**	**1.68E-06**	**1.68E-05**	**1.68E-04**	**Control**	**Control**		
**231BR**	0.692	0.721	0.709	0.697	0.799	0.581	0.739	0.160		
	0.684	0.704	0.692	0.738	0.833	0.633	0.745	0.162		
	0.690	0.703	0.712	0.718	0.825	0.623	0.754	0.161		
	0.688	0.709	0.696	0.708	0.790	0.592	0.730	0.162		
	0.698	0.711	0.700	0.684	0.780	0.618	0.714	0.164		
	0.717	0.710	0.676	0.685	0.836	0.663	0.725	0.159		
**average**	0.695	0.710	0.698	0.705	0.811	0.618	0.735	0.161	**Approximate**	
**deviation (SD)**	0.0118	0.0064	0.0130	0.0208	0.0239	0.0294	0.0144	0.0018	**IC50**	**> 1.68E-04 M**
**Inhibition ratio**	0.0692	0.0433	0.0645	0.0514	-0.1325	0.2026				

	drug concentration (M)						Positive	Negative		
**Cell Line**	**1.68E-09**	**1.68E-08**	**1.68E-07**	**1.68E-06**	**1.68E-05**	**1.68E-04**	**Control**	**Control**		
**HTB22**	0.351	0.327	0.341	0.363	0.348	0.411	0.346	0.126		
	0.322	0.327	0.361	0.351	0.376	0.429	0.356	0.131		
	0.362	0.343	0.303	0.346	0.326	0.399	0.327	0.129		
	0.338	0.342	0.356	0.347	0.330	0.374	0.313	0.130		
	0.290	0.330	0.342	0.340	0.355	0.405	0.329	0.132		
	0.656	0.359	0.397	0.354	0.335	0.430	0.340	0.128		
**average**	0.387	0.338	0.350	0.350	0.345	0.408	0.335	0.129	**Approximate**	
**deviation (SD)**	0.134	0.013	0.031	0.008	0.019	0.021	0.015	0.002	**IC50**	**> 1.675E-04 M**
**Inhibition ratio**	-1.044	-0.808	-0.867	-0.867	-0.842	-1.148				

	drug concentration (M)						Positive	Negative		
**Cell Line**	**1.68E-09**	**1.68E-08**	**1.68E-07**	**1.68E-06**	**1.68E-05**	**1.68E-04**	**Control**	**Control**		
**CRL**	1.614	1.633	1.605	1.643	1.629	1.654	1.685	0.160		
	1.722	1.667	1.653	1.694	1.681	1.628	1.624	0.162		
	1.627	1.670	1.631	1.776	1.715	1.569	1.624	0.161		
	1.690	1.635	1.643	1.758	1.712	1.569	1.588	0.162		
	1.738	1.691	1.637	1.659	1.651	1.623	1.703	0.164		
	1.866	1.777	1.743	1.807	1.707	1.696	1.699	0.159		
**average**	1.710	1.679	1.652	1.723	1.683	1.623	1.654	0.161	**Approximate**	
**deviation (SD)**	0.09140	0.05296	0.04741	0.06697	0.03573	0.04929	0.04805	0.00175	**IC50**	**> 1.675E-04 M**
**Inhibition ratio**	-0.03717	-0.01663	0.00134	-0.04610	-0.01909	0.02065				

C14:

	drug concentration (M)					Positive	Negative	Drug			
**Cell Line**	**3.85E-08**	**3.86E-07**	**3.85E-06**	**3.85E-05**	**3.85E-04**	**Control**	**Control**	**Control**			
**HTB131**	1.259	1.580	1.546	0.719	2.079	1.487	0.078	0.147			
	1.878	1.523	1.960	0.791	1.934	0.101	0.077	0.150			
	1.555	1.385	1.537	0.648	2.010	1.381	0.079	0.162			
	1.478	1.147	1.304	0.711	2.122	1.474	0.076	0.469			
	1.039	1.366	1.407	0.645	2.191	1.541	0.079	0.835			
	1.765	2.030	2.143	0.763	1.901	1.378	0.076		**Approximate**		
**average**	1.333	1.355	1.449	0.713	2.040	1.452	0.078	0.153	**IC50**	**2.28E-05 M**	**3.85E-06 < IC50 < 3.85E-05**
**SD**	0.2325	0.1555	0.1154	0.0591	0.1118	0.0710	0.0014		**SD**	**2.41E-06**	
**Inhibition rate**	0.0868	0.0704	0.0025	0.7677	0.0688						

	drug concentration (M)					Positive	Negative	Drug		
**Cell Line**	**3.85E-08**	**3.86E-07**	**3.85E-06**	**3.85E-05**	**3.85E-04**	**Control**	**Control**	**Control**		
**CRL**	2.238	2.333	2.149	0.726	2.283	2.370	0.128			
	2.431	2.566	2.441	1.136	2.114	2.244	0.131			
	2.158	2.491	2.413	0.906	2.202	2.251	0.128			
	2.444	2.570	2.315	0.867	2.302	2.242	0.129	0.423		
	2.435	2.370	2.377	0.888	2.219	2.240	0.129	1.382		
	2.540	2.541	2.314	0.852	2.270	2.242	0.125		**Approximate**	
**average**	2.316	2.441	2.335	0.896	2.232	2.265	0.128		**IC50**	**3.83E-05 M**
**SD**	0.1446	0.1030	0.1044	0.1337	0.0692	0.0517	0.0020		**SD**	**1.56E-06**
**Inhibition rate**	-0.0236	-0.0824	-0.0327	0.5027	0.6024					

	drug concentration (M)					Positive	Negative	Drug			
**Cell Line**	**3.85E-08**	**3.86E-07**	**3.85E-06**	**3.85E-05**	**3.85E-04**	**Control**	**Control**	**Control**			
**231BR**	0.686	0.656	0.617	0.460	1.977	0.647	0.131	0.213			
	0.736	0.793	0.745	0.500	2.190	0.691	0.134	0.215			
	0.811	0.842	0.853	0.520	2.182	0.729	0.136	0.215			
	0.655	0.661	0.595	0.482	2.025	0.717	0.135	0.423			
	0.620	0.621	0.638	0.538	2.108	0.852	0.135	1.382			
	0.736	0.671	0.720	0.536	2.242	0.784	0.130		**Approximate**		
**average**	0.707	0.707	0.695	0.506	2.121	0.737	0.134		**IC50**	**1.2E-05 M**	**3.85E-06 < IC50 < 3.85E-05**
**SD**	0.0681	0.0884	0.0974	0.0311	0.1032	0.0723	0.0024		**SD**	**1.75E-06**	
**Inhibition rate**	0.0476	0.0476	0.0687	0.8621	-0.2270						

	drug concentration (M)					Positive	Negative	Drug			
**Cell Line**	**3.85E-08**	**3.86E-07**	**3.85E-06**	**3.85E-05**	**3.85E-04**	**Control**	**Control**	**Control**			
**231**	0.604	0.572	0.575	0.432	2.106	0.633	0.076	0.210			
	0.525	0.561	0.557	0.429	2.179	0.573	0.079	0.211			
	0.610	0.620	0.606	0.398	2.146	0.595	0.079	0.215			
	0.626	0.620	0.597	0.448	2.144	0.597	0.078	0.364			
	0.638	0.629	0.594	0.438	2.154	0.632	0.078	1.668			
	0.673	0.675	0.639	0.430	2.077	0.693	0.077		**Approximate**		
**average**	0.613	0.613	0.595	0.429	2.134	0.621	0.078	0.212	**IC50**	**2.89E-05 M**	**3.85E-06 < IC50 < 3.85E-05**
**SD**	0.0495	0.0414	0.0279	0.0168	0.0366	0.0424	0.0012		**SD**	**2.75E-06**	
**Inhibition rate**	0.0153	0.0150	0.0485	0.6332	-0.1056						

	drug concentration (M)					Positive	Negative	Drug		
**Cell Line**	**3.85E-08**	**3.86E-07**	**3.85E-06**	**3.85E-05**	**3.85E-04**	**Control**	**Control**	**Control**		
**MCF10A**	0.458	0.481	0.492	0.579	1.113	0.489	0.182	0.186		
	0.472	0.453	0.425	0.604	1.228	0.451	0.186	0.188		
	0.447	0.393	0.473	0.551	1.174	0.439	0.185	0.203		
	0.488	0.441	0.449	0.646	1.298	0.443	0.185	0.390		
	0.471	0.474	0.466	0.607	1.229	0.448	0.188	0.843		
	0.490	0.481	0.516	0.668	1.189	0.446	0.188		**Approximate**	
**average**	0.462	0.440	0.453	0.609	1.205	0.459	0.186		**IC50**	**> 3.85E-04 M**
**SD**	0.0119	0.0343	0.0214	0.0428	0.0623	0.0204	0.0023			
**Inhibition rate**	-0.0110	0.0687	0.0211	0.1349	-0.3266					

C9:

	drug concentration (M)					Positive	Negative	Drug			
**Cell Line**	**5.60E-08**	**5.60E-07**	**5.60E-06**	**5.60E-05**	**5.60E-04**	**Control**	**Control**	**Control**			
**HTB131**	1.738	1.635	1.942	0.282	0.374	1.498	0.078	0.143			
	1.566	1.735	1.820	0.254	0.433	2.081	0.079	0.136			
	1.817	1.977	1.784	0.378	0.439	2.311	0.079	0.128			
	1.576	1.995	1.577	0.344	0.472	1.962	0.077	0.168			
	1.502	1.642	1.641	0.296	0.488	2.232	0.078	0.418			
	1.777	1.976	1.787	0.409	0.393	1.707	0.078		**Approximate**		
**average**	1.663	1.827	1.759	0.327	0.433	1.965	0.078	0.136	**IC 50**	**1.02E-05 M**	**5.6E-06 < IC50 < 5.6E-05 M**
**deviation**	0.1306	0.1746	0.1309	0.0599	0.0440	0.3128	0.0008		**SD**	**1.87E-06**	
**Inhibition Rate**	0.1602	0.0733	0.1094	0.8849	0.9612						

	drug concentration (M)					Positive	Negative	Drug			
**Cell Line**	**5.60E-08**	**5.60E-07**	**5.60E-06**	**5.60E-05**	**5.60E-04**	**Control**	**Control**	**Control**			
**CRL**	1.763	1.837	1.589	0.220	0.457	1.860	0.125				
	1.898	2.083	1.769	0.215	0.499	1.788	0.128				
	2.078	2.253	1.974	0.254	0.441	1.983	0.129				
	2.161	2.242	1.740	0.214	0.555	2.561	0.128				
	2.276	2.305	1.352	0.214	0.534	2.268	0.129				
	2.036	2.066	1.537	0.212	0.463	2.344	0.127		**Approximate**		
**average**	2.035	2.131	1.660	0.222	0.492	2.134	0.128		**IC50**	**9.42E-06 M**	**5.6E-06 < IC50 < 5.6E-05 M**
**deviation**	0.1836	0.1735	0.2154	0.0161	0.0457	0.3040	0.0015		**SD**	**1.64E-06**	
**Inhibition Rate**	0.0492	0.0015	0.2362	0.9532	0.9634						

	drug concentration (M)					Positive	Negative	Drug			
**Cell Line**	**5.60E-08**	**5.60E-07**	**5.60E-06**	**5.60E-05**	**5.60E-04**	**Control**	**Control**	**Control**			
**231BR**	0.679	0.638	0.589	0.275	0.666	0.656	0.132				
	0.745	0.671	0.584	0.283	0.688	0.719	0.136				
	0.642	0.705	0.614	0.281	0.627	0.648	0.138				
	0.625	0.665	0.651	0.286	0.579	0.739	0.135				
	0.631	0.709	0.645	0.276	0.535	0.810	0.134				
	0.745	0.703	0.666	0.283	0.502	0.716	0.131		**Approximate**		
**average**	0.678	0.682	0.625	0.281	0.600	0.715	0.134		**IC50**	**1.72E-05 M**	**5.6E-06 < IC50 < 5.6E-05 M**
**deviation**	0.05530	0.02844	0.03422	0.00432	0.07362	0.05931	0.00258		**SD**	**1.86E-06**	
**Inhibition Rate**	0.06347	0.05658	0.15480	0.80747	0.68879						

	drug concentration (M)					Positive	Negative	Drug			
**Cell Line**	**5.60E-08**	**5.60E-07**	**5.60E-06**	**5.60E-05**	**5.60E-04**	**Control**	**Control**	**Control**			
**231**	0.646	0.584	0.531	0.176	0.563	0.633	0.077				
	0.664	0.571	0.485	0.176	0.541	0.573	0.078				
	0.587	0.515	0.481	0.175	0.479	0.595	0.078				
	0.621	0.532	0.482	0.178	0.484	0.597	0.078				
	0.599	0.553	0.491	0.176	0.493	0.632	0.078				
	0.770	0.745	0.610	0.179	0.482	0.693	0.077		**Approximate**		
**average**	0.648	0.583	0.513	0.177	0.507	0.621	0.078		**IC50**	**1.18E-05 M**	**5.6E-06 < IC50 < 5.6E-05 M**
**deviation**	0.06632	0.08308	0.05096	0.00151	0.03585	0.04243	0.00052		**SD**	**9.42E-07**	
**Inhibition Rate**	-0.05035	0.06847	0.19742	0.89196	0.74401						

	drug concentration (M)					Positive	Negative	Drug			
Cell Line	5.60E-08	5.60E-07	5.60E-06	5.60E-05	5.60E-04	Control	Control	Control			
MCF10A	0.458	0.443	0.418	0.242	0.408	0.445	0.180	0.184			
	0.474	0.464	0.425	0.249	0.426	0.466	0.185	0.181			
	0.441	0.438	0.412	0.249	0.425	0.476	0.183	0.187			
	0.434	0.446	0.402	0.252	0.438	0.455	0.182	0.240			
	0.485	0.439	0.411	0.248	0.434	0.499	0.184	0.402			
	0.487	0.443	0.396	0.244	0.412	0.474	0.177		Approximate		
average	0.463	0.446	0.411	0.247	0.424	0.469	0.182	0.184	IC50	1.14E-05 M	5.6E-06 < IC50 < 5.6E-05 M
deviation	0.02250	0.00952	0.01050	0.00367	0.01184	0.01873	0.00293		SD	1.11E-06	
Inhibition Rate	0.02033	0.08188	0.20325	0.96748	0.91696						

L[13]:

	drug concentration (M)					Positive	Negative	Drug		
Cell Line	4.20E-08	4.20E-07	4.20E-06	4.20E-05	4.20E-04	Control	Control	Control		
231BR	0.969	0.938	0.626	0.342	1.589	1.016	0.173	0.189		
	0.956	0.931	0.577	0.339	1.614	0.966	0.172	0.324		
	0.877	0.960	0.549	0.334	1.606	0.962	0.169	1.805		
average	0.934	0.943	0.584	0.338	1.603	0.981	0.171		Approximate	
deviation (SD)	0.04979	0.01513	0.03897	0.00404	0.01277	0.03009	0.00208		IC50	3.1E-06 M
Inhibition Rate	0.05802	0.04691	0.51235	0.98230	0.99630					

L[15]:

	drug concentration (M)					Positive	Negative	Drug		
Cell Line	6.45E-08	6.45E-07	6.45E-06	6.45E-05	6.45E-04	Control	Control	Control		
231BR	1.003	1.005	0.975	0.431	0.989	1.001	0.176	0.188		
	1.006	0.951	0.957	0.401	0.981	0.992	0.175	0.258		
	1.055	1.013	0.997	0.372	0.967	0.962	0.176	0.964		
average	1.021	0.990	0.976	0.401	0.979	0.985	0.176		Approximate	
deviation	0.02919	0.03372	0.02003	0.02950	0.01114	0.02042	0.00058		IC50	3.2E-05 M
Inhibition	-0.04491	-0.00577	0.02555	0.82283	0.98146					

L[10]:

	drug concentration (M)					Positive	Negative	Drug		
Cell Line	3.00E-08	3.00E-07	3.00E-06	3.00E-05	3.00E-04	Control	Control	Control		
231BR	1.026	0.987	0.973	0.960	0.632	1.029	0.179	0.171		
	1.043	1.059	0.883	1.039	0.624	1.035	0.178	0.190		
	1.063	1.053	0.988	1.068	0.628	0.997	0.176	0.287		
average	1.044	1.033	0.948	1.022	0.628	1.020	0.178		Approximate	
deviation (SD)	0.01852	0.03995	0.05679	0.05590	0.00400	0.02043	0.00153		IC50	2.85E-04 M
Iihibition Rate	-0.02847	-0.01542	0.08541	0.01265	0.59549					

C15:

	drug concentration (M)					Positive	Negative	Drug		
Cell Line	3.70E-08	3.70E-07	3.70E-06	3.70E-05	3.70E-04	Control	Control	Control		
231BR	1.051	1.025	0.929	0.599	1.451	1.030	0.179	0.174		
	1.042	0.986	0.934	0.496	1.437	1.034	0.179	0.232		
	1.041	1.020	0.882	0.424	1.300	1.071	0.181	1.177		
average	1.045	1.010	0.915	0.506	1.396	1.045	0.180		Approximate	
deviation (SD)	0.005508	0.021221	0.028688	0.087956	0.083433	0.022605	0.001155		IC50	3.19E-05 M
Inhibition Rate	0.000385	0.040077	0.150289	0.682852	0.746821					

C13: (Batch 1)

	drug concentration (M)					Positive	Negative	Drug		
Cell Line	1.60E-08	1.60E-07	1.60E-06	1.60E-05	1.60E-04	Control	Control	Control		
231BR	1.204	1.259	1.254	0.905	0.906	1.293	0.201	0.203		
	1.281	1.271	1.236	0.913	0.916	1.229	0.204	0.243		
	1.263	1.217	1.205	0.898	0.962	1.292	0.201	1.092		
average	1.249	1.249	1.232	0.905	0.928	1.271	0.202		Approximate	
deviation (SD)	0.04028	0.02835	0.02479	0.00751	0.02987	0.03667	0.00173		IC50	2.78E-05 M
Inhibition Rate	0.02027	0.02058	0.03679	0.19052	0.96352					

C13: (Batch 2)

	drug concentration (M)					Positive	Negative	Drug		
Cell Line	1.65E-08	1.65E-07	1.65E-06	1.65E-05	1.65E-04	Control	Control	Control		
231BR	1.238	1.239	1.124	0.340	1.396	1.219	0.197	0.212		
	1.263	1.285	1.224	0.354	1.366	1.257	0.202	0.282		
	1.161	1.293	1.214	0.339	1.210	1.243	0.196	1.438		
average	1.221	1.272	1.187	0.344	1.324	1.240	0.198		Approximate	
deviation (SD)	0.05316	0.02914	0.05508	0.00839	0.09986	0.01922	0.00321		IC50	1.12E-05
Inhibition Rate	0.01855	-0.03103	0.05054	0.75016	0.83877					

C8:

	drug concentration (M)					Positive	Negative	Drug		
Cell Line	2.50E-08	2.50E-07	2.50E-06	2.50E-05	2.50E-04	Control	Control	Control		
231BR	1.296	1.219	1.095	0.878	0.499	1.255	0.196	0.191		
	1.297	1.223	1.089	0.867	0.510	1.230	0.198	0.198		
	1.246	1.239	1.089	0.838	0.509	1.222	0.198	0.453		
average	1.280	1.227	1.091	0.861	0.506	1.236	0.197		Approximate	
deviation (SD)	0.02916	0.01058	0.00346	0.02066	0.00608	0.01721	0.00115		IC50	4.85E-05 M
Inhibition Rate	-0.04203	0.00866	0.13956	0.36092	0.75938					

C10:

	drug concentration (M)					Positive	Negative	Drug		
**Cell Line**	**4.80E-08**	**4.80E-07**	**4.80E-06**	**4.80E-05**	**4.80E-04**	**Control**	**Control**	**Control**		
**231BR**	1.256	1.264	0.322	0.237	1.935	1.222	0.197	0.189		
	1.255	1.260	0.322	0.237	1.844	1.234	0.196	0.204		
	1.219	1.226	0.324	0.244	1.853	1.284	0.197	2.278	**Approximate**	
**average**	1.243	1.250	0.323	0.239	1.877	1.247	0.197		**IC50**	**1.2E-06 M**
**deviation (SD)**	0.02108	0.02088	0.00115	0.00404	0.05014	0.03288	0.00058			
**Inhibition Rate**	0.00349	-0.00286	0.88032	0.96635	1.18730					

L[7]:

	drug concentration (M)					Positive	Negative		
**Cell Line**	**7.50E-10**	**7.50E-09**	**7.50E-08**	**7.50E-07**	**7.50E-06**	**Control**	**Control**		
**231BR**	1.030	0.941	0.486	0.383	0.363	0.902	0.053		
	1.028	0.955	0.513	0.391	0.358	0.935	0.102		
	1.035	0.958	0.536	0.407	0.365	1.000	0.101		
	1.064	0.966	0.520	0.429	0.370	0.970	0.100		
	1.020	0.928	0.548	0.422	0.370	0.943	0.098		
	1.045	1.034	0.563	0.424	0.372	1.005	0.054		
**average**	1.035	0.955	0.529	0.411	0.367	0.962	0.100	**Approximate**	
**deviation (SD)**	0.007594	0.010424	0.015777	0.015341	0.003559	0.029428	0.001708	**IC50**	**7.5E-08 M**
**inhibition rate**	1.084131	0.991877	0.497824	0.360603	0.309545			**SD**	**3.78E-09**

	drug concentration (M)					Positive	Negative		
**Cell Line**	**7.50E-10**	**7.50E-09**	**7.50E-08**	**7.50E-07**	**7.50E-06**	**Control**	**Control**		
**361**	1.312	1.176	1.207	1.224	0.294	0.791	0.139		
	1.012	1.146	1.228	1.002	0.278	1.027	0.132		
	0.874	1.058	1.127	1.202	0.264	1.191	0.134		
	1.255	1.138	1.221	1.298	0.269	1.155	0.200		
	1.309	1.095	1.076	1.204	0.249	1.158	0.146		
	1.149	1.267	1.063	1.234	0.295	1.301	0.202		
**average**	1.181	1.139	1.158	1.216	0.276	1.133	0.155	**Approximate**	
**deviation (SD)**	0.1310	0.0334	0.0684	0.0156	0.0132	0.0724	0.0306	**IC50**	**7.5E-07 < IC50 < 7.5E-06 M**
**inhibition rate**	1.0496	1.0061	1.0256	1.0851	0.1242				

	drug concentration (M)					Positive	Negative		
**Cell Line**	**7.50E-10**	**7.50E-09**	**7.50E-08**	**7.50E-07**	**7.50E-06**	**Control**	**Control**		
**CRL**	2.695	2.614	2.435	1.400	0.269	2.498	0.212		
	2.704	2.568	2.403	1.415	0.342	2.387	0.218		
	2.597	2.510	2.281	1.385	0.340	2.349	0.215		
	2.605	2.560	2.425	1.448	0.356	2.385	0.215		
	2.598	2.565	2.427	1.394	0.312	2.396	0.214		
	2.728	2.641	2.441	1.386	0.285	2.432	0.213		
**average**	2.651	2.577	2.423	1.399	0.320	2.400	0.214	**Approximate**	
**deviation (SD)**	0.05677	0.02505	0.01370	0.01226	0.02691	0.02186	0.00096	**IC50**	**7.5E-07 < IC50 < 7.5E-06 M**
**inhibition rate**	1.11461	1.08086	1.01029	0.54192	0.04827				

	drug concentration (M)					Positive	Negative		
**Cell Line**	**7.50E-10**	**7.50E-09**	**7.50E-08**	**7.50E-07**	**7.50E-06**	**Control**	**Control**		
**HTB131**	1.463	1.143	0.695	0.546	0.264	1.125	0.139		
	1.280	1.212	0.858	0.665	0.290	1.222	0.144		
	1.145	1.317	0.958	0.536	0.262	1.169	0.138		
	1.093	1.110	0.707	0.607	0.287	1.109	0.137		
	1.207	1.205	0.798	0.573	0.241	1.143	0.134		
	1.230	1.173	0.686	0.534	0.221	0.947	0.132		
**average**	1.216	1.183	0.765	0.566	0.264	1.137	0.137	**Approximate**	
**deviation (SD)**	0.05601	0.03175	0.07746	0.03178	0.01881	0.02574	0.00216	**IC50**	**7.5E-08 < IC50 < 7.5E-07 M**
**inhibition rate**	1.07904	1.04677	0.62781	0.42871	0.12656				

	drug concentration (M)					Positive	Negative		
**Cell Line**	**7.50E-10**	**7.50E-09**	**7.50E-08**	**7.50E-07**	**7.50E-06**	**Control**	**Control**		
**231**	0.732	0.712	0.444	0.401	0.274	0.737	0.170		
	0.696	0.756	0.464	0.381	0.276	0.720	0.166		
	0.778	0.715	0.465	0.419	0.284	0.757	0.182		
	0.733	0.696	0.493	0.372	0.258	0.708	0.170		
	0.747	0.715	0.457	0.387	0.279	0.660	0.168		
	0.723	0.552	0.444	0.375	0.278	0.717	0.166		
**average**	0.734	0.710	0.458	0.386	0.277	0.721	0.169	**Approximate**	
**deviation (SD)**	0.00991	0.00911	0.00968	0.01114	0.00222	0.01212	0.00191	**IC50**	**7.5E-08 < IC50 < 7.5E-07 M**
**inhibition rate**	1.02400	0.98007	0.52355	0.39402	0.19611				

	drug concentration (M)					Positive	Negative		
**Cell Line**	**7.50E-10**	**7.50E-09**	**7.50E-08**	**7.50E-07**	**7.50E-06**	**Control**	**Control**		
**HTB22**	0.703	0.670	0.628	0.572	0.324	0.671	0.152		
	0.692	0.668	0.631	0.555	0.320	0.679	0.156		
	0.662	0.634	0.620	0.559	0.316	0.652	0.156		
	0.665	0.653	0.645	0.562	0.328	0.695	0.154		
	0.667	0.642	0.610	0.558	0.310	0.669	0.155		
	0.596	0.539	0.565	0.524	0.249	0.631	0.153		
**average**	0.672	0.649	0.622	0.559	0.318	0.668	0.155	**Approximate**	
**deviation (SD)**	0.01382	0.01473	0.00939	0.00289	0.00597	0.01135	0.00129	**IC50**	**7.5E-07 < IC50 < 7.5E-06 M**
**inhibition rate**	1.00731	0.96396	0.91135	0.78714	0.31758				

	drug concentration (M)					Positive	Negative		
**Cell Line**	**7.50E-10**	**7.50E-09**	**7.50E-08**	**7.50E-07**	**7.50E-06**	**Control**	**Control**		
**MCF10A-Core**	1.239	1.139	0.776	0.653	0.351	1.140	0.182		
	1.267	1.167	0.825	0.684	0.361	1.141	0.189		
	1.299	1.238	0.832	0.703	0.366	1.255	0.190		
	1.267	1.159	0.733	0.638	0.342	1.237	0.191		
	1.256	1.130	0.745	0.649	0.353	1.151	0.192		
	1.316	1.126	0.719	0.623	0.334	1.133	0.240		
**average**	1.272	1.149	0.770	0.656	0.352	1.196	0.191	**Approximate**	
**deviation (SD)**	0.01857	0.01717	0.04105	0.01971	0.00780	0.05834	0.00129	**IC50**	**7.5E-08 < IC50 < 7.5E-07 M**
**inhibition rate**	1.07583	0.95301	0.57608	0.46295	0.11114				

Ps:

	drug concentration (M)						Positive	Negative	Drug		
**cell line**	**7.5xE-10**	**7.5xE-9**	**7.5xE-8**	**7.5xE-7**	**7.5xE-6**	**7.5xE-5**	**Control**	**Control**	**Control**		
**231BR**	0.98	0.825	0.78	0.456	0.411		1.162	0.119			
	0.999	0.976	0.915	0.756	0.413		0.927	0.126			
	1.024	0.969	0.963	0.795	0.403		0.93	0.123			
	0.821	0.869	0.87	0.76	0.413		0.943	0.12			
	0.979	0.979	0.945	0.768	0.391		0.918	0.123			
	1.077	1.039	0.942	0.842	0.413		0.98	0.245		**Approximate**	
**average**	0.9373	0.9483	0.9413	0.7698	0.4100		0.9450	0.1230		**IC50**	**7.5E-07 < IC50 < 7.5E-06 M**
**deviation**	0.0211	0.0530	0.0198	0.0176	0.0048		0.0243	0.0024			
**inhibition rate**	0.9907	1.0040	0.9954	0.7868	0.3491						

	drug concentration (M)						Positive	Negative	Drug		
**cell line**	**7.5xE-10**	**7.5xE-9**	**7.5xE-8**	**7.5xE-7**	**7.5xE-6**	**7.5xE-5**	**Control**	**Control**	**Control**		
**361**	1.362	0.985	1.183	1.238	1.167		1.25	0.138			
	1.408	1.181	1.054	1.111	1.231		1.24	0.139			
	1.334	1.213	1.095	1.18	1.323		1.253	0.139			
	1.35	1.264	1.192				1.118	0.138			
	1.466	0.982	1.182				1.143	0.133			
	1.438	1.332	1.26				1.142	0.137		**Approximate**	
**average**	1.3895	1.1608	1.1953	1.1763	1.2403		1.1938	0.1380		**IC50**	**> 7.5E-06 M**
**deviation**	0.0409	0.1221	0.0456	0.0636	0.0784		0.0593	0.0008			
**inhibition rate**	1.1854	0.9687	1.0015	0.9835	1.0441						

	drug concentration (M)						Positive	Negative	Drug		
**cell line**	**7.5xE-10**	**7.5xE-9**	**7.5xE-8**	**7.5xE-7**	**7.5xE-6**	**7.5xE-5**	**Control**	**Control**	**Control**		
**CRL**		2.644	2.631	2.393	1.187	0.582	2.496	0.213			
		2.642	2.595	2.31	1.265	0.619	2.433	0.215			
		2.512	2.542	2.324	1.225	0.6	2.277	0.213			
		2.51	2.631	2.36	1.317	0.649	2.562	0.22			
		2.58	2.464	2.303	1.256	0.671	2.507	0.214			
		2.593	2.556	2.264	1.16	0.562	2.499	0.213		**Approximate**	
**average**		2.58175	2.581	2.32425	1.23325	0.6125	2.48375	0.21375		**IC50**	**7.5E-07 < IC50 < 7.5E-06 M**
**deviation**		0.0536	0.0402	0.0254	0.0353	0.0286	0.0342	0.0010			
**inhibition rate**		1.0432	1.0428	0.9297	0.4491	0.1757					

	drug concentration (M)						Positive	Negative	Drug		
**cell line**	**7.5xE-10**	**7.5xE-9**	**7.5xE-8**	**7.5xE-7**	**7.5xE-6**	**7.5xE-5**	**Control**	**Control**	**Control**		
**HTB131**		1.297	1.202	0.872	0.465	0.381	1.18	0.146			
		1.117	1.222	1.051	0.558	0.432	1.288	0.147			
		1.21	1.286	1.04	0.51	0.414	1.251	0.148			
		1.264	1.307	1.034	0.415	0.468	1.309	0.145			
		1.192	1.067	0.949	0.705	0.459	1.312	0.146			
		1.263	1.172	0.976	0.513	0.435	0.972	0.149		**Approximate**	
**average**		1.23225	1.2205	0.99975	0.5115	0.435	1.257	0.14675		**IC50**	**7.5E-07 < IC50 < 7.5E-06 M**
**deviation**		0.0368	0.0483	0.0445	0.0380	0.0185	0.0567	0.0010			
**inhibition rate**		0.9777	0.9671	0.7683	0.3285	0.2596					

	drug concentration (M)						Positive	Negative	Drug		
**cell line**	**7.5xE-10**	**7.5xE-9**	**7.5xE-8**	**7.5xE-7**	**7.5xE-6**	**7.5xE-5**	**Control**	**Control**	**Control**		
**231**		0.694	0.696	0.599	0.383	0.236	0.784	0.183	0.16		
		0.681	0.73	0.595	0.398	0.262	0.763	0.175	0.159		
		0.699	0.692	0.642	0.358	0.257	0.669	0.178	0.161		
		0.711	0.751	0.622	0.374	0.254	0.745	0.178	0.163		
		0.65	0.626	0.581	0.354	0.235	0.709	0.169	0.177		
		0.674	0.503	0.438	0.317	0.236	0.696	0.181		**Approximate**	
**average**		0.687	0.686	0.59925	0.36725	0.24575	0.72825	0.178		**IC50**	**7.5E-07 < IC50 < 7.5E-06 M**
**deviation**		0.0115	0.0435	0.0170	0.0136	0.0113	0.0311	0.0024			
**inhibition rate**		0.9250	0.9232	0.7656	0.3439	0.1231					

Taxol (Paclitaxel):

	drug concentration (M)					Positive	Negative		
**Cell Line**	**7.50E-10**	**7.50E-09**	**7.50E-08**	**7.50E-07**	**7.50E-06**	**Control**	**Control**		
**HTB131**	1.559	0.96	1.15	0.365	0.703	1.587	0.148		
	1.869	0.98	0.873	0.512	0.363	1.744	0.148		
	1.727	1.277	1.17	0.591	0.593	1.521	0.145		
	1.704	1.152	1.088	0.539	0.778	1.827	0.146		
	1.742	1.053	1.017	0.522	0.899	1.818	0.149		
	1.794	1	1.06	0.507	0.856	1.841	0.149		
**average**	1.733	1.070	1.060	0.506	0.699	1.723	0.148	**IC50**	**5.7E-08 M**
**SD**	0.103	0.122	0.108	0.075	0.198	0.137	0.002	**SD**	**8.32E-09**
**inhibition rate**	-0.006	0.414	0.421	0.772	0.650				

	drug concentration (M)					Positive	Negative		
**Cell Line**	**7.50E-10**	**7.50E-09**	**7.50E-08**	**7.50E-07**	**7.50E-06**	**Control**	**Control**		
**CRL**	1.158	0.812	0.657	0.497	0.386	1.579	0.19		
	1.378	0.601	0.571	0.566	0.573	1.572	0.185		
	1.38	1.03	0.651	0.513	0.545	1.633	0.189		
	1.602	0.695	0.48	0.609	0.484	1.565	0.188		
	1.578	0.801	0.696	0.67	0.605	1.626	0.187		
	1.713	0.996	0.705	0.497	0.522	1.61	0.188		
**average**	1.468	0.823	0.627	0.559	0.519	1.598	0.188	**IC50**	**2.8E-08 M**
**SD**	0.201	0.167	0.086	0.070	0.077	0.029	0.002	**SD**	**5.51E-09**
**inhibition rate**	0.092	0.550	0.689	0.737	0.765				

	drug concentration (M)					Positive	Negative		
**Cell Line**	**7.50E-10**	**7.50E-09**	**7.50E-08**	**7.50E-07**	**7.50E-06**	**Control**	**Control**		
**231BR**	0.641	0.577	0.452	0.449	0.423	0.654	0.256		
	0.606	0.556	0.502	0.483	0.493	0.822	0.258		
	0.605	0.607	0.52	0.458	0.43	0.722	0.256		
	0.685	0.604	0.531	0.503	0.459	0.762	0.249		
	0.726	0.582	0.53	0.479	0.448	0.786	0.262		
	0.835	0.605	0.562	0.522	0.441	0.935	0.288		
**average**	0.683	0.589	0.516	0.482	0.449	0.780	0.262	**IC50**	**3.9E-08 M**
**SD**	0.080	0.019	0.034	0.025	0.023	0.087	0.012	**SD**	**8.62E-09**
**inhibition rate**	0.187	0.370	0.509	0.575	0.639				

	drug concentration (M)					Positive	Negative		
**Cell Line**	**7.50E-10**	**7.50E-09**	**7.50E-08**	**7.50E-07**	**7.50E-06**	**Control**	**Control**		
**231**	0.703	0.492	0.399	0.391	0.363	0.747	0.211		
	0.624	0.455	0.37	0.379	0.363	0.723	0.212		
	0.615	0.448	0.369	0.347	0.344	0.679	0.213		
	0.661	0.472	0.371	0.349	0.358	0.717	0.238		
	0.65	0.469	0.36	0.352	0.361	0.707	0.218		
	0.652	0.57	0.358	0.304	0.401	0.762	0.223		
**average**	0.651	0.484	0.371	0.354	0.365	0.723	0.219	**IC50**	**2.13E-08 M**
**SD**	0.031	0.045	0.015	0.030	0.019	0.029	0.010	**SD**	**2.83E-09**
**inhibition rate**	0.142	0.473	0.698	0.733	0.710				

	drug concentration (M)					Positive	Negative		
**Cell Line**	**7.50E-10**	**7.50E-09**	**7.50E-08**	**7.50E-07**	**7.50E-06**	**Control**	**Control**		
**MCF10A**	0.425	0.37	0.356	0.35	0.313	0.45	0.179		
	0.424	0.44	0.367	0.354	0.353	0.464	0.184		
	0.405	0.367	0.368	0.351	0.346	0.426	0.178		
	0.411	0.379	0.349	0.337	0.35	0.419	0.181		
	0.425	0.374	0.363	0.334	0.346	0.442	0.182		
	0.411	0.369	0.362	0.332	0.333	0.469	0.179		
**average**	0.417	0.383	0.361	0.343	0.340	0.445	0.181	**IC50**	**3.0E-05 M**
**SD**	0.009	0.028	0.007	0.010	0.015	0.020	0.002	**SD**	**2.61E-06**
**inhibition rate**	0.107	0.234	0.319	0.386	0.397				

	drug concentration (M)					Positive	Negative		
**Cell Line**	**7.50E-10**	**7.50E-09**	**7.50E-08**	**7.50E-07**	**7.50E-06**	**Control**	**Control**		
**361**	3.247	3.147	2.67	2.501	2.601	3.618	0.156		
	3.159	2.865	2.36	2.443	2.49	3.27	0.158		
	3.074	3.033	2.444	2.399	2.693	3.416	0.159		
	3.378	3.239	2.44	2.455	2.506	3.269	0.152		
	3.593	3.137	2.437	2.444	2.335	3.33	0.156		
	3.3	3.039	2.567	2.312	2.221	3.264	0.156		
**average**	3.292	3.077	2.486	2.426	2.474	3.361	0.156	**IC50**	**> 7.5E-06 M**
**SD**	0.182	0.129	0.112	0.065	0.172	0.139	0.002		
**inhibition rate**	0.022	0.089	0.273	0.292	0.277				

	drug concentration (M)						Positive	Negative	Drug		
**cell line**	**7.5xE-10**	**7.5xE-9**	**7.5xE-8**	**7.5xE-7**	**7.5xE-6**	**7.5xE-5**	**Control**	**Control**	**Control**		
**HTB22**		0.753	0.672	0.682	0.516	0.739	0.676	0.125			
		0.689	0.666	0.648	0.495	0.709	0.648	0.157			
		0.671	0.672	0.652	0.503	0.702	0.696	0.157			
		0.697	0.674	0.645	0.492	0.711	0.704	0.155			
		0.684	0.663	0.642	0.498	0.711	0.653	0.154			
		0.688	0.664	0.654	0.514	0.757	0.637	0.154		**Approximate**	
**average**		0.6895	0.6685	0.64975	0.5025	0.70825	0.66825	0.155		**IC50**	**> 7.5E-05 M**
**deviation**		0.0083	0.0041	0.0040	0.0083	0.0043	0.0222	0.0014			
**inhibition rate**		1.0414	1.0005	0.9640	0.6771	0.5390					

	drug concentration (M)						Positive	Negative	Drug		
**cell line**	**7.5xE-10**	**7.5xE-9**	**7.5xE-8**	**7.5xE-7**	**7.5xE-6**	**7.5xE-5**	**Control**	**Control**	**Control**		
**MCF10A**		1.153	1.255	1.061	0.673	0.512	1.2	0.183			
		1.237	1.233	1.069	0.709	0.5	1.264	0.19			
		1.205	1.211	1.012	0.708	0.488	1.228	0.191			
		1.189	1.227	1.107	0.744	0.501	1.237	0.19			
		1.239	1.258	1.062	0.705	0.521	1.209	0.188			
		1.341	1.245	1.122	0.735	0.519	1.293			**Approximate**	
**average**		1.2175	1.24	1.07475	0.71425	0.508	1.2345	0.18975		**IC50**	**7.5E-06 M**
**deviation**		0.0246	0.0125	0.0218	0.0139	0.0091	0.0229	0.0013		**SD**	**2.59E-07**
**inhibition rate**		0.9837	1.0053	0.8471	0.5020	0.3046					

Table S4|Secondary cytotoxicity tests of **L[13]**, **C10**, **L[7]**, **C1**, and **Ps**, respectively, on different cancer cell lines.

L[13]:

	drug concentration (M)								Positive	Negative	Drug		
**Cell Line**	**3.90E-07**	**7.80E-07**	**1.56E-06**	**3.13E-06**	**6.25E-06**	**1.25E-05**	**2.10E-05**	**4.20E-05**	**Control**	**Control**	**Control**		
**231BR**	0.602	0.655	0.611	0.540	0.423	0.292	0.274	0.341	0.666	0.173	0.203		
	0.629	0.623	0.601	0.546	0.429	0.285	0.271	0.348	0.662	0.172	0.208		
	0.589	0.705	0.630	0.585	0.439	0.309	0.275	0.347	0.641	0.169	0.214		
	0.637	0.646	0.570	0.533	0.431	0.281	0.276	0.346	0.647	0.173	0.231		
	0.698	0.650	0.597	0.547	0.441	0.307	0.274	0.350	0.701	0.168	0.260		
	0.722	0.632	0.667	0.551	0.424	0.315	0.281	0.380	0.753	0.171	0.286		
**average**	0.6462	0.6518	0.6127	0.5503	0.4312	0.2982	0.2752	0.3520	0.6783	0.1710		**IC50**	**5.06E-06 M**
**deviation (SD)**	0.05298	0.02863	0.03304	0.01811	0.00749	0.01403	0.00331	0.01404	0.04215	0.00210		**SD**	**4.47E-07**
**Inhibition Rate**	0.06702	0.05509	0.13754	0.27930	0.54281	0.85860	0.96807	0.86105					

	drug concentration (M)								Positive	Negative	Drug		
**Cell Line**	**3.90E-07**	**7.80E-07**	**1.56E-06**	**3.13E-06**	**6.25E-06**	**1.25E-05**	**2.10E-05**	**4.20E-05**	**Control**	**Control**	**Control**		
**CRL**	1.833	1.647	1.629	1.679	0.949	0.270	0.217	0.338	1.806	0.181	0.189		
	1.705	1.674	1.754	1.715	1.470	0.333	0.207	0.348	1.719	0.179	0.195		
	1.728	1.736	1.787	1.705	1.592	0.321	0.219	0.348	1.685	0.181	0.199		
	1.658	1.655	1.799	1.604	1.309	0.329	0.218	0.334	1.716	0.184	0.214		
	1.679	1.661	1.729	1.661	1.426	0.364	0.218	0.347	1.787	0.182	0.243		
	1.790	1.588	1.622	1.370	1.308	0.328	0.216	0.342	1.810	0.178	0.300		
**average**	1.712	1.660	1.720	1.622	1.342	0.324	0.216	0.343	1.754	0.181	0.194		
**deviation (SD)**	0.05098	0.04765	0.07727	0.12972	0.22029	0.03049	0.00445	0.00588	0.05359	0.00214	0.00503	**IC50**	**6.53E-06 M**
**Inhibition Rate**	0.02692	0.06015	0.02179	0.08440	0.26389	0.91656	0.98600	0.97254				**SD**	**9.47E-07**

	drug concentration (M)								Positive	Negative	Drug		
**Cell Line**	**3.90E-07**	**7.80E-07**	**1.56E-06**	**3.13E-06**	**6.25E-06**	**1.25E-05**	**2.10E-05**	**4.20E-05**	**Control**	**Control**	**Control**		
**HTB131**	1.988	1.834	1.708	1.414	1.350	1.091	0.340	0.236	1.650	0.138	0.140		
	1.822	1.650	1.563	1.634	1.379	1.129	0.306	0.237	1.587	0.137	0.140		
	1.745	1.713	1.609	1.655	1.214	1.100	0.340	0.231	1.601	0.141	0.147		
**average**	1.852	1.732	1.627	1.568	1.314	1.107	0.329	0.235	1.613	0.139	0.142		
**deviation (SD)**	0.12419	0.09351	0.07410	0.13349	0.08809	0.01986	0.01963	0.00321	0.03308	0.00208	0.00404	**IC50**	**1.26E-05 M**
**Inhibition Rate**	-0.16225	-0.08112	-0.00929	0.03082	0.20304	0.34421	0.87310	0.93700				**SD**	**3.84E-07**

	drug concentration (M)								Positive	Negative	Drug		
**Cell Line**	**3.90E-07**	**7.80E-07**	**1.56E-06**	**3.13E-06**	**6.25E-06**	**1.25E-05**	**2.10E-05**	**4.20E-05**	**Control**	**Control**	**Control**		
**231**	0.635	0.619	0.593	0.528	0.385	0.251	0.194	0.248	0.686	0.168	0.170		
	0.647	0.626	0.574	0.534	0.410	0.244	0.201	0.248	0.699	0.165	0.197		
	0.660	0.600	0.587	0.530	0.332	0.229	0.199	0.252	0.714	0.171	0.239		
**average**	0.647	0.615	0.585	0.531	0.376	0.241	0.198	0.249	0.700	0.168	0.170		
**deviation (SD)**	0.01250	0.01345	0.00971	0.00306	0.03983	0.01124	0.00361	0.00231	0.01401	0.00300		**IC50**	**3.49E-06 M**
**Inhibition Rate**	0.09937	0.16038	0.21761	0.31950	0.61195	0.86541	0.99811	0.98050				**SD**	**1.03E-07**

	drug concentration (M)								Positive	Negative	Drug		
**Cell Line**	**3.90E-07**	**7.80E-07**	**1.56E-06**	**3.13E-06**	**6.25E-06**	**1.25E-05**	**2.10E-05**	**4.20E-05**	**Control**	**Control**	**Control**		
**MCF10A**	1.198	1.156	0.569	0.328	1.232	1.186	0.197	0.209					
	1.171	1.118	0.615	0.334	1.302	1.171	0.197	0.321					
	1.174	1.056	0.608	0.323	1.301	1.215	0.199	1.323					
**average**	1.181	1.110	0.597	0.328	1.278	1.191	0.198						
**deviation (SD)**	0.01480	0.05048	0.02479	0.00551	0.04013	0.02237	0.00115					**IC50**	**3.7E-06 M**
**Inhibition Rate**	0.01018	0.08248	0.60455	0.87848	-0.08893							**SD**	**2.45E-07**

C10:

	drug concentration (M)								Positive	Negative	Drug		
**Cell Line**	**3.75E-07**	**7.50E-07**	**1.50E-06**	**3.00E-06**	**6.00E-06**	**1.20E-05**	**2.40E-05**	**4.80E-05**	**Control**	**Control**	**Control**		
**231BR**	0.684	0.644	0.469	0.360	0.241	0.199	0.204	0.218	0.671	0.197	0.189		
	0.667	0.614	0.449	0.359	0.213	0.199	0.203	0.206	0.685	0.196	0.192		
	0.674	0.655	0.442	0.359	0.230	0.200	0.202	0.210	0.698	0.196	0.178		
	0.691	0.651	0.475	0.355	0.234	0.203	0.208	0.213	0.683	0.197	0.184		
	0.654	0.692	0.450	0.371	0.230	0.203	0.209	0.216	0.675	0.196	0.188		
	0.700	0.696	0.454	0.377	0.245	0.211	0.202	0.216	0.686	0.198	0.209		
**average**	0.678	0.659	0.457	0.364	0.232	0.203	0.205	0.213	0.683	0.197	0.186		
**deviation (SD)**	0.01674	0.03094	0.01276	0.00853	0.01116	0.00455	0.00308	0.00449	0.00944	0.00082		**IC50**	**2.20E-06 M**
**Inhibition Rate**	0.00939	0.04896	0.45573	0.64286	0.90711	0.96680	0.96244	0.99162				**SD**	**1.16E-07**

	drug concentration (M)								Positive	Negative		
**Cell Line**	**3.75E-07**	**7.50E-07**	**1.50E-06**	**3.00E-06**	**6.00E-06**	**1.20E-05**	**2.40E-05**	**4.80E-05**	**Control**	**Control**		
**CRL**	1.839	1.251	0.972	0.569	0.202	0.177	0.185	0.190	1.833	0.177		
	1.847	1.493	0.931	0.563	0.212	0.186	0.183	0.189	1.864	0.176		
	1.675	1.403	0.944	0.519	0.212	0.181	0.184	0.192	1.694	0.172		
	1.765	1.544	0.972	0.535	0.215	0.195	0.183	0.205	1.789	0.174		
	1.768	1.434	0.961	0.568	0.210	0.208	0.187	0.200	1.765	0.169		
	1.885	1.656	1.010	0.651	0.219	0.198	0.183	0.199	1.849	0.182		
**average**	1.797	1.464	0.965	0.568	0.212	0.191	0.184	0.196	1.799	0.175		
**deviation (SD)**	0.07584	0.13718	0.02733	0.04561	0.00568	0.01162	0.00160	0.00643	0.06350	0.00447	**IC50**	**1.60E-06 M**
**Inhibition Rate**	0.00154	0.20659	0.51355	0.75831	0.97742	0.99025	0.99436	0.98717			**SD**	**8.42E-08**

	drug concentration (M)								Positive	Negative		
**Cell Line**	**3.75E-07**	**7.50E-07**	**1.50E-06**	**3.00E-06**	**6.00E-06**	**1.20E-05**	**2.40E-05**	**4.80E-05**	**Control**	**Control**		
**HTB131**	1.534	1.577	0.653	0.661	0.446	0.124	0.118	0.132	1.493	0.102		
	1.536	1.493	0.656	0.600	0.435	0.132	0.118	0.134	1.588	0.098		
	1.578	1.491	0.676	0.638	0.439	0.122	0.120	0.137	1.569	0.118		
**average**	1.549	1.520	0.662	0.633	0.440	0.126	0.119	0.134	1.550	0.106		
**deviation (SD)**	0.02485	0.04908	0.01250	0.03081	0.00557	0.00529	0.00115	0.00252	0.05027	0.01058	**IC50**	**2.17E-06 M**
**Inhibition Rate**	0.00046	0.02054	0.61519	0.63504	0.76870	0.98615	0.99123	0.98869			**SD**	**1.00E-07**

	drug concentration (M)								Positive	Negative		
**Cell Line**	**3.75E-07**	**7.50E-07**	**1.50E-06**	**3.00E-06**	**6.00E-06**	**1.20E-05**	**2.40E-05**	**4.80E-05**	**Control**	**Control**		
**231**	0.640	0.679	0.597	0.438	0.242	0.132	0.116	0.121	0.680	0.107		
	0.631	0.649	0.601	0.440	0.237	0.130	0.111	0.120	0.730	0.101		
	0.675	0.685	0.615	0.466	0.264	0.135	0.115	0.128	0.748	0.119		
**average**	0.649	0.671	0.604	0.448	0.248	0.132	0.114	0.123	0.719	0.104		
**deviation (SD)**	0.02325	0.01929	0.00945	0.01562	0.01436	0.00252	0.00265	0.00436	0.03523	0.00424	**IC50**	**2.86E-06 M**
**Inhibition Rate**	0.11436	0.07805	0.18645	0.44065	0.76640	0.95393	0.98374	0.99350			**SD**	**2.13E-07**

	drug concentration (M)					Positive	Negative	Drug		
**Cell Line**	**4.80E-08**	**4.80E-07**	**4.80E-06**	**4.80E-05**	**4.80E-04**	**Control**	**Control**	**Control**		
**MCF10A**	1.143	1.096	0.216	0.202	1.904	1.153	0.181	0.180		
	1.164	1.192	0.217	0.197	1.803	1.173	0.183	0.193		
	1.196	1.263	0.220	0.197	1.813	1.283	0.183	1.486		
**average**	1.168	1.184	0.218	0.199	1.840	1.203	0.182			
**deviation (SD)**	0.02669	0.08381	0.00208	0.00289	0.05565	0.07000	0.00115		**IC50**	**1.4E-06 M**
**Inhibition Rate**	0.03736	0.02177	0.96329	0.98181	0.65497				**SD**	**1.51E-07**

L^7:

	drug concentration (M)								Positive	Negative			
**Cell Line**	**2.93E-07**	**5.86E-07**	**1.17E-06**	**2.34E-06**	**4.69E-06**	**9.38E-06**	**1.88E-05**	**3.75E-05**	**Control**	**Control**			
**361**	0.121	0.124	0.129	0.137	0.150	0.183	0.286	0.410					
	1.622	1.592	1.517	1.379	1.036	0.415	0.347	0.488	1.492	0.136			
	1.625	1.572	1.563	1.647	0.950	0.283	0.431	0.530	1.453	0.146			
	1.714	1.593	1.498	1.697	1.302	0.421	0.457	0.547	1.531	0.138			
	1.615	1.653	1.608	1.804	1.219	0.388	0.426	0.468	1.570	0.149			
	1.706	1.743	1.697	1.879	0.138	0.281	0.335	0.416	1.653	0.154			
**average**	1.621	1.613	1.563	1.574	1.186	0.408	0.438	0.495	1.531	0.144			
**deviation (SD)**	0.005132	0.034933	0.045501	0.080206	0.136097	0.017578	0.016643	0.031644	0.03900	0.00569	**IC50**	**5.79*10^-6 M**	**(2124.93 ng/mL)**
**survival rate**	1.0815	1.0736	1.0339	1.0365	0.7469	0.1623	0.1096	0.0615			**SD**	**3.20E-07**	
**inhibition rate**	-0.08149	-0.07356	-0.03389	-0.03654	0.25313	0.83774	0.89038	0.93846					

	drug concentration (M)								Positive	Negative			
**Cell Line**	**5.86E-09**	**1.17E-08**	**2.34E-08**	**4.69E-08**	**9.38E-08**	**1.88E-07**	**3.75E-07**	**7.50E-07**	**Control**	**Control**			
**231BR**	1.361	1.329	1.207	0.903	0.622	0.564	0.553	0.537	1.324	0.208			
	1.351	1.292	1.219	0.951	0.641	0.582	0.565	0.556	1.375	0.212			
	1.340	1.282	1.294	1.015	0.645	0.602	0.585	0.553	1.322	0.208			
	1.349	1.295	1.273	0.966	0.664	0.571	0.592	0.545	1.355	0.215			
	1.401	1.310	1.235	0.951	0.644	0.577	0.602	0.554	1.320	0.209			
	1.395	1.371	1.275	0.937	0.618	0.568	0.577	0.545	1.372	0.214			
**average**	1.364	1.307	1.251	0.951	0.638	0.575	0.580	0.549	1.343	0.211			
**deviation (SD)**	0.02132	0.01694	0.02792	0.01184	0.01080	0.00624	0.01159	0.00492	0.02440	0.00275	**IC50**	**4.63*10^-8 M**	**(16.992 ng/mL)**
**inhibition rate**	-0.01832	0.03245	0.08190	0.34614	0.62274	0.67881	0.67417	0.70110			**SD**	**6.50E-10**	

	drug concentration (M)								Positive	Negative			
**Cell Line**	**5.86E-08**	**1.17E-07**	**2.34E-07**	**4.69E-07**	**9.38E-07**	**1.88E-06**	**3.75E-06**	**7.50E-06**	**Control**	**Control**			
**CRL**	1.327	1.174	0.966	0.682	0.598	0.442	0.407	0.263	1.471	0.204			
	1.367	1.269	1.064	0.759	0.583	0.483	0.391	0.371	1.500	0.203			
	1.306	1.225	1.045	0.802	0.569	0.472	0.398	0.280	1.458	0.199			
	1.232	1.183	1.001	0.752	0.578	0.497	0.359	0.270	1.354	0.200			
	1.262	1.155	1.060	0.740	0.559	0.460	0.372	0.268	1.361	0.197			
	1.303	1.246	1.133	0.798	0.576	0.453	0.373	0.272	1.501	0.194			
**average**	1.300	1.207	1.043	0.762	0.577	0.467	0.384	0.273	1.448	0.200			
**deviation (SD)**	0.02718	0.03421	0.02885	0.02509	0.00580	0.01324	0.01303	0.00526	0.06028	0.00250	**IC50**	**4.09*10^-7 M**	**(150.103 ng/mL)**
**inhibition rate**	0.11861	0.19275	0.32458	0.54919	0.69806	0.78581	0.85273	0.94170			**SD**	**2.70E-08**	

	drug concentration (M)								Positive	Negative			
**Cell Line**	**2.93E-09**	**5.86E-09**	**1.17E-08**	**2.34E-08**	**4.69E-08**	**9.38E-08**	**1.88E-07**	**3.75E-07**	**Control**	**Control**			
**HTB 131**	1.523	1.410	1.409	1.382	1.360	1.189	0.805	0.676	1.360	0.132			
	1.490	1.452	1.462	1.439	1.356	1.225	0.921	0.750	1.408	0.131			
	1.499	1.466	1.433	1.433	1.360	1.189	0.884	0.686	1.346	0.138			
	1.500	1.467	1.436	1.432	1.355	1.211	0.881	0.705	1.453	0.134			
	1.502	1.436	1.498	1.449	1.255	1.236	0.840	0.694	1.138	0.136			
	1.544	1.428	1.494	1.415	1.414	1.137	0.842	0.658	1.253	0.136			
**average**	1.506	1.446	1.456	1.430	1.358	1.204	0.862	0.679	1.392	0.138			
**deviation (SD)**	0.01140	0.01692	0.02834	0.01031	0.00263	0.01769	0.02401	0.01552	0.04871	0.10100	**IC50**	**1.394*10^-7 M**	**(51.1598 ng/mL)**
**inhibition rate**	-0.09091	-0.04277	-0.05132	-0.03024	0.02705	0.14979	0.42172	0.56754		0.13500	**SD**	**6.20E-09**	

	drug concentration (M)								Positive	Negative			
Cell Line	5.86E-09	1.17E-08	2.34E-08	4.69E-08	9.38E-08	1.88E-07	3.75E-07	7.50E-07	Control	Control			
231	0.809	0.744	0.729	0.569	0.444	0.414	0.412	0.416	0.735	0.183			
	0.810	0.803	0.711	0.566	0.449	0.435	0.418	0.420	0.773	0.173			
	0.822	0.750	0.688	0.587	0.459	0.421	0.417	0.413	0.847	0.179			
	0.839	0.760	0.692	0.584	0.448	0.419	0.417	0.401	0.656	0.179			
	0.785	0.744	0.710	0.534	0.456	0.398	0.405	0.425	0.851	0.174			
	0.717	0.730	0.737	0.520	0.453	0.384	0.399	0.355	0.722	0.172			
average	0.807	0.750	0.711	0.563	0.452	0.413	0.413	0.396	0.802	0.176			
deviation (SD)	0.01550	0.00755	0.01511	0.02103	0.00695	0.01042	0.00568	0.02825	0.05702	0.00320	**IC50**	**7.58*10^-8 M**	**(27.8186 ng/mL)**
inhibition rate	-0.00800	0.08317	0.14554	0.38105	0.55978	0.62135	0.62175	0.64814			**SD**	**7.20E-09**	

	drug concentration (M)								Positive	Negative		
Cell Line	4.26E-07	8.52E-07	1.70E-06	3.41E-06	6.81E-06	1.36E-05	2.73E-05	5.45E-05	Control	Control		
MCF10A	1.759	1.147	0.973	0.953	0.852	0.796	0.775	0.657	1.755	0.121		
	1.491	1.152	0.940	0.878	0.831	0.793	0.741	0.658	1.754	0.188		
	1.396	1.132	0.992	0.871	0.846	0.794	0.792	0.698	1.737	0.199		
	1.539	1.156	0.991	0.871	0.869	0.760	0.729	0.623	1.766	0.202		
	1.355	1.222	0.971	0.890	0.891	0.759	0.699	0.619	1.747	0.207		
	1.719	1.158	0.990	0.889	0.854	0.778	0.795	0.663	1.748	0.208	**IC50**	**3.93E-06 M**
average	1.543	1.161	0.976	0.892	0.857	0.780	0.755	0.653	1.751		**SD**	**5.57E-07**
deviation (SD)	0.16571	0.03122	0.02003	0.03102	0.02064	0.01712	0.03841	0.02906	0.00970			
inhibition rate	0.12761	0.36194	0.47542	0.54964	0.57598	0.62690	0.64502	0.71164				

C1:

	drug concentration (M)								Positive	Negative		
Cell Line	1.24E-08	2.47E-08	4.94E-08	9.88E-08	1.98E-07	3.95E-07	7.90E-07	1.58E-06	Control	Control		
231BR	0.700	0.700	0.741	0.755	0.702	0.472	0.415	0.347	0.728	0.189		
	0.735	0.681	0.752	0.785	0.742	0.482	0.415	0.383	0.720	0.196		
	0.684	0.703	0.719	0.686	0.707	0.464	0.420	0.337	0.674	0.197		
	0.686	0.693	0.727	0.752	0.673	0.492	0.413	0.353	0.691	0.200		
	0.690	0.716	0.707	0.691	0.702	0.480	0.417	0.338	0.685	0.196		
	0.715	0.706	0.727	0.716	0.718	0.483	0.420	0.335	0.736			
average	0.702	0.700	0.729	0.731	0.707	0.479	0.417	0.349	0.706	0.196		
deviation (SD)	0.01993	0.01192	0.01590	0.03945	0.02259	0.00968	0.00288	0.01809	0.02557	0.00404	**IC50**	**5.97E-07 M**
Inhibition Rate	0.00850	0.01209	-0.04477	-0.04869	-0.00261	0.44542	0.56732	0.70033			**SD**	**3.28E-08**

	drug concentration (M)								Positive	Negative		
Cell Line	1.24E-08	2.47E-08	4.94E-08	9.88E-08	1.98E-07	3.95E-07	7.90E-07	1.58E-06	Control	Control		
CRL	1.853	1.930	1.910	1.836	1.128	0.958	0.966	0.939	1.921	0.186		
	1.883	1.892	1.907	1.787	1.121	0.977	0.934	0.994	1.938	0.186		
	1.880	1.880	1.831	1.793	1.196	0.985	0.965	0.917	1.862	0.188		
	1.892	1.910	1.844	1.849	1.189	0.997	0.991	0.961	1.870	0.186		
	1.944	1.924	1.837	1.773	1.184	1.001	0.994	0.938	1.885	0.183		
	1.972	1.872	1.848	1.819	1.150	0.991	0.904	0.943	1.966	0.177		
average	1.904	1.901	1.863	1.810	1.161	0.985	0.959	0.949	1.907	0.184		
deviation (SD)	0.04469	0.02372	0.03586	0.02988	0.03270	0.01568	0.03459	0.02627	0.04127	0.00393	**IC50**	**3.15E-07 M**
Inhibition Rate	0.00174	0.00329	0.02563	0.05659	0.43277	0.53521	0.55020	0.55620			**SD**	**9.77E-09**

	drug concentration (M)								Positive	Negative		
**Cell Line**	**1.24E-08**	**2.47E-08**	**4.94E-08**	**9.88E-08**	**1.98E-07**	**3.95E-07**	**7.90E-07**	**1.58E-06**	**Control**	**Control**		
**HTB131**	1.592	1.416	1.224	1.288	0.733	0.695	0.714	0.499	1.743	0.135		
	1.585	1.328	1.347	1.113	0.651	0.682	0.654	0.500	1.721	0.134		
	1.587	1.550	1.625	1.272	0.674	0.727	0.646	0.546	1.662	0.133		
**average**	1.588	1.431	1.399	1.224	0.686	0.701	0.671	0.515	1.709	0.134		
**deviation (SD)**	0.003606	0.111791	0.205432	0.096749	0.042297	0.023159	0.037166	0.026851	0.041885	0.001000	**IC50**	**2.28E-07 M**
**Inhibition Rate**	0.076825	0.176296	0.197037	0.307725	0.649524	0.639788	0.658836	0.758095			**SD**	**1.11E-08**

	drug concentration (M)								Positive	Negative		
**Cell Line**	**1.24E-08**	**2.47E-08**	**4.94E-08**	**9.88E-08**	**1.98E-07**	**3.95E-07**	**7.90E-07**	**1.58E-06**	**Control**	**Control**		
**231**	0.671	0.640	0.623	0.606	0.667	0.342	0.235	0.241	0.699	0.138		
	0.638	0.663	0.691	0.642	0.617	0.368	0.258	0.266	0.699	0.144		
	0.669	0.686	0.684	0.726	0.658	0.381	0.262	0.261	0.708	0.145		
**average**	0.659	0.663	0.666	0.658	0.647	0.364	0.252	0.256	0.702	0.142		
**deviation (SD)**	0.01850	0.02300	0.03740	0.06158	0.02665	0.01986	0.01457	0.01323	0.00520	0.00379	**IC50**	**4.03E-07 M**
**Inhibition Rate**	0.07619	0.06964	0.06429	0.07857	0.09762	0.60417	0.80417	0.79643			**SD**	**3.71E-08**

	drug concentration (M)					Positive	Negative	Drug		
**Cell Line**	**1.58E-08**	**1.58E-07**	**1.58E-06**	**1.58E-05**	**1.58E-04**	**Control**	**Control**	**Control**		
**MCF10A**	1.286	0.938	0.774	0.209	0.271	1.257	0.181	0.182		
	1.286	0.973	0.715	0.202	0.270	1.261	0.183	0.199		
	1.211	0.901	0.752	0.207	0.247	1.310	0.183	0.289		
**average**	1.261	0.937	0.747	0.206	0.263	1.276				
**deviation (SD)**	0.04330	0.03600	0.02982	0.00361	0.01358	0.02951			**IC50**	**1.0E-06 M**
**Inhibition Rate**	0.01371	0.30957	0.48355	0.97806	0.94180				**SD**	**4.60E-08**

	drug concentration (M)					Positive	Negative	Drug		
**Cell Line**	**1.58E-08**	**1.58E-07**	**1.58E-06**	**1.58E-05**	**1.58E-04**	**Control**	**Control**	**Control**		
**361**	4.163	3.463	2.892	0.726	0.296	3.363	0.156	0.151		
	4.160	3.184	2.707	0.869	0.396	3.133	0.155	0.146		
	4.105	3.344	2.881	0.876	0.343	3.255	0.153	0.159		
	4.107	3.518	2.827	0.914	0.349	3.509	0.155	0.140		
	4.090	3.325	2.603	0.846	0.329	3.056	0.154	0.131		
	4.160	3.119	2.681	0.823	0.369	3.363	0.158			
**average**	4.131	3.326	2.765	0.842	0.347	3.280	0.155			
**deviation (SD)**	0.03358	0.15425	0.11840	0.06465	0.03415	0.16651	0.00172		**IC50**	**6.5E-06 M**
**Inhibition Rate**	-0.27227	-0.01456	0.16475	0.78005	0.93856				**SD**	**7.15E-07**

Ps:

	drug concentration (M)								Positive	Negative		
**Cell Line**	**5.86E-07**	**1.17E-06**	**2.34E-06**	**4.69E-06**	**9.38E-06**	**1.88E-05**	**3.75E-05**	**7.50E-05**	**Control**	**Control**		
**361**	0.1190	0.1250	0.1210	0.1230	0.1360	0.1520	0.1560	0.1810	0.1190			
	1.6020	1.5020	1.5910	1.6170	1.6660	1.9220	2.0710	1.9630	1.5450	0.1370		
	1.5530	1.4840	1.5260	1.5580	1.5010	1.7520	1.9960	1.7860	1.4290	0.1110		
	1.4820	1.4130	1.4110	1.5050	1.4660	1.7860	2.0000	1.9150	1.6230	0.1480		
	1.5660	1.6080	1.4860	1.5240	1.4970	2.1010	2.0590	1.8210	1.4120	0.1440		
	1.7020	1.6850	1.7750	1.8230	1.7150	1.6260	2.1470	2.0420	1.7560	0.1570		
**average**	1.3373	1.3028	1.3183	1.3583	1.3302	1.5565	1.7382	1.6180	1.3140	0.1394		
**deviation (SD)**	0.60118	0.58499	0.59939	0.61607	0.59370	0.70688	0.77706	0.71013	0.59918	0.01744		
**Inhibition Rate**	-0.01986	0.00951	-0.00369	-0.03774	-0.01376	-0.20645	-0.36112	-0.25881			**IC50**	**>7.5E-05 M**

	Increased drug concentration (mg/mL)							Positive	Negative			
**Cell Line**	**3.90E-03**	**7.81E-03**	**1.56E-02**	**3.12E-02**	**6.25E-02**	**1.25E-01**	**2.50E-01**	**Control**	**Control**			
**361**	0.882	0.934	1.046	0.868	0.478	0.628	0.283	0.943	0.181			
	0.890	0.974	1.025	0.836	0.577	0.656	0.316	0.922	0.166			
	0.830	0.943	1.104	0.828	0.563	0.717	0.308	0.941	0.168			
	0.856	0.935	1.021	0.797	0.602	0.668	0.324	0.737	0.174			
	0.815	0.956	1.025	0.806	0.583	0.644	0.314	0.883	0.187			
	0.824	0.948	1.099	0.880	0.579	0.639	0.305	0.903	0.218			
**average**	0.848	0.946	1.049	0.835	0.576	0.652	0.311	0.912	0.182			
**deviation (SD)**	0.026583	0.008813	0.034932	0.025684	0.008699	0.012971	0.005123	0.024918	0.019169	**IC50**	**2.28E-04 M**	**(0.074 mg/mL)**
**Inhibition Rate**	0.087671	-0.045890	-0.187329	0.106164	0.460959	0.356507	0.823630			**SD**	**1.57E-05**	

	drug concentration (M)								Positive	Negative			
**Cell Line**	**5.86E-07**	**1.17E-06**	**2.34E-06**	**4.69E-06**	**9.38E-06**	**1.88E-05**	**3.75E-05**	**7.50E-05**	**Control**	**Control**			
**231BR**	1.065	0.725	0.595	0.592	0.563	0.556	0.499	0.382	1.301	0.203			
	1.087	0.756	0.605	0.601	0.593	0.569	0.516	0.385	1.344	0.205			
	1.049	0.728	0.615	0.612	0.584	0.590	0.525	0.392	1.331	0.202			
	1.104	0.742	0.609	0.611	0.578	0.553	0.542	0.393	1.386	0.208			
	1.131	0.769	0.616	0.587	0.585	0.538	0.485	0.380	1.352	0.208			
	1.095	0.752	0.583	0.583	0.553	0.522	0.474	0.375	1.248	0.196			
**average**	1.088	0.745	0.606	0.598	0.578	0.554	0.506	0.385	1.332	0.205			
**deviation (SD)**	0.016681	0.012477	0.008406	0.010563	0.010149	0.012728	0.017802	0.005252	0.022405	0.002646	**IC50**	**9.63E-07 M**	**(311.95 ng/mL)**
**Inhibition Rate**	0.783370	0.478936	0.356098	0.348780	0.330820	0.309978	0.267627	0.159867			**SD**	**2.83E-08**	

	drug concentration (M)								Positive	Negative			
**Cell Line**	**5.86E-07**	**1.17E-06**	**2.34E-06**	**4.69E-06**	**9.38E-06**	**1.88E-05**	**3.75E-05**	**7.50E-05**	**Control**	**Control**			
**CRL**	1.323	1.165	1.222	0.732	0.464	0.386	0.392	0.465	1.376	0.195			
	1.289	1.115	1.233	0.805	0.475	0.409	0.407	0.480	1.311	0.199			
	1.255	1.167	1.279	0.804	0.522	0.412	0.402	0.474	1.324	0.197			
	1.253	1.077	1.337	0.769	0.514	0.392	0.388	0.465	1.313	0.196			
	1.291	1.069	1.291	0.719	0.496	0.384	0.387	0.476	1.310	0.195			
	1.091	1.138	1.248	0.727	0.464	0.377	0.393	0.473	1.394	0.192			
**average**	1.272	1.124	1.263	0.758	0.487	0.393	0.394	0.472	1.331	0.196			
**deviation (SD)**	0.020817	0.037268	0.026862	0.035935	0.022232	0.011354	0.005909	0.004830	0.030540	0.000957	**IC50**	**4.64E-06 M**	**(1504.70 ng/mL)**
**Inhibition Rate**	0.948029	0.817441	0.939881	0.495265	0.256772	0.173530	0.174411	0.243338			**SD**	**3.20E-07**	

	drug concentration (M)								Positive	Negative			
**Cell Line**	**5.86E-08**	**1.17E-07**	**2.34E-07**	**4.69E-07**	**9.38E-07**	**1.88E-06**	**3.75E-06**	**7.50E-06**	**Control**	**Control**			
**HTB131**	1.369	1.315	1.275	1.253	1.105	0.771	0.537	0.550	1.325	0.162			
	1.441	1.390	1.389	1.312	1.286	0.858	0.620	0.595	1.290	0.168			
	1.409	1.256	1.402	1.340	1.067	0.659	0.667	0.602	1.419	0.152			
	1.581	1.391	1.351	1.361	1.276	0.825	0.714	0.609	1.500	0.145			
	1.469	1.267	1.324	1.235	1.109	0.839	0.651	0.526	1.397	0.174			
	1.441	1.398	1.276	1.174	1.103	0.729	0.592	0.557	1.390	0.170			
**average**	1.440	1.341	1.335	1.285	1.148	0.791	0.633	0.576	1.383	0.149			
**deviation (SD)**	0.024522	0.060698	0.047518	0.049254	0.090201	0.050675	0.033312	0.026293	0.040434	0.179000	**IC50**	**2.81E-06 M**	**(910.16 ng/mL)**
**Inhibition Rate**	1.046811	0.965658	0.960957	0.920074	0.808258	0.516149	0.386549	0.340352		0.159750	**SD**	**1.97E-07**	

	drug concentration (M)								Positive	Negative			
**Cell Line**	**5.86E-08**	**1.17E-07**	**2.34E-07**	**4.69E-07**	**9.38E-07**	**1.88E-06**	**3.75E-06**	**7.50E-06**	**Control**	**Control**			
**231**	0.872	0.859	0.837	0.807	0.692	0.539	0.467	0.400	0.839	0.180			
	0.894	0.855	0.815	0.789	0.757	0.548	0.425	0.396	0.834	0.178			
	0.841	0.814	0.875	0.815	0.725	0.530	0.464	0.408	0.861	0.178			
	0.865	0.819	0.846	0.851	0.635	0.526	0.469	0.408	0.654	0.151			
	0.838	0.825	0.856	0.730	0.679	0.176	0.448	0.417	0.814	0.181			
	0.803	0.810	0.786	0.736	0.629	0.507	0.433	0.389	0.792	0.178			
**average**	0.854	0.828	0.839	0.787	0.683	0.526	0.453	0.398	0.837	0.179			
**deviation (SD)**	0.017029	0.018392	0.016299	0.035538	0.037259	0.013478	0.015727	0.007932	0.019305	0.001000	**IC50**	**1.58E-06 M**	**(510.86 ng/mL)**
**Inhibition Rate**	1.025816	0.986712	1.002278	0.923690	0.765756	0.526955	0.416856	0.333713			**SD**	**7.74E-08**	

	drug concentration (M)								Positive	Negative			
**Cell Line**	**5.86E-07**	**1.17E-06**	**2.34E-06**	**4.69E-06**	**9.38E-06**	**1.88E-05**	**3.75E-05**	**7.50E-05**	**Control**	**Control**			
**MCF10A**	1.187	1.079	0.814	0.819	0.793	0.787	0.693	0.573	1.533	0.182			
	1.244	1.135	0.822	0.813	0.816	0.793	0.673	0.608	1.455	0.186			
	1.317	1.063	0.820	0.831	0.748	0.797	0.689	0.591	1.414	0.186			
	1.234	1.102	0.821	0.794	0.768	0.686	0.653	0.469	1.450	0.180			
	1.244	1.056	0.761	0.752	0.765	0.760	0.636	0.578	1.346	0.184			
	1.341	0.961	0.709	0.737	0.788	0.689	0.627	0.568	1.510	0.186			
**average**	1.261	1.066	0.791	0.791	0.780	0.752	0.662	0.565	1.451	0.184			
**deviation (SD)**	0.057129	0.058924	0.046594	0.038247	0.024172	0.051614	0.027542	0.048952	0.067254	0.002530	**IC50**	**8.75E-06 M**	**(2839.91 ng/mL)**
**Inhibition Rate**	0.149829	0.303867	0.520784	0.520916	0.529861	0.551697	0.622862	0.699684			**SD**	**2.98E-07**	

Section F. Crystallographic Characterization

All the crystallographic data for the 2-trifluoromethansulfonyloxy-4-methoxy-5-[(5-ethyl-2H-pyrrol-2-ylidene)methyl]-1H-pyrrole (**Key Intermediate**) structure reported in this paper have been deposited to the Cambridge Crystallographic Data Centre (CCDC) and can be obtained free of charge via www.ccdc.cam.ac.uk/data_request/cif. CCDC deposition number and all data for the compound can be found in **Tables S5–S10**.

Table S5 | Crystallographic data and structure refinement for the **Key Intermediate**.

	CCDC# 2160302
**Empirical Formula**	$C_{13}H_{13}F_3N_2O_4S$
**Mol. Weight**	350.31
**Temperature / K**	100(2)
**Crystal System**	Monoclinic
**Space Group**	*C2/c*
a, b, c **(Å)**	24.822(11), 4.954(2), 23.601(10)
α, β, γ **(°)**	90, 93.027(5), 90
Volume **(Å^3)**	2898(2)
Z	8
**ρ_{calc} / mg mm^{-3}**	1.606
**μ / mm^{-1}**	0.280
**F(000)**	1440
**Crystal Size / mm^3**	0.350 × 0.095 × 0.010
**2Θ Range for Data Collection**	1.643 to 25.641°
**Index Ranges**	$-30 \le h \le 30$, $-6 \le k \le 6$, $-28 \le l \le 28$
**Reflections Collected**	12659
**Independent Reflections**	2727[R(int) = 0.0530]
**Data/Restraints/Parameters**	2727/0/214
**Goodness-of-fit on F^2**	1.056
**Final R Indexes [I>2σ (I)]**	R_1 = 0.0421, wR_2 = 0.1020
**Final R Indexes [All Data]**	R_1 = 0.0604, wR_2 = 0.1108
**Largest Diff. Peak/Hole / e Å^{-3}**	0.372/-0.492

Crystallographic Data of the Key Intermediate

Method: Single crystals of $C_{13}H_{13}F_3N_2O_4S$ were submitted. A suitable crystal was selected (a Zeiss Stemi 305 microscope was used to identify a suitable specimen) and the crystal was mounted on a MīTiGen holder in Paratone oil on a Bruker Kappa APEX-II CCD diffractometer (operated at 1500 W (50kV, 30 mA) to generate (graphite monochromated) Mo Kα radiation (λ = 0.71073 Å)). The crystal was kept at 100 K during data collection. Using Olex2 (Dolomanov et al., 2009), the structure was solved with the XT (Sheldrick, 2015) structure solution program using Intrinsic Phasing and refined with the XL (Sheldrick, 2007) refinement package using Least Squares minimization.

Crystal Data: For $C_{13}H_{13}F_3N_2O_4S$ (M = 350.31): monoclinic, space group *C2/c* (no. 15), a = 24.822(11) Å, b = 4.954(2) Å, c = 23.601(10) Å, α = 90°, β = 93.027(5)°, γ = 90°, V = 2898(2) Å^3, Z = 8, T = 100(2) K, μ(MoKα) = 0.280 mm^{-1}, *Dcalc* = 1.606 g/mm^3, 12659 reflections measured (1.643° ≤ 2Θ ≤ 25.641°), 2727 unique (R_{int} = 0.0530) which were used in all calculations. The final R_1 was 0.0421 (I > 2σ(I)) and wR_2 was 0.1108 (all data).

Refinement Details: After data collection, the unit cell was re-determined using a subset of the full data collection. Intensity data were corrected for Lorentz, polarization, and background effects using the Bruker program APEX 3. A semi-empirical correction for adsorption was applied using the program *SADABS* (Krause et al., 2014). The *SHELXL-2014* (Sheldrick, 2007), series of programs was used for the solution and refinement of the crystal structure. Hydrogen atoms bound to carbon atoms were located in the difference Fourier map and were geometrically constrained using the appropriate AFIX commands. The hydrogen atom bound to N2 (H2A) was last major peak found in the difference Fourier map and was allowed to refine both its position and thermal displacement parameter.

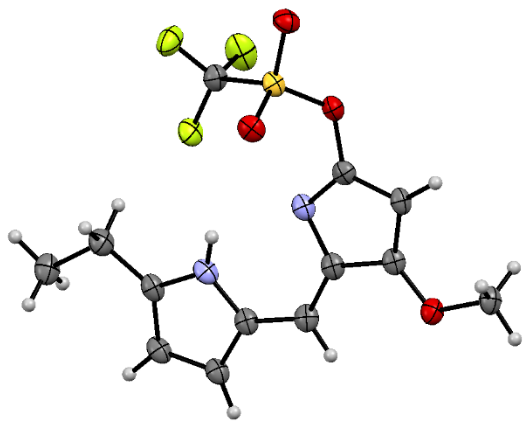

Figure S9|Single crystal X-ray structures of the **Key Intermediate**.

Table S6|Atomic coordinates (x 10^4) and equivalent isotropic displacement parameters (Å^2 x 10^3) for **Key Intermediate**. U(eq) is defined as one third of the trace of the orthogonalized Uij tensor for the **Key Intermediate**.

	**x**	**y**	**z**	**U(eq)**
S(1)	4689(1)	7266(1)	6443(1)	24(1)
F(1)	5300(1)	4339(3)	5793(1)	33(1)
F(2)	4454(1)	4241(3)	5584(1)	42(1)
F(3)	4912(1)	7752(3)	5385(1)	46(1)
O(1)	5079(1)	9725(3)	6507(1)	27(1)
O(2)	4802(1)	5311(4)	6866(1)	29(1)
O(3)	4170(1)	8362(4)	6333(1)	32(1)
O(4)	6701(1)	11156(4)	7681(1)	28(1)
N(1)	5944(1)	7780(4)	6551(1)	25(1)
N(2)	6581(1)	3847(5)	6053(1)	26(1)
C(1)	5607(1)	9460(5)	6756(1)	24(1)
C(2)	5790(1)	11114(5)	7213(1)	24(1)
C(3)	6316(1)	10326(5)	10326(5)	23(1)
C(4)	6418(1)	8219(5)	6892(1)	25(1)
C(5)	6886(1)	6852(5)	6833(1)	26(1)
C(6)	6973(1)	4782(5)	6435(1)	25(1)
C(7)	7441(1)	3366(5)	6330(1)	27(1)
C(8)	7318(1)	1607(5)	5881(1)	28(1)
C(9)	6781(1)	1934(5)	5710(1)	26(1)
C(10)	6441(1)	607(6)	5257(1)	32(1)
C(11)	6748(1)	-1408(6)	4910(1)	34(1)
C(12)	6538(1)	13191(5)	8070(1)	28(1)
C(13)	4859(1)	5817(5)	5759(1)	28(1)

Table S7|Bond lengths [Å] and angles [°] for the **Key Intermediate**.

Bond lengths [Å]	Bond angles [°]	Bond lengths [Å]	Bond angles [°]
S(1)-O(2)	1.4075(18)	N(1)-C(1)-O(1)	120.6(2)
S(1)-O(3)	1.4096(19)	C(2)-C(1)-O(1)	121.2(2)
S(1)-O(1)	1.5589(19)	C(3)-C(2)-C(1)	102.5(2)
S(1)-C(13)	1.836(3)	C(3)-C(2)-H(2B)	128.7
F(1)-C(13)	1.317(3)	C(1)-C(2)-H(2B)	128.7
F(2)-C(13)	1.323(3)	O(4)-C(3)-C(2)	131.0(2)
F(3)-C(13)	1.315(3)	O(4)-C(3)-C(4)	121.5(2)
O(1)-C(1)	1.413(3)	C(2)-C(3)-C(4)	107.6(2)
O(4)-C(3)	1.342(3)	C(5)-C(4)-N(1)	123.9(2)
O(4)-C(12)	1.435(3)	C(5)-C(4)-C(3)	127.3(2)
N(1)-C(1)	1.292(3)	N(1)-C(4)-C(3)	108.8(2)
N(1)-C(4)	1.406(3)	C(4)-C(5)-C(6)	126.2(2)
N(2)-C(9)	1.357(3)	C(4)-C(5)-H(5)	116.9
N(2)-C(6)	1.372(3)	C(6)-C(5)-H(5)	116.9
N(2)-H(2A)	0.79(3)	N(2)-C(6)-C(7)	106.5(2)
C(1)-C(2)	1.410(3)	N(2)-C(6)-C(5)	123.6(2)
C(2)-C(3)	1.369(3)	C(7)-C(6)-C(5)	129.9(2)
C(2)-H(2B)	0.9500	C(6)-C(7)-C(8)	107.4(2)
C(3)-C(4)	1.452(3)	C(6)-C(7)-H(7)	126.3
C(4)-C(5)	1.360(4)	C(8)-C(7)-H(7)	126.3
C(5)-C(6)	1.415(4)	C(9)-C(8)-C(7)	108.6(2)
C(5)-H(5)	0.9500	C(9)-C(8)-H(8)	125.7
C(6)-C(7)	1.391(3)	C(7)-C(8)-H(8)	125.7
C(7)-C(8)	1.393(4)	N(2)-C(9)-C(8)	106.6(2)
C(7)-H(7)	0.9500	N(2)-C(9)-C(10)	121.9(2)
C(8)-C(9)	1.382(4)	C(8)-C(9)-C(10)	131.6(2)
C(8)-H(8)	0.9500	C(9)-C(10)-C(11)	113.4(2)
C(9)-C(10)	1.481(4)	C(9)-C(10)-H(10A)	108.9
C(10)-C(11)	1.523(4)	C(11)-C(10)-H(10A)	108.9
C(10)-H(10A)	0.9900	C(9)-C(10)-H(10B)	108.9
C(10)-H(10B)	0.9900	C(11)-C(10)-H(10B)	108.9
C(11)-H(11A)	0.9800	H(10A)-C(10)-H(10B)	107.7
C(11)-H(11B)	0.9800	C(10)-C(11)-H(11A)	109.5
C(11)-H(11C)	0.9800	C(10)-C(11)-H(11B)	109.5
C(12)-H(12A)	0.9800	H(11A)-C(11)-H(11B)	109.5
C(12)-H(12B)	0.9800	C(10)-C(11)-H(11C)	109.5
C(12)-H(12C)	0.9800	H(11A)-C(11)-H(11C)	109.5
O(2)-S(1)-O(3)	122.87(11)	H(11B)-C(11)-H(11C)	109.5

O(2)-S(1)-O(1)	111.72(11)	O(4)-C(12)-H(12A)	109.5
O(3)-S(1)-O(1)	105.90(11)	O(4)-C(12)-H(12B)	109.5
O(2)-S(1)-C(13)	107.88(12)	H(12A)-C(12)-H(12B)	109.5
O(3)-S(1)-C(13)	103.76(12)	O(4)-C(12)-H(12C)	109.5
O(1)-S(1)-C(13)	102.68(11)	H(12A)-C(12)-H(12C)	109.5
C(1)-O(1)-S(1)	121.66(15)	H(12B)-C(12)-H(12C)	109.5
C(3)-O(4)-C(12)	115.28(19)	F(3)-C(13)-F(1)	109.5(2)
C(1)-N(1)-C(4)	103.0(2)	F(3)-C(13)-F(2)	108.8(2)
C(9)-N(2)-C(6)	111.0(2)	F(1)-C(13)-F(2)	107.9(2)
C(9)-N(2)-H(2A)	121(2)	F(3)-C(13)-S(1)	109.97(18)
C(6)-N(2)-H(2A)	127(2)	F(1)-C(13)-S(1)	113.17(18)
N(1)-C(1)-C(2)	118.1(2)	F(2)-C(13)-S(1)	107.43(17)

Symmetry transformations used to generate equivalent atoms

Table S8|Anisotropic displacement parameters ($Å^2 \times 10^3$) for the **Key Intermediate**. The anisotropic displacement factor exponent takes the form: $-2\pi^2 [h^2 a^{*2}U^{11} + ... + 2 h k a^* b^* U^{12}]$

	**U11**	**U22**	**U33**	**U23**	**U13**	**U12**
S(1)	26(1)	26(1)	21(1)	-1(1)	10(1)	2(1)
F(1)	30(1)	37(1)	31(1)	-9(1)	11(1)	5(1)
F(2)	33(1)	51(1)	43(1)	-22(1)	3(1)	-2(1)
F(3)	74(1)	43(1)	23(1)	7(1)	16(1)	6(1)
O(1)	30(1)	23(1)	28(1)	-2(1)	4(1)	3(1)
O(2)	30(1)	32(1)	24(1)	6(1)	10(1)	1(1)
O(3)	27(1)	36(1)	33(1)	-2(1)	5(1)	7(1)
O(4)	26(1)	32(1)	26(1)	-6(1)	8(1)	-2(1)
N(1)	27(1)	24(1)	23(1)	1(1)	11(1)	0(1)
N(2)	24(1)	30(1)	26(1)	0(1)	12(1)	4(1)
C(1)	27(1)	24(1)	21(1)	4(1)	5(1)	-1(1)
C(2)	29(1)	22(1)	21(1)	-1(1)	11(1)	0(1)
C(3)	28(1)	24(1)	18(1)	2(1)	11(1)	-1(1)
C(4)	28(1)	24(1)	23(1)	2(1)	11(1)	-2(1)
C(5)	27(1)	28(1)	24(1)	1(1)	12(1)	-4(1)
C(6)	26(1)	25(1)	25(1)	2(1)	12(1)	-2(1)
C(7)	26(1)	26(1)	30(1)	0(1)	10(1)	1(1)
C(8)	27(1)	28(1)	29(1)	1(1)	13(1)	3(1)
C(9)	31(1)	24(1)	23(1)	1(1)	12(1)	2(1)
C(10)	30(1)	36(2)	31(2)	-5(1)	9(1)	3(1)
C(11)	34(2)	37(2)	31(2)	-8(1)	15(1)	-4(1)
C(12)	31(1)	28(1)	26(1)	-5(1)	9(1)	-2(1)
C(13)	32(1)	30(2)	24(1)	-1(1)	7(1)	0(1)

Table S9|Hydrogen coordinates (x 10^4) and isotropic displacement parameters (Å^2 x 10^3) for the **Key Intermediate**.

	x	y	z	U(eq)
H(2A)	6282(12)	4380(60)	6006(12)	28(8)
H(2B)	5596	12441	7410	28
H(5)	7184	7333	7082	31
H(7)	7782	3562	6528	32
H(8)	7562	388	5720	33
H(10A)	6283	2010	5000	38
H(10B)	6140	-340	5432	38
H(11A)	6504	-2186	4613	51
H(11B)	6893	-2849	5158	51
H(11C)	7046	-485	4734	51
H(12A)	6843	13661	8331	43
H(12B)	6241	12502	8286	43
H(12C)	6418	14800	7857	43

Table S10|Hydrogen bonds for the **Key Intermediate**.

D-H	d(D-H)	d(H..A)	<DHA	d(D..A)	A
N2-H2A	0.791	2.461	159.77	3.215	F1
N2-H2A	0.791	2.304	122.23	2.806	N1

Section G. References

Cetin, M. M. (2017). Syntheses and characterization of copper(I) complexes for study of dynamic supramolecular ring-chain equilibria and application as photoredox catalysts. *PhD Dissertation*. Texas Tech University, Lubbock, TX, USA

Cetin, M. M., Hodson, R. T., Hart, C. R., Cordes, D. B., Findlater, M., Casadonte Jr., D. J., et al. (2017). Characterization and photocatalytic behavior of 2,9-di(aryl)-1,10-phenanthroline copper (I) complexes. *Dalton Trans.* 46 (20), 6553–6569. doi:10.1039/c7dt00400a

Cetin, M. M., Shafiei-Haghighi, S., Chen, J., Zhang, S., Miller, A. C., Unruh, D. K., et al. (2020). Synthesis, structures, photophysical properties, and catalytic characteristics of 2,9-dimesityl-1,10-phenanthroline (dmesp) transition metal complexes. *J. Polym. Sci.* 58 (8), 1130–1143. doi:10.1002/pol.20190276

Dietrich-Buchecker, C. and Sauvage, J.-P. (1990). Templated synthesis of interlocked macrocyclic ligands, the catenands. Preparation and characterization of the prototypical bis-30 membered ring system. *Tetrahedron*, 46 (2), 503–512. doi:10.1016/s0040-4020(01)85433-8

Dolomanov, O. V., Bourhis, L. J., Gildea, R. J., Howard, J. A. K. and Puschmann, H. (2009). OLEX2: a complete structure solution, refinement and analysis program. *J. Appl. Cryst.* 42 (2), 339–341. doi:10.1107/s0021889808042726

Hayes, D., Kohler, L., Chen, L. X. and Mulfort, K. L. (2018). Ligand Mediation of Vectorial Charge Transfer in Cu(I)diimine Chromophore–Acceptor Dyads. *J. Phys. Chem. Lett.* 9 (8), 2070–2076. doi:10.1021/acs.jpclett.8b00468

Hayes, D., Kohler, L., Hadt, R. G., Zhang, X., Liu, C., Mulfort, K. L., et al. (2018). Excited state electron and energy relays in supramolecular dinuclear complexes revealed by ultrafast optical and X-ray transient absorption spectroscopy. *Chem. Sci.* 9 (4), 860–875. doi:10.1039/c7sc04055e

Kang, S., Berkshire, B. M., Xue, Z., Gupta, M., Layode, C., May, P. A., et al. (2008). Polypseudorotaxanes via Ring-Opening Metathesis Polymerizations of [2]Catenanes. *J. Am. Chem. Soc.* 130 (46), 15246–15247. doi:10.1021/ja806122r

Kang, S., Cetin, M. M., Jiang, R., Clevenger, E. S. and Mayer, M. F. (2014). Synthesis of Metalated Pseudorotaxane Polymers with Full Control over the Average Linear Density of Threaded Macrocycles. *J. Am. Chem. Soc.* 136 (36), 12588–12591. doi:10.1021/ja507167k

Kohler, L., Hadt, R. G., Hayes, D., Chen, L. X. and Mulfort, K. L. (2017). Synthesis, structure, and excited state kinetics of heteroleptic Cu(I) complexes with a new sterically demanding phenanthroline ligand. *Dalton Trans.* 46 (38), 13088–13100. doi:10.1039/c7dt02476b

Kohler, L., Hayes, D., Hong, J., Carter, T. J., Shelby, M. L., Fransted, K. A., et al. (2016). Synthesis, structure, ultrafast kinetics, and light-induced dynamics of CuHETPHEN chromophores. *Dalton Trans.* 45 (24), 9871–9883. doi:10.1039/c6dt00324a

Krause, L., Herbst-Irmer, R., Sheldrick, G. M., Zachariae, U., de Groot, B. L. (2014). SADABS v2014/5. *J. Appl. Crsyt.* 48, 3–10. https://doi.org/10.1107/S1600576714022985

Melvin, M. S., Tomlinson, J. T., Park, G., Day, C. S., Saluta, G. R., Kucera, G. L., et al. (2002). Influence of theA-Ring on the Proton Affinity and Anticancer Properties of the Prodigiosins. *Chem. Res. Toxicol.* 15 (5), 734–741. doi:10.1021/tx025507x

Schmittel, M., Lüning, U., Meder, M., Ganz, A., Michel, C. and Herderich, M. (1997). Synthesis of sterically encumbered 2,9-diaryl substituted phenanthrolines. Key building blocks for the preparation of mixed (Bis-heteroleptic) phenanthroline copper(I) complexes. *Heterocycl. Commun.* 3 (6)**,** 493–498. doi:10.1515/hc.1997.3.6.493

Sheldrick, G. M. (2007). A short history ofSHELX. *Acta Crystallogr., Sect. A Fundam. Cryst.* 64 (1), 112–122. doi:10.1107/s0108767307043930

Sheldrick, G. M. (2015). SHELXT-Integrated space-group and crystal-structure determination. *Acta Crystallogr., Sect. A: Found Adv.* 71 (1), 3–8. doi:10.1107/s2053273314026370

Zhong, W., Tang, Y., Zampella, G., Wang, X., Yang, X., Hu, B., et al. (2010). A rare bond between a soft metal (Fe^{I}) and a relatively hard base (RO^{-}, R = phenolic moiety). *Inorg. Chem. Commun.* 13 (9), 1089–1092. doi:10.1016/j.inoche.2010.06.026
